# Early Retinal UCHL1 Dysregulation Coupled With Synaptic Loss Reflects Alzheimer's Disease Severity

**DOI:** 10.1002/advs.202600020

**Published:** 2026-08-29

**Authors:** Altan Rentsendorj, Jean‐Philippe Vit, Alexandre Hutton, Yosef Koronyo, Saba Shahin, Edward Robinson, Ernesto Barron, Anthony Rodriguez, Ousman Jallow, Julia Sheyn, Bhakta Prasad Gaire, Lalita Subedi, Miyah R. Davis, Barathan Gnanabharathi, Alexander V. Ljubimov, Stuart L. Graham, Vivek K. Gupta, Mehdi Mirzaei, Debra Hawes, Katlin Silm, Keith L. Black, Lon S. Schneider, Alfredo A. Sadun, Jesse G. Meyer, Dieu‐Trang Fuchs, Maya Koronyo‐Hamaoui

**Affiliations:** ^1^ Department of Neurosurgery Maxine Dunitz Neurosurgical Institute Cedars‐Sinai Medical Center Los Angeles California USA; ^2^ Department of Computational Biomedicine Cedars‐Sinai Medical Center Los Angeles California USA; ^3^ Doheny Eye Institute University of California Los Angeles California USA; ^4^ Board of Governors Regenerative Medicine Institute Cedars‐Sinai Medical Center Los Angeles California USA; ^5^ Division of Infectious Diseases and Immunology Department of Pediatrics Guerin Children's at Cedars‐Sinai Medical Center Los Angeles California USA; ^6^ Department of Biomedical Sciences Infectious and Immunologic Diseases Research Center Cedars‐Sinai Medical Center Los Angeles California USA; ^7^ Center For Neural Science and Medicine Cedars‐Sinai Medical Center Los Angeles California USA; ^8^ Department of Biomedical Sciences Division of Applied Cell Biology and Physiology Cedars‐Sinai Medical Center Los Angeles California USA; ^9^ Department of Neurology Cedars‐Sinai Medical Center Los Angeles California USA; ^10^ David Geffen School of Medicine University of California Los Angeles California USA; ^11^ Macquarie Medical School Faculty of Medicine Health and Human Sciences Macquarie University Sydney NSW Australia; ^12^ ProHeme Diagnostics Pty Ltd Sydney NSW Australia; ^13^ Department of Pathology Keck School of Medicine University of Southern California Los Angeles California USA; ^14^ Department of Psychiatry and The Behavioral Sciences Department of Neurology Keck School of Medicine University of Southern California Los Angeles California USA; ^15^ Smidt Heart Institute Cedars‐Sinai Medical Center Los Angeles California USA

**Keywords:** dementia, excitatory synapse, MCI, N‐methyl‐D‐aspartate receptor subunit 2A, p75NTR, postsynaptic density protein 95, ubiquitin–proteasome system, vesicular glutamate transporter 1

## Abstract

Synaptic dysfunction is a major driver of cognitive decline in Alzheimer's disease (AD), yet its extent and molecular basis in the retina remain poorly defined. We integrated postmortem retinal and matched brain histopathology with ultrastructural, proteomic, biochemical, and machine‐learning analyses across cognitively normal, mild cognitive impairment, and AD cohorts. Retinal glutamatergic synapses exhibited early, progressive degeneration, marked by loss of presynaptic vesicular glutamate transporter 1 (VGLUT1) and synaptophysin and postsynaptic density protein 95 (PSD95) and N‐methyl‐D‐aspartate receptor subunit 2A (NMDAR2A), along with ribbon synapse ultrastructural disruption. Synaptic deficits correlated with amyloid‐β_42_ (Aβ_42_), pathogenic tau, oxidative stress, the Aβ‐binding p75 neurotrophin receptor, and glial activation that paralleled disease progression. Proteomics revealed widespread synaptic remodeling accompanied by disease‐associated microglia, astrocyte‐mediated excitotoxicity, and pyroptotic pathways. The synapse‐enriched deubiquitinase ubiquitin C‐terminal hydrolase L1 (UCHL1) was dysregulated early, particularly in horizontal and bipolar interneurons, and strongly associated with synaptic loss and neuroinflammation. Mechanistically, fibrillar Aβ_42_ induced rapid UCHL1 and synaptic depletion in human and murine neurons before overt neurodegeneration. Machine‐learning models identified retinal UCHL1 as the strongest predictor of Braak stage and cognitive impairment. These findings establish the retina as an early site of AD synaptopathy and position UCHL1 as a candidate biomarker and mechanistic mediator linking amyloid pathology, neuroinflammation, and synaptic vulnerability.

## Graphical abstract

This study identifies synapse‐enriched deubiquitinase UCHL1 as an early Aβ‐responsive regulator of retinal synaptopathy in Alzheimer's disease. Retinal UCHL1 loss accompanies excitatory synapse degeneration, p75NTR activation, and neuroinflammation, and predicts Braak stage and cognitive decline. Aβ_42_ fibrils trigger synaptic and UCHL1 depletion before overt neurodegeneration, positioning UCHL1 as a mechanistic mediator and promising retinal biomarker for AD.

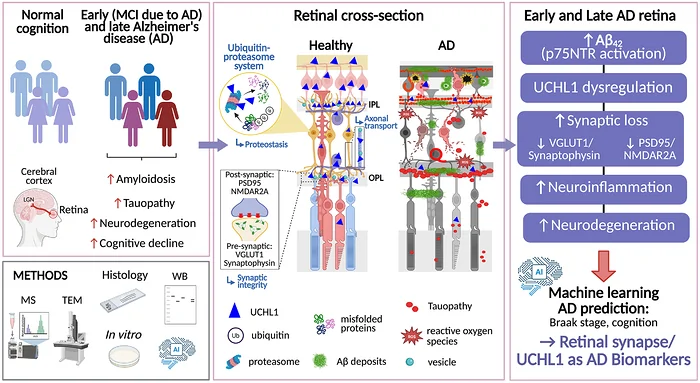

## 1 Introduction

Synaptopathy is an early and central feature of Alzheimer's disease (AD) and one of the strongest predictors of cognitive decline [1, 2, 3]. Traditionally characterized by amyloid β‐protein (Aβ) plaques and neurofibrillary tangles (NFTs) composed of hyperphosphorylated (p)tau protein [4, 5, 6, 7, 8, 9], AD is increasingly recognized as a brain disorder driven by early synaptic dysfunction, with deficits in glutamatergic signaling commonly preceding neuronal loss [2, 10, 11, 12, 13, 14, 15, 16, 17]. We previously found that overnight exposure to fibrillar, and moreover, oligomeric Aβ_42_ aggregates caused a 50%–70% loss of glutamatergic pre‐ and postsynaptic puncta in mouse primary cortical neuron cultures [18]. However, despite decades of study, the mechanisms linking Aβ and tau accumulation to synaptic collapse and neurodegeneration remain unclear, underscoring the need for accessible biomarkers that faithfully reflect these pathological processes.

Brain synaptic density can be quantified in vivo using synaptic vesicle glycoprotein 2A (SV2A) positron emission tomography (PET) imaging with [^11^C]UCB‐J, and synaptic proteins can be measured in cerebrospinal fluid (CSF); yet, these approaches are limited by exposure to radioactive tracers, high cost and limited accessibility, and the requirement for invasive lumbar puncture procedures, underscoring the need for safer and more practical biomarkers of synaptic integrity.

The neurosensory retina, an embryological outgrowth of the brain, provides a uniquely accessible site for noninvasive imaging of central nervous system (CNS) pathology [19]. Increasing evidence demonstrates that AD hallmark pathologies, including Aβ deposits, tau inclusions, gliosis, and vascular changes, are detectable in the human retina and correlate with cerebral pathology and cognitive decline [19, 20, 21, 22, 23, 24, 25, 26, 27, 28, 29, 30, 31, 32, 33, 34, 35, 36, 37, 38, 39]. Advanced retinal imaging modalities, such as hyperspectral imaging and scanning laser ophthalmoscopy, have further detected Aβ and vascular dysfunction in living patients [27, 40, 41, 42, 43, 44, 45, 46, 47, 48, 49, 50, 51]. Retinal synaptic dysfunction in living humans is primarily assessed noninvasively using electroretinography (ERG), particularly pattern ERG (PERG) and multifocal ERG (mfERG), which enable detection of functional deficits preceding overt structural degeneration [52]. Optical coherence tomography (OCT) is commonly integrated with ERG to identify corresponding structural alterations, including thinning of synapse‐rich inner retinal layers such as the inner plexiform layer (IPL) and ganglion cell–inner plexiform layer (GCIPL), the latter serving as a robust in vivo marker of retinal ganglion cell dysfunction [53, 54, 55]. In research settings, adaptive optics (AO) imaging combined with OCT or scanning laser ophthalmoscopy (SLO) provides cellular‐resolution visualization of photoreceptors and retinal microarchitecture [56, 57]. However, whether synapses in the AD retina undergo degeneration that parallels neurodegeneration in the brain, and the molecular mechanisms that govern this process, remain largely unexplored.

Over the last decade, neuroinflammation has shifted from a secondary correlate of AD to a primary and early driver of its pathogenesis, with microglia and astrocytes influencing amyloid handling, cytokine signaling, oxidative stress, and synaptic remodeling and elimination [58, 59]. Dysregulated innate immune processes, rather than simply responding to neuronal injury, directly contribute to synaptic dysfunction, circuit destabilization, and disease progression [58, 60, 61, 62, 63, 64, 65]. Large‐scale genetic and transcriptomic studies have defined disease‐associated microglia (DAM)—also known as neurodegenerative microglia (MGnD)—marked by altered apolipoprotein E (APOE), glycoprotein non‐metastatic melanoma protein B (GPNMB), integrin subunit alpha X (ITGAX), and triggering receptor expressed on myeloid cells 2 (TREM2) expression, that arise during AD‐like progression and tightly associate with cerebral amyloid pathology and neurodegeneration [66, 67]. The human retina, with its own specialized neuroimmune network, exhibits analogous inflammatory signatures in AD, including ionized calcium‐binding adapter molecule 1 (IBA1)‐positive microglial activation, reactive astrocytes and Müller glia [expressing glial fibrillary acidic protein (GFAP), S100 calcium‐binding proetin B (S100β), and/or vimentin], complement dysregulation, and cytokine induction [24, 28, 32, 38, 39, 68, 69]. Our recent work demonstrated that retinal inflammatory activation tracks retinal amyloidosis and neurodegeneration, suggesting that the retina parallels brain immune dysregulation [28, 68, 70]. However, whether retinal neuroinflammation contributes to retinal synaptic degeneration and proteostatic failure in AD remains unknown.

One candidate molecule potentially integrating these processes is ubiquitin carboxyl‐terminal hydrolase L1 (UCHL1; also known as PGP9.5 and PARK5), a neuron‐enriched deubiquitinating enzyme and key component of the ubiquitin–proteasome system (UPS), essential for maintaining ubiquitin homeostasis, synaptic vesicle cycling, neuronal survival, and proteostasis [71, 72, 73, 74]. Beyond its established role in synaptic maintenance, UCHL1 is highly susceptible to oxidative and inflammatory stress and exhibits abnormal oxidation, aggregation, Aβ and tau accumulation, and loss of function in AD brains [75, 76, 77, 78, 79, 80, 81, 82]. Indeed, cerebral UCHL1 was shown to facilitate clearance of misfolded proteins and prevent aggregation of toxic Aβ_42_ and ptau [73, 83, 84, 85]. Reduced UCHL1 expression in AD cortex and hippocampus correlates with synaptic loss and cognitive decline [79]. Whether retinal UCHL1 deficiency occurs within a broader inflammatory microenvironment and how such alterations relate to synaptic degeneration is unresolved.

Another AD‐relevant neurodegenerative pathway involves signaling via the p75 neurotrophin receptor (p75NTR; also known as NGFR). Initially characterized as a death receptor, p75NTR regulates axonal pruning, synaptic plasticity, and apoptosis in the CNS [86, 87, 88, 89, 90]. In brains from AD donors and rodent models, Aβ was shown to bind to p75NTR, driving neuritic degeneration, tau hyperphosphorylation, oxidative stress, and neuronal loss [91, 92, 93, 94, 95, 96]. Furthermore, increased retinal p75NTR has been reported in experimental retinopathy models [97, 98, 99]; however, its relevance to AD retinal pathology and its potential relationship to synaptic failure is not clear. It is therefore hypothesized that, much like the brain, the retina is an early site of synaptic vulnerability in AD, driven by a convergent Aβ and tau pathogenic cascade acting through p75NTR signaling, proteostatic failure, as well as harmful neuroinflammation and UCHL1 dysregulation.

Here, we combine spatially resolved histopathology of supero‐ and infero‐temporal (ST, IT) retinal cross‐sections with ultrastructural, biochemical, proteomic, and machine‐learning approaches across independent cohorts spanning normal cognition (NC), mild cognitive impairment (MCI due to AD), and AD dementia. Focusing on excitatory glutamatergic circuitry, we quantify presynaptic vesicular glutamate transporter 1 (VGLUT1) and synaptophysin (SYP), together with postsynaptic density protein 95 (PSD95) and N‐methyl‐D‐aspartate receptor subunit 2A (NMDAR2A; also known as GluN2A), key regulators of excitatory neurotransmission that are highly vulnerable in AD brains [100, 101, 102, 103]. We reveal early and progressive degeneration of retinal pre‐ and postsynaptic compartments accompanied by ultrastructural synaptic collapse, photoreceptor vulnerability, neuroinflammatory activation, and profound dysregulation of the proteostatic regulator UCHL1 in MCI and AD cases. These abnormalities are tightly associated with retinal Aβ_42_ accumulation, pathogenic tau species, oxidative stress, p75NTR signaling, disease‐associated innate immune pathways, and cognitive decline. Furthermore, machine‐learning analyses identify UCHL1 as the strongest retinal predictor of Braak stage and cognitive status. Mechanistically, Aβ_1–42_ fibrils (fAβ_42_) induced early UCHL1 depletion accompanied by loss of synaptic markers in both human SH‐SY5Y neuroblastoma cells and primary murine hippocampal neurons, preceding detectable neuronal degeneration. Collectively, our findings establish the retina as an early site of neuroinflammatory, synaptic, and proteostatic dysfunction in AD and identify UCHL1 as a potential mechanistic hub linking amyloid pathology, innate immune activation, and neuronal vulnerability.

## 2 Results

**FIGURE 1:**
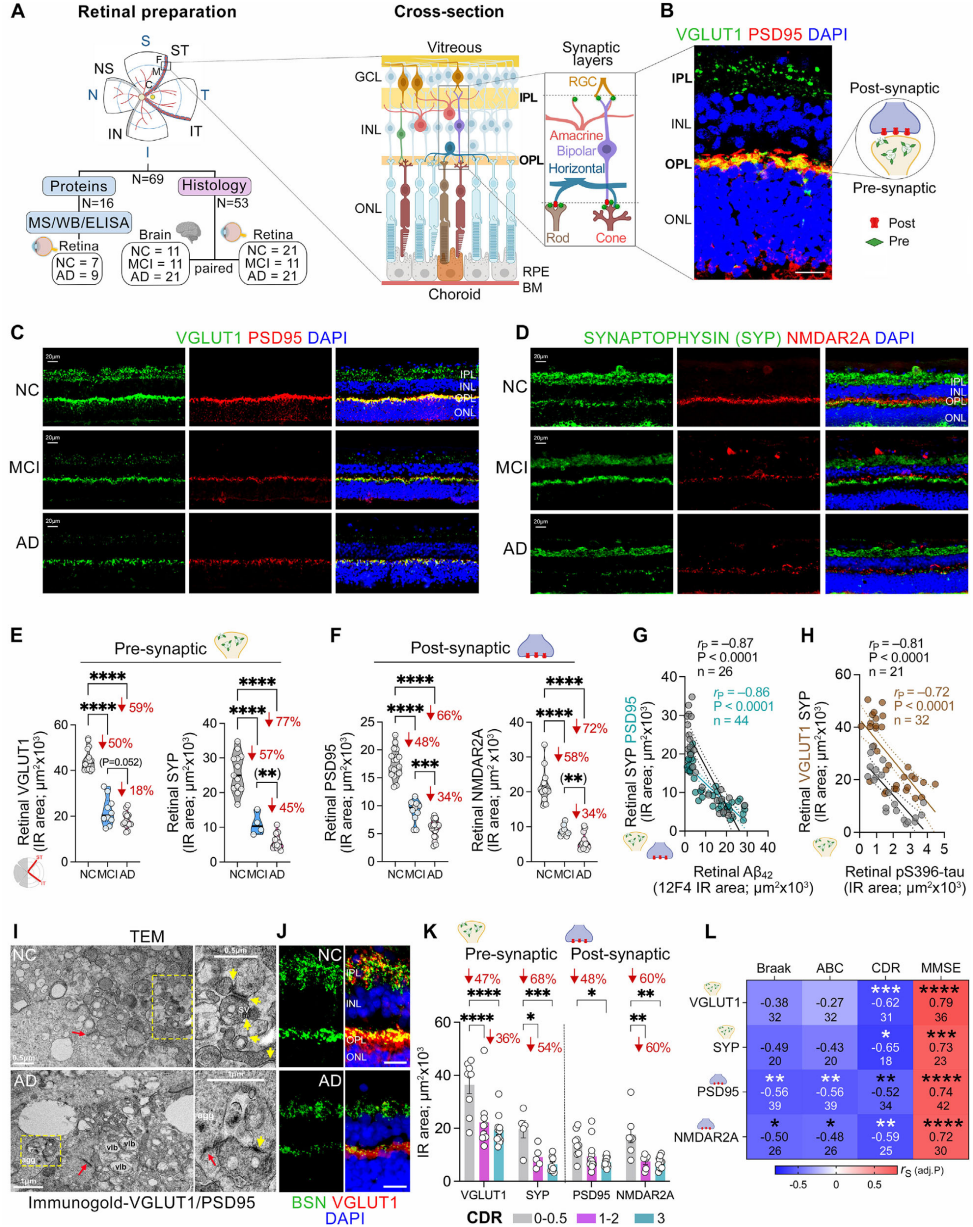
Synaptic loss in MCI and AD retinas and its relationship to core AD pathologies and disease status. (A) Schematic of retinal cross‐sections from NC, MCI, and AD donors in superior‐ and inferior‐temporal (ST/IT) regions (red), subdivided into central (C), mid (M), and far‐peripheral (F) subregions. Retinal layers and synaptic architecture in the inner/outer plexiform layers (IPL/OPL), including pre‐ and postsynaptic terminals, are illustrated. The flowchart indicates allocation of donor eyes for histopathology and biochemical analyses. (B) Representative confocal image immunolabeled for pre‐ (VGLUT1, green) and postsynaptic (PSD95, red) markers, showing strong labeling in the IPL and OPL. Nuclei are stained with DAPI (blue). Scale bar: 20 µm. (C, D) Immunofluorescence images of NC, MCI, and AD retinas stained for pre‐ (VGLUT1, synaptophysin [SYP]; green) and postsynaptic (PSD95, NMDAR2A; red) markers. Nuclei: DAPI (blue). Scale bars: 20 µm. (E, F) Quantification of VGLUT1, SYP, PSD95, and NMDAR2A immunoreactive (IR) area in MCI (*n* = 4–11) and AD (*n* = 12–20) versus NC (*n* = 13–18). (G, H) Pearson's correlations (*r*_P_) between retinal synaptic markers and retinal Aβ_42_ (12F4) (G) or pS396‐tau burden (H). (I) TEM images of the OPL in NC and AD retinas, immunogold‐labeled for VGLUT1 and PSD95. Yellow arrowheads indicate synaptic clefts between pre‐ and postsynaptic terminals. Presynaptic terminals with electron‐dense synaptic vesicles (SVs) are marked. Red arrows highlight membrane‐docked presynaptic ribbons, preserved in NC but detached/floating in AD. agg, electron‐dense aggregate; vlb, blebbing leakage. Scale bar: 0.5–1 µm. (J) Immunofluorescence for synaptic ribbons by Bassoon (green) with VGLUT1 (red) and nuclei (DAPI, blue). Scale bars: 20 µm. (K) Quantification of retinal synaptic markers stratified by clinical dementia rating (CDR) scores (CDR 0–0.5: *n* = 5–9; CDR 1–2: *n* = 5–12; CDR 3: *n* = 8–13). (L) Heatmap of Spearman correlations (*r*_S_) and significance (Holm–Šídák–adjusted *p* values) for each retinal neuronal marker against four brain pathology and cognitive scores (BRAAK, ABC, MMSE, CDR). Violin plots show individual subjects (circles) with lower, median, and upper quartiles. Bar graphs show group means ± SEM; *p < 0.05, **p < 0.01, ***p < 0.001, ****p < 0.0001, by two‐sided unpaired *t*‐test (E, F; values in parentheses), or one‐way (E, F) or two‐way (K) ANOVA followed by Tukey's multiple‐comparisons test. Percentage (%) changes are shown in red. IPL, inner plexiform layer; INL, inner nuclear layer; OPL, outer plexiform layer; ONL, outer nuclear layer. Panel A was created in BioRender.com.

To investigate synaptic integrity and associated neurodegeneration in postmortem retinas from patients with early and advanced‐stage AD, we analyzed the spatiotemporal distribution and burden of synaptic proteins in superior‐ and inferior‐temporal (ST/IT) retinal cross‐sections from a cohort of 53 individuals comprising NC controls (*n* = 21, mean age 82.6 ± 10.5 years, 11 females/10 males), MCI due‐to‐AD (*n* = 11, mean age 89.3 ± 5.2 years, 7 females/4 males), and AD dementia (*n* = 21, mean age 86.2 ± 8.9 years, 13 females/8 males). Age, sex, and postmortem interval (PMI) did not differ significantly among the three diagnostic groups, and these variables were controlled for in each retinal biomarker subset, minimizing potential confounding effects. An additional cohort was used for mass spectrometry (MS)‐based proteomics, Western blot (WB), and enzyme‐linked immunosorbent assay (ELISA) to assess dysregulation of synaptic and AD‐related proteins in the temporal retina of AD patients and NC controls (*n* = 16). Demographic, clinical, and neuropathological data for the cohorts used in histological and protein analyses are detailed in Figure 1A and Table 1 (individual human donors are listed in Table S1).

**TABLE 1:** Demographic and neuropathological data on human brains and retinas in this study.

Histology cohort		NC	MCI	AD	*F*	P
*n* = 53 [F (%), M]		21 [11F (52%), 10 M]	11 [7F (64%), 4 M]	21 [13F (62%), 8 M]	−	−
Age at death (years)		82.6 ± 10.5	89.3 ± 5.2	86.2 ± 8.9	2.11	0.13
Race		16 W (76%), 2B, 3H	9 W (82%), 1B, 1H	15 W (71%), 4A, 2H	−	−
PMI (h)		7.8 ± 4.0	8.2 ± 4.9	7.8 ± 3.6	0.05	0.95
MMSE score (*n* = 46)		28.8 ± 2.0	21 ± 7.2	12.5 ± 7.6	33.0	**<0.0001**
CDR score (*n* = 36)		0.2 ± 0.4	2.1 ± 1.2	2.1 ± 0.9	15.8	**<0.0001**
**Brain neuropathology**	Braak stage (%)	0–II 73%	0–II 27%	0–II 0%	24.4	**<0.0001**
(*n* = 43)		III–IV 18%	III–IV 45%	III–IV 24%		
		V–VI 9%	V–VI 27%	V–VI 76%		
	ABC average	1.4 ± 0.8	2.1 ± 0.5	2.8 ± 0.3	24.6	**<0.0001**
	Aβ plaque^a^	1.1 ± 1.1	1.90 ± 0.8	2.6 ± 1.0	9.2	**0.0005**
	NFTs^a^	0.5 ± 0.5	1.6 ± 1.0	2.3 ± 1.1	13.5	**<0.0001**
	NTs^a^	0.4 ± 0.8	1.1 ± 0.8	1.2 ± 0.8	3.7	**0.036**
	Atrophy^a^	0.5 ± 0.7	1.1 ± 0.9	1.7 ± 1.1	5.7	**0.0067**
**Protein biochemistry cohort**		**NC**		**AD**		**P**
*n* = 16 [F (%), M]		7 [4F (57%), 3 M]		9 [6F (67%), 3 M]		−
Age at death (years)		76.7 ± 5.4		82.0 ± 15.7		0.41
Race		6 W (86%), 1H		8 W (89%), 1H		−
PMI (h)		7.5 ± 4.2		6.6 ± 2.6		0.67
MMSE score (*n* = 4)		23.0 ± n.a.		10.7 ± 6.1		0.22
CDR score (*n* = 7)		0.3 ± 0.4		2.4 ± 0.9		**0.025**
**Brain neuropathology**	Braak stage (%)	I–II (100%)		III (14%)		**0.0013**
(*n* = 9)		−		V–VI (86%)		
	ABC average	1.7 ± 0.5		2.6 ± 0.5		**0.044**
	Aβ plaque^a^	1.7 ± 0.7		2.8 ± 1.0		0.25
	NFTs^a^	1.2 ± 0.007		2.7 ± 1.1		0.088
	NTs^a^	0.2 ± 0.1		1.3 ± 0.7		0.074
	Atrophy^a^	0.6 ± 0.07		1.2 ± 1.0		0.42

List of human donors (*n* = 69 subjects) included in this study. Paired brains with neuropathological assessments were available for 52 human donors.

Mean ABC scores were determined as follows: A, Aβ plaque score modified from Thal; B, NFT stage modified from Braak; C, neuritic plaque score modified from CERAD.

Group values are presented as mean ± standard deviation. *F* and *p*‐values were determined using one‐way analysis of variance with Tukey's multiple comparisons test. *p*‐values presented in bold type demonstrate significance.

^a^ Severity score.

Abbreviations: Aβ, amyloid beta; AD, Alzheimer's disease; A, Asian; B, Black; CDR, Clinical Dementia Rating; F, female; H, Hispanic; M, male; MCI, mild cognitive impairment; MMSE, Mini‐Mental State Examination; NC, cognitively normal controls; NFTs, neurofibrillary tangles; NTs, neuropil threads; PMI, postmortem interval; W, White.

### 2.1 Early and Profound Retinal Synaptic Degeneration Strongly Links to Cognitive Impairment in AD

To determine the impact of AD on retinal synaptic integrity, we initially conducted a histological analysis of glutamatergic presynaptic (VGLUT1) and postsynaptic (PSD95) proteins in retinal synapse‐rich inner and outer plexiform layers (IPL/OPL), in a cohort of AD and MCI patients compared with age‐ and sex‐matched NC controls (*n* = 51; Figure 1A,B). We further analyzed the presynaptic vesicle glycoprotein synaptophysin and the postsynaptic glutamatergic receptor (NMDAR2A) in a subset of these subjects (*n* = 29–36). Synaptic marker expression was next correlated with disease status in respective brain regions (*n* = 43). Immunohistochemical (IHC) analysis of these pre‐ and postsynaptic markers showed early and progressive synaptic protein loss in MCI and AD retinas (Figure 1C,D; extended data in Figure S1A,B), with distinct distribution patterns for presynapses (IPL and OPL) and postsynapses (OPL dominant) markers. Quantitative IHC analysis revealed highly significant reductions in presynaptic markers (VGLUT1 and synaptophysin) by 50%–57% in MCI retinas and 59%–77% in AD retinas compared to NC controls (*p*PP < 0.0001, Figure 1E). Postsynaptic marker levels (PSD95 and NMDAR2A) followed similar trajectories, with marked decreases of 48%–58% and 66%–72% in MCI and AD retinas, respectively (*p* < 0.0001, Figure 1F). A detailed geometrical analysis of retinal synaptic markers across central (C), mid‐peripheral (M), and far‐peripheral (F) subregions demonstrated that the mid‐ and far‐peripheral ST/IT retina exhibited the most pronounced degeneration in AD, particularly for synaptophysin (Figure S1C–F). Furthermore, retinal synaptic markers remained significantly reduced after adjusting for retinal thinning in MCI and AD patients (Figure S1G). This spatial pattern aligns with prior evidence of peripheral‐dominant retinal amyloidosis and tauopathy distribution [28, 37], suggesting localized synaptic vulnerability to AD pathology. The heightened susceptibility of the mid‐peripheral retina may reflect regional differences in metabolic demand and circuit architecture, and the greater dependence of outer retinal (photoreceptor) synapses on choroidal perfusion, potentially increasing their vulnerability to AD‐related amyloid/tau stressors early in disease.

To test this, we evaluated the relationship between synaptic integrity and Aβ/tau pathology in the same retinal regions (representative images in Figure S1H,I). Among the NC group, a strong correlation was detected between retinal phosphorylated serine‐396 (pS396)‐tau and postsynaptic NMDAR2A protein (*r* = −0.79, P = 0.0037, Figure S1J). Note that all multivariable correlations reported hereafter are presented as adjusted P values (P*_adj._*). Pearson correlation coefficient (*r*_P_) analysis of retinal synaptic markers and retinal amyloidosis and tau isoforms (Table 2) revealed that all pre‐ and postsynaptic proteins, particularly synaptophysin and PSD95, were very closely associated with and highly vulnerable to Aβ_42_ levels in the same regions [*r* = −0.86 to −0.87, P*_adj._* < 0.0001; Figure 1G]. These correlations remained strong and significant even when the analysis was restricted to NC and MCI cases [*r* = −0.76 to −0.82, P*_adj._* < 0.01–0.0001], further supporting that retinal glutamatergic synaptic loss is an early Aβ_42_‐associated event at the MCI stage, before advanced AD dementia. Synaptophysin was also tightly associated with Aβ oligomers in the retina (*r* = −0.83, P*_adj._* < 0.01; Table 2). With respect to retinal tauopathy, synaptophysin was very strongly correlated with and susceptible to pathogenic tau species, particularly pS396‐tau isoforms (*r* = −0.81, P*_adj._* < 0.0001; Figure 1H). In addition, retinal VGLUT1 levels were negatively correlated with Aβ_42_ and pS396‐tau burden [*r* = −0.72 to −0.81, P*_adj._* < 0.0001; Table 2 and Figure 1H]. Most immature retinal tau species, including citrullinated arginine‐209 (CitR209)‐tau, pS396‐tau, and T22^+^ tau oligomers, exhibited strong associations with both pre‐ and postsynaptic markers (Table 2), whereas AT8^+^ p‐tau showed significant correlations only with synaptophysin and PSD95 (*r* = −0.60 to −0.68, P*_adj._* < 0.05–0.01). Of note, mature forms of abnormal tau, including paired helical filament (PHF)‐tau and MC1^+^ neurofibrillary tangles (NFTs), did not correlate with any synaptic proteins in the retina (Table 2). These findings support a model in which retinal synaptic degeneration is driven by local Aβ and tau aggregation, mirroring well‐characterized mechanisms in the AD brain.

**TABLE 2:** Correlations between retinal synaptic markers and retinal AD pathology.

Retinal	Amyloidosis		Tau isoforms					
	Aβ_42_	AβOi	CitR209	T22	pS396	AT8	PHF	MC1
VGLUT1	−0.81****	−0.66**	−0.64**	−0.72**	−0.72****	−0.48	−0.074	−0.18
	38	19	25	21	32	22	10	21
SYP	−0.87****	−0.83**	−0.73**	−0.65*	−0.81****	−0.60*	−0.58	−0.19
	26	13	21	16	21	18	8	16
PSD95	−0.86****	−0.50*	−0.68**	−0.68**	−0.70****	−0.68**	0.018	−0.021
	44	25	25	21	33	23	11	21
NMDAR2A	−0.79****	−0.59*	−0.74***	−0.62*	−0.72***	−0.49	−0.069	−0.14
	32	18	23	20	28	20	10	20

Pearson correlation analyses showing the strength of the association (*r*, upper value), sample size (*n*, lower value), and adjusted P values with Holm‐Šídák multiple‐comparison correction method (adjusted for each retinal synaptic marker against 8 retinal amyloidosis and tauopathy markers): *p  <  0.05, **p  <  0.01, ***p  <  0.001, **** *p*  <  0.0001.

Ultrastructural analysis of the IPL and OPL layers using transmission electron microscopy (TEM), following immunogold labeling with presynaptic (VGLUT1) and postsynaptic (PSD95) markers, further confirmed synaptic morphological disruption and loss of synaptic integrity in the AD retina (Figure 1I). In NC control retinas, synapses displayed well‐preserved architecture (yellow arrows), with intact clefts, membrane‐anchored presynaptic ribbons (red arrows), and presynaptic terminals densely packed with electron‐dense synaptic vesicles (SVs) aligned with clearly defined postsynaptic terminals (Figure 1I, upper panel). In contrast, AD retinas exhibited reduced synaptic density or strength, as well as severe ultrastructural synaptic abnormalities, including darkened and electron‐dense aggregates (agg), fragmented and mislocalized, free‐floating presynaptic ribbons within the cytoplasm, indicating a loss of membrane‐anchored ribbon structures, vesicular leakage, and cellular blebbing (vlb) (Figure 1I, lower panel). Immunostaining of ribbon synapses with Bassoon co‐labeled for VGLUT1 revealed strong colocalization (yellow) marking presynaptic ribbons in the IPL and OPL, which were substantially reduced in AD retinas (Figure 1J; extended images in Figure S1K). These morphological abnormalities are consistent with advanced synaptic degeneration and loss of structural integrity.

Next, we determined whether retinal synaptic loss reflected overall AD severity and stage, as indexed by brain pathology, including Braak stage and ABC scores, and cognitive status as assessed by the clinical dementia rating (CDR) and the mini‐mental state examination (MMSE; Figures 1K,L; extended data in Figure S1L,M, and Tables S2–S5). Stratification by CDR categories revealed progressive reductions in synaptic markers, with VGLUT1 reduced by 36%–47%, synaptophysin by 54%–68%, PSD95 by 48%, and NMDAR2A by 60% in individuals with CDR 1–2 or CDR 3 as compared to individuals with CDR 0 (Figure 1K and Table S2). Stratification by Braak stages revealed similar patterns of significant reductions in retinal VGLUT1 (34%–41%) and SYP (50%–63%) in individuals with Braak stage III–VI compared to stage 0‐II, whereas retinal PSD95 and NMDAR2A were significantly reduced by 52%–54% only in individuals with stage V–VI but not III–IV (Figure S1L and Table S3). Individuals with MMSE scores of ≤26 demonstrated 52%–67% reductions in retinal pre‐ and postsynaptic markers as compared to those with MMSE scores above 26 (Figure S1M and Table S4). Spearman's rank correlation analyses, with multivariable adjustment, revealed significant associations between retinal synaptic markers and brain AD‐related pathology and disease status (Heatmap: Figure 1L and Table S5). While weak to moderate associations were found between retinal synaptic markers and brain Aβ‐plaque or NFT neuropathological burden, a strong association was detected between retinal synaptophysin and brain NFT severity (*r* = −0.65, *p* < 0.01; Table S5). Notably, retinal postsynaptic markers (PSD95, NMDAR2A), compared with presynaptic proteins, showed more significant correlations with disease stage (Braak, ABC), whereas presynaptic markers (VGLUT1, synaptophysin) exhibited slightly stronger associations with cognitive deficits (CDR, MMSE; Figure 1L). Overall, our findings reveal early, robust, and spatially distinct patterns of synaptic density loss in the human AD retina, which were tightly coupled to both retinal and brain pathology. The peripheral retina emerges as a focal point of synaptic vulnerability, likely reflecting early Aβ and tau accumulation.

### 2.2 Proteomic Evidence of Retinal Neurosynaptic Deficits in AD with p75NTR Upregulation

We next sought to delineate synaptic‐ and neurodegeneration‐targeted molecular pathways in the AD retina by analyzing an independent human cohort using an integrated protein biochemistry workflow encompassing WB, secondary MS‐based proteomics, and quantitative ELISA. This cohort comprised neuropathologically confirmed AD patients (*n* = 9; mean age = 82.0 ± 15.7 years; 6 females, 3 males) and age‐ and sex‐matched individuals with normal cognition (NC; *n* = 7; mean age = 76.7 ± 5.4 years; 4 females, 3 males). Initial proteomic findings from this cohort were previously reported (Proteomics Identification Database ‐ PRIDE ID: PXD040225) [28]. Preranked gene set enrichment analysis (GSEA) [104] of AD retinal MS data further confirmed inhibited synapse‐ and vision‐linked pathways, including Kyoto Encyclopedia of Genes and Genomes (KEGG) “adherens junction” and “phototransduction,” alongside Gene Ontology (GO) “response to light stimulus” and “actin filament‐based transport,” while activated GO “negative regulation of cell cycle”—consistent with disrupted retinal connectivity, impaired neuronal homeostasis, and neurodegeneration in AD (Figure S2).

**FIGURE 2:**
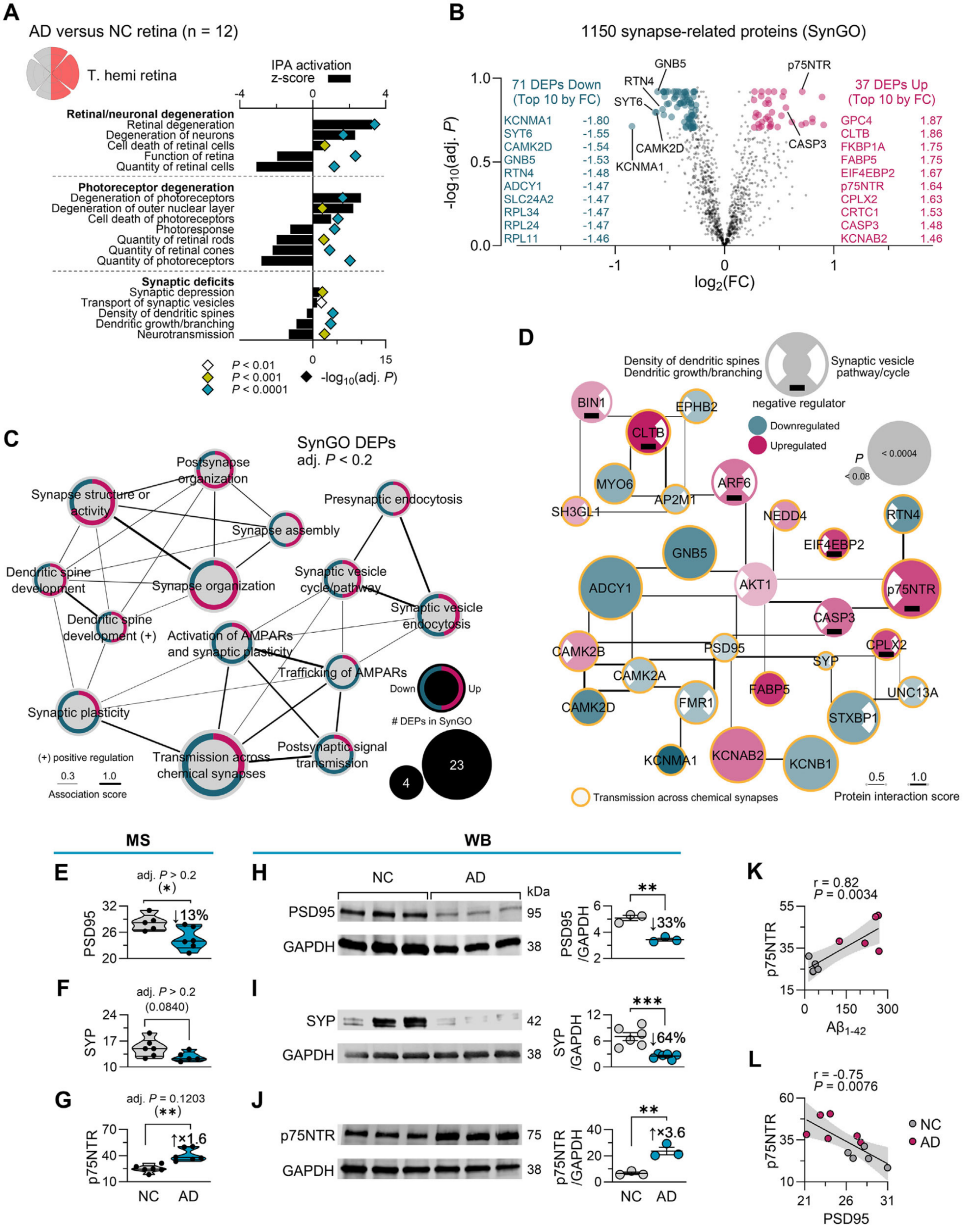
Dysregulated AD retinal proteome associated with synaptic and neuronal loss. (A–D) Pathway analysis of global proteomics via mass spectrometry (MS) in AD (*n* = 6) versus NC control (*n* = 6) retinas. For this analysis, DEPs were defined by |FC| > 1.2 and adj. *p* < 0.2. (A) Dysregulated biological pathways related to retinal/neuronal degeneration, photoreceptor degeneration, and synaptic deficits, revealed by Ingenuity Pathway Analysis (IPA) of DEPs in the AD retina. Bar and symbol graphs represent activation z‐scores and Benjamini‐Hochberg‐adjusted *p*‐values, respectively. The range of *p*‐values is indicated as color‐coded symbols. (B) Volcano plot of dysregulated synapse‐associated proteins in AD versus NC control retinas, extracted from the SynGO release 1.2 knowledge base. The top 10 upregulated and downregulated DEPs are listed with their fold change (FC). (C) Pathway network (Metascape) from Gene Ontology (GO) analysis of synaptic‐relevant DEPs revealed by SynGO. The size of the nodes represents the number of DEPs in SynGO, while the thickness of edges represents the overlapping DEPs (association score) between GO terms. (D) Interaction network analysis of DEPs related to synaptic plasticity, synaptic vesicle cycle, and neurotransmission built from the STRING v12.0 database. (E–G) Violin plots of the postsynaptic marker PSD95 (E; *n* = 5–6/group), the presynaptic protein SYP (F; *n* = 5–6/group), and the neuron‐type‐specific death receptor NGFR/p75NTR (G; *n* = 6/group), quantified by MS, in AD versus control retinas. (H–J) Western blot analysis and normalized quantitation of retinal PSD95 (H; *n* = 3/group), SYP (I; *n* = 6/group) and p75NTR (J; *n* = 3/group). Full blots are shown in Figure S4. (K) Pearson's correlations between retinal Aβ_1‐42_ levels, measured by ELISA, and retinal p75NTR, quantified by MS. (L) Pearson's correlation between retinal PSD95 and retinal p75NTR. Violin plots show individual subjects (circles) with lower, median, and upper quartiles. *p < 0.05, ** *p* < 0.01, *** *p* < 0.001, by two‐sided unpaired *t*‐test (E–J).

Further, Ingenuity Pathway Analysis (IPA) of differentially expressed proteins (DEPs: |fold change| > 1.2 and false discovery rate (FDR)‐adjusted *p*‐values < 0.2) in AD versus NC retinas revealed perturbation of retinal homeostasis and function, with prominent photoreceptor degeneration, alongside synaptic dysfunction spanning synaptic depression, dendritic spine density/architecture, synaptic vesicle trafficking, and neurotransmission (Figure 2A). A substantial fraction of retinal proteins expressed in photoreceptors, including several with exclusive photoreceptor expression, was markedly dysregulated in AD (Figure S3A,B). Within photoreceptor outer segments, the site of phototransduction, opsins including rhodopsin (RHO), long‐wave‐sensitive opsin 1 (OPN1LW), and short‐wave‐sensitive opsin 1 (OPN1SW) were reduced by ∼47%–61% in AD retinas, accompanied by significant decreases in cyclic nucleotide–gated channel subunits (CNGA1, CNGB1), transducin γ subunit (GNGT1), and Na^+^/Ca^2+^–potassium channels (SLC24A1/2), key components of the visual transduction cascade. Photoreceptor inner‐segment proteins implicated in excitability and phototransduction—including the potassium voltage‐gated channels subfamily B member 1 (KCNB1) and modifier subfamily V member 2(KCNV2), and potassium calcium‐activated channel subfamily M alpha 1 (KCNMA1)—were similarly decreased, together with peripherin‐2 (PRPH2), in the AD retina (Figure S3A,B). In parallel, pro‐apoptotic mediators were increased in the AD retina, including Fas‐associated protein with death domain (FADD), pro‐apoptotic caspase 3 (CASP3), and the Aβ‐binding death receptor p75NTR (Figure S3C,D) [95, 105, 106]. Retinal p75NTR levels tightly and inversely correlated with all photoreceptor markers (Figure S3E; P*_adj._* < 0.05), consistent with pronounced photoreceptor degeneration in AD.

Alongside neurodegeneration, synaptic integrity was compromised, as evidenced by downregulation of synaptic proteins enriched in photoreceptor terminals, including synaptotagmin‐6 (SYT6) and retinoschisin‐1 (RS1) (Figure S3A). We analyzed synapse‐associated DEPs in AD retinas using the Synaptic GO (SynGO) 1.2 database (Figure 2B–D). Of the 1602 synapse‐associated proteins included in the database, 1150 were detected in the human retina, with 37 significantly upregulated and 71 significantly downregulated in the AD retina (Figure 2B; list of synapse‐associated DEPs in Tables S6 and S7). These accounted for approximately 14% and 15% of upregulated and downregulated DEPs in the AD retina, respectively (Figure S3F). GO‐guided pathway analysis of the 108 significantly altered proteins, queried across GO Biological Process, Reactome, KEGG, and WikiPathways, converged on synaptic dysregulation in AD retinas, marked by reduced synaptic plasticity, chemical synaptic and postsynaptic signaling, and α‐amino‐3‐hydroxy‐5‐methyl‐4‐isoxazolepropionic acid receptor (AMPAR) activation/trafficking, alongside increased signatures of synaptic organization and structural remodeling (Figure 2C). Consistent with these changes, positive regulators of synaptic transmission were largely downregulated, including the Ca^2^^+^/calmodulin‐regulated adenylyl cyclase 1 (ADCY1/AC1) linked to visual/sensory and photoreceptor biology, the core G‐protein subunit β5 (GNB5) which tunes synaptic signaling and light responses, and the synaptic markers PSD95 and synaptophysin (Figure 2D). Conversely, negative regulators of synaptic vesicle recycling—including synaptic vesicle endocytosis‐related clathrin light chain B (CLTB), bridging integrator 1 (BIN1) and adenosine diphosphate (ADP)‐ribosylation factor 6 (ARF6)—and dendritic spine formation, including the translation initiation repressor eukaryotic translation initiation factor 4E binding protein 2 (EIF4EBP2), CASP3, and p75NTR, were upregulated in the AD retina (Figure 2D). Overall, these synaptic proteome changes indicate that AD‐related retinal pathology extends beyond photoreceptor loss to active remodeling and failure of neuronal connectivity, underscoring synaptic dysfunction as a central feature of retinal neurodegeneration.

WB of retinal homogenates from a subset of AD patients and NC individuals corroborated the MS findings, confirming reduced expression of PSD95 (33% decrease; *p *< 0.01) and synaptophysin (64% decrease; *p* < 0.001), alongside an increase in p75NTR (3.6‐fold; *p* < 0.01) in AD retinas (Figure 2E–J; full extended blots in Figure S4). Given that Aβ_42_ binds p75NTR and can promote neuritic degeneration, synaptic loss, and apoptosis, we next tested the p75NTR association with Aβ_1‐42_ peptide levels (measured by a quantitative sandwich ELISA) and synaptic markers (Figure 2K,L). Retinal p75NTR expression very strongly correlated with retinal Aβ_1–42_ levels (Figure 2K; r = 0.82, P = 0.0034) and was inversely associated with the glutamatergic postsynaptic marker PSD95 (Figure 2L; r = −0.75, P = 0.0076). Together, these relationships support the existence of an Aβ_42_–p75NTR axis contributing to synaptic loss and neurodegeneration in the AD retina.

Overall, these proteomic and biochemical analyses demonstrate that AD retinas undergo profound photoreceptor marker loss and synaptic dysfunction, potentially driven by Aβ_42_ accumulation and p75NTR‐mediated apoptosis.

### 2.3 Oxidative Stress and p75NTR Apoptotic Signaling‐Mediated Synaptic Loss in the AD Retina

**FIGURE 3:**
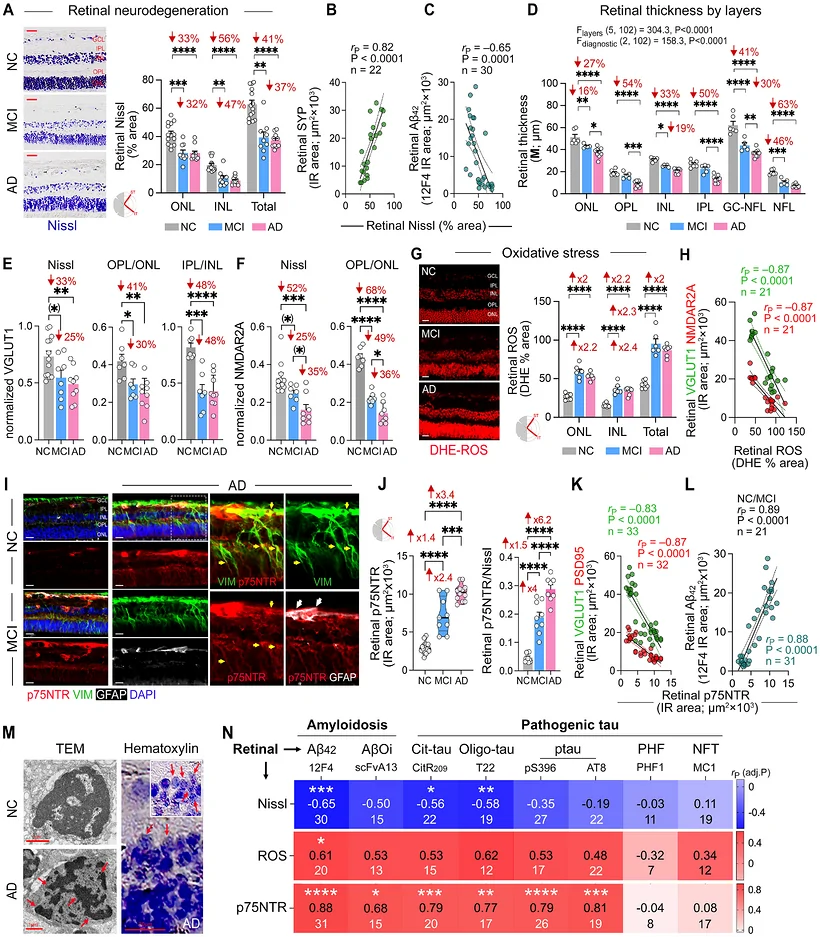
Retinal neurodegeneration and death receptor signaling in MCI and AD patient retinas. (A) Representative Nissl‐stained retinal cross‐sections from individuals with NC, MCI, and AD dementia. Scale bars: 20 µm. Quantification of Nissl % area in the ONL, INL, and total retina (GCL + INL + ONL) in MCI (*n* = 9) and AD patients (*n* = 10) versus NC controls (*n* = 14). (B, C) Pearson's correlations (*r*_P_) between Nissl % area and retinal SYP (B) and Aβ_42_ burden (C). (D) Mid‐peripheral retinal layer thickness (ST/IT regions) in MCI (*n* = 5), AD (*n* = 9) and NC (*n* = 6) individuals. (E) Quantification of VGLUT1: total VGLUT1 normalized to Nissl^+^ neurons; OPL VGLUT1 adjusted to ONL thickness; IPL VGLUT1 adjusted to INL thickness. (F) Quantification of NMDAR2A: total NMDAR2A normalized to Nissl^+^ neurons; OPL NMDAR2A adjusted to ONL thickness. (G) Representative dihydroethidium (DHE) staining of retinal reactive oxygen species (ROS) in NC, MCI, and AD retinas. Scale bars: 20 µm. The bar graph shows quantification of DHE‐ROS in the ONL, INL, and total retina in a subset of NC (*n* = 8), MCI (*n* = 6), and AD (*n* = 7). (H) Pearson's correlation (*r*_P_) between neuronal DHE^+^ % area and retinal VGLUT1 and NMDAR2A. (I) Immunofluorescence of retinal cross‐sections labeled for p75NTR (nerve growth factor receptor; red), vimentin (green), GFAP (white) and nuclei (DAPI, blue) from individuals with NC, MCI and AD dementia. Vimentin^+^ Müller glia expressing p75NTR are shown by yellow arrows, and GFAP^+^ astrocytes colocalizing with p75NTR are shown by white arrows. Scale bars: 20 µm. (J) Quantification of retinal p75NTR IR area and p75NTR adjusted to Nissl^+^ neurons, in MCI (*n* = 9) and AD (*n* = 8–12) patients compared with NC controls (*n* = 11–13). (K, L) Pearson's correlations (*r*_P_) between retinal p75NTR IR area and retinal VGLUT1 and PSD95 IR area (K), as well as 12F4^+^Aβ_42_ IR area across all diagnostic groups and within NC/MCI only (L). (M) TEM and H&E images showing neuronal nuclear morphology in NC and AD retinas. Shrunken, dense, and darkly stained pyknotic nuclei were observed in AD retinal cells (red arrows), indicative of cell death. (N) Heatmap showing Pearson's correlations (*r*_P_) and significance (Holm‐Šídák‐adjusted *p* values for each retinal neuronal marker against 8 retinal amyloidosis and tauopathy markers). Associations were assessed between retinal degeneration markers (Nissl, DHE‐ROS, p75NTR) and retinal AD pathology, including amyloidosis (Aβ_42_ and scFvA13^+^ intraneuronal Aβ oligomers – AβOi) and tauopathy (pS396‐tau, T22‐tau oligomers). The violin plot shows individual subjects (circles) with lower, median, and upper quartiles. Bar graphs display group means ± SEM. *p < 0.05, **p < 0.01, ***p < 0.001, ****p < 0.0001, by two‐sided unpaired *t*‐test (E, F; values in parentheses), or one‐way (E, F, J) or two‐way (A, D, G) ANOVA followed by Tukey's multiple‐comparisons test. Percentage (%) changes are shown in red. NFL, nerve fiber layer; GCL, ganglion cell layer; IPL, inner plexiform layer; INL, inner nuclear layer; OPL, outer plexiform layer; ONL, outer nuclear layer.

Our protein biochemistry analyses show that synaptic protein deficits and activation of death receptor pathways have emerged as central pathological hallmarks of the AD retina. We next interrogated neurodegeneration‐associated processes within anatomically defined retinal subregions and specific cellular layers in an expanded human cohort (Figure 3 and Table 3; extended data in Figure S5). To this end, we initially assessed neuronal integrity in retinas from MCI (*n* = 9), AD (*n* = 10), and age‐matched NC controls (*n* = 14) using neuronal‐specific Nissl staining, which labels nuclei and granules (i.e., ribosomal RNA) in purple (Figure 3A). Quantitative analysis revealed substantial neuronal loss across nuclear cell layers, with highly significant 32%–33% losses of outer nuclear layer (ONL) neurons and 47%–56% losses of inner nuclear layer (INL) neurons in MCI and AD retinas compared with NC controls (*p* < 0.01–0.0001; Figure 3A). Retinal Nissl^+^ neuronal area was very strongly and directly correlated with retinal synaptophysin (*r* = 0.82, *p* < 0.0001; Figure 3B) and strongly and negatively correlated with retinal Aβ_42_ burden (*r* = −0.65, P = 0.0001; Figure 3C), further supporting a link between Aβ_42_ accumulation and both neuronal and synaptic loss in the AD retina. There was also a moderate association between retinal Nissl and retinal CitR209‐tau (*r* = 0.56, P = 0.05), but not with other tau isoforms (Table 3). To further characterize retinal neurodegeneration, we performed histomorphometric analysis of retinal thickness across six layers (ONL, OPL, INL, IPL, ganglion cell layer/nerve fiber layer, GC/NFL, and NFL) in predefined C/M/F anatomical subregions (Figure 3D, mid periphery; extended data across all retinal layers and subregions are detailed in Figure S5A–I). The most noticeable thinning was detected in the mid‐peripheral retina (Figure 3D), prominently in axonal‐ and synapse‐rich layers (NFL, IPL, OPL), which were reduced by 50%–63% in AD versus NC (*p* < 0.0001). The earliest and most pronounced loss in MCI retinas occurred in the GCL/NFL, diminished by 30%–46% (*p* < 0.001–0.0001). After adjusting synaptic markers to Nissl^+^ neurons and correcting OPL and IPL synaptic measures for thinning of the corresponding ONL and INL, respectively, we detected significant synaptic protein loss already at the MCI stage, with further decline in AD, independent of neurodegeneration, most notably for VGLUT1 and NMDAR2A (Figure 3E,F and Figure S5J,K).

**TABLE 3:** Correlations between retinal neurodegeneration‐related and UCHL1 markers with retinal AD pathology.

	Synaptic markers				Amyloidosis		Tau isoforms					
Retinal	VGLUT1	SYP	PSD95	NMDAR2A	Aβ_42_	AβOi	CitR209	T22	pS396	AT8	PHF	MC1
Nissl	0.68*** 31	0.82**** 22	0.69*** 32	0.79**** 26	−0.65*** 30	−0.50 15	−0.56* 22	−0.58 19	−0.35 27	−0.19 22	−0.03 11	0.11 19
ROS (DHE)	−0.87****	−0.83***	−0.88****	−0.87****	0.61*	0.53	0.53	0.62	0.53	0.48	−0.32	0.34
	21	16	21	21	20	13	15	12	17	12	7	12
p75NTR	−0.83****	−0.84****	−0.87****	0.81****	0.88****	0.68*	0.79***	0.77**	0.79****	0.81***	−0.039	0.076
	33	22	32	27	31	15	20	17	26	19	8	17
UCHL1	0.88****	0.89****	0.91****	0.86****	−0.88****	−0.57**	−0.73***	−0.65**	−0.70****	−0.63**	−0.21	−0.13
	40	27	46	34	45	25	25	23	34	22	10	23

Pearson correlation analyses showing the strength of the association (*r*, upper value), sample size (*n*, lower value), and adjusted P values with Holm‐Šídák multiple‐comparison correction method (adjusted for each retinal neuronal marker against 12 retinal synaptic, amyloidosis, and tauopathy markers): *p  <  0.05, **p  <  0.01, ***p  <  0.001, ****p  <  0.0001.

Pathological Aβ and tau species have been shown to drive oxidative stress [107, 108, 109]. Given the retina's high metabolic demand, vulnerability to oxidative damage, and progressive neurodegeneration in AD, we next quantified retinal labeling related to reactive oxygen species (ROS) in the MCI and AD retina (Figure 3G and Figure S6A). Dihydroethidium (DHE) labeling [110] revealed a 2.0–2.4‐fold increase in oxidized DHE fluorescence (% area), consistent with elevated superoxide/ROS levels, in the retinal nuclear layers of MCI and AD patients compared with NC controls (*p* < 0.0001). Retinal DHE levels exhibited very strong inverse correlations with retinal synaptic markers (Figure 3H and Figure S6B,C; Table 3; *r* = −0.83 to −0.88, P*_adj._* < 0.0001), alongside strong positive correlations with 12F4^+^ Aβ_42_ area (*r* = 0.61, *p* = 0.0042; Figure S6D), but not with pathogenic tau species. These findings implicate oxidative stress as a downstream mediator of Aβ_42_ accumulation driving synaptic and neuronal loss in the AD retina.

Previously, p75NTR, an Aβ‐responsive death receptor, has been implicated in regulating oxidative stress, apoptosis, and synaptic plasticity, as well as in mediating Aβ‐driven neurite degeneration, tau hyperphosphorylation, and neuronal loss in AD models [91, 92, 93, 94]. Here, it also emerged as a top upregulated synapse‐associated protein in the AD retina from our MS and WB analyses (Figure 2G,J,L). Consistent with these findings, IHC revealed increased p75NTR expression in MCI and AD retinas versus NC. Signal localized primarily to macroglia, colocalizing with GFAP^+^ astrocytes (white arrows) and vimentin^+^ Müller glia (yellow arrows) (Figure 3I; extended images in Figure S6E). Neuronal, non‐glial p75NTR expression was also observed in MCI and AD retinas. Quantification confirmed a robust increase in retinal p75NTR: immunoreactive area rose 2.4–3.4‐fold in MCI and AD versus NC, and furthermore, was elevated 4.0–6.2‐fold when normalized to Nissl^+^ neurons (*p* < 0.0001; Figure 3J). Moreover, retinal p75NTR expression exhibited very strong inverse correlations with all retinal synaptic markers (Table 3), including the pre‐ and postsynaptic markers VGLUT1 and PSD95 (*r* = −0.83 and −0.87, respectively, *p* < 0.0001; Figure 3K), and a very tight direct correlation with retinal Aβ_42_ burden (*r* = 0.88, *p* < 0.0001; NC/MCI: *r* = 0.89, *p* < 0.0001, Figure 3L), supporting its role as a mediator of Aβ‐induced synaptic loss in the retina, mirroring mechanisms observed in the AD brain [93, 111, 112, 113, 114]. Interestingly, strong associations between retinal p75NTR and immature but not mature tau isoforms were also observed (*r* = 0.77–0.81, P*_adj._* = 0.0012–0.00001, Table 3), proposing the involvement of tauopathy in p75NTR‐mediated synaptic degeneration in the AD retina. We next assessed the clinical relevance of retinal p75NTR by evaluating its potential associations with brain AD pathology and cognitive status (Table 4). Retinal p75NTR expression moderately to strongly correlated with brain atrophy, Braak stage, and ABC scores (*r* = 0.53–0.61, P*_adj._* = 0.024–0.0061; Figure S6F and Table 4) and was inversely associated with MMSE scores (*r* = −0.62, P = 0.0005; Figure S6F), indicating that retinal p75NTR upregulation reflects both disease severity and cognitive impairment.

**TABLE 4:** Correlations between retinal UCHL1 and neurodegeneration‐related markers with brain pathology and cognition.

Brain/retina	Aβ plaques	NFTs	Atrophy	Braak	ABC	CDR	MMSE
Nissl	−0.39 25	−0.44 25	−0.005 25	−0.30 25	−0.35 25	−0.64** 25	0.64** 27
ROS (DHE)	−0.081 14	−0.077 14	−0.046 14	−0.13 14	0.28 14	0.33 14	−0.56 17
p75NTR	0.22 26	0.49* 26	0.54* 26	0.61** 26	0.53* 26	0.41 25	−0.62** 27
UCHL1	−0.57*** 42	−0.65**** 42	−0.55*** 42	−0.75**** 42	−0.73**** 42	−0.49** 35	0.81**** 45

Spearman's rank correlation analyses showing the strength of the association (*r*, upper value), sample size (*n*, lower value), and adjusted P values with Holm‐Šídák multiple‐comparison correction method (adjusted for each retinal neuronal marker against 7 brain pathology and cognitive scores): *p  <  0.05, **p  <  0.01, ***p  <  0.001, ****p  <  0.0001.

TEM and hematoxylin and eosin (H&E) staining further showed the ultrastructural features of retinal neuronal death, revealing shrunken, condensed, and darkly stained pyknotic nuclei, hallmarks of oxidative stress‐induced apoptosis or necroptosis, in AD compared with NC donors (Figure 3M, red arrows). A heatmap summary analysis (Figure 3N) highlights strong inter‐relationships among retinal Aβ/tau pathologies and neurodegeneration, with retinal p75NTR emerging as a robustly correlated marker. These correlations reinforce p75NTR as a central integrator of Aβ‐ and tau‐driven toxicity and a potential mediator of synaptic degeneration in the retina, paralleling AD mechanisms in the brain [93, 111, 112, 113, 114].

### 2.4 Early Retinal UCHL1 Dysregulation Links to Proteinopathy and Synaptic Degeneration in AD

**FIGURE 4:**
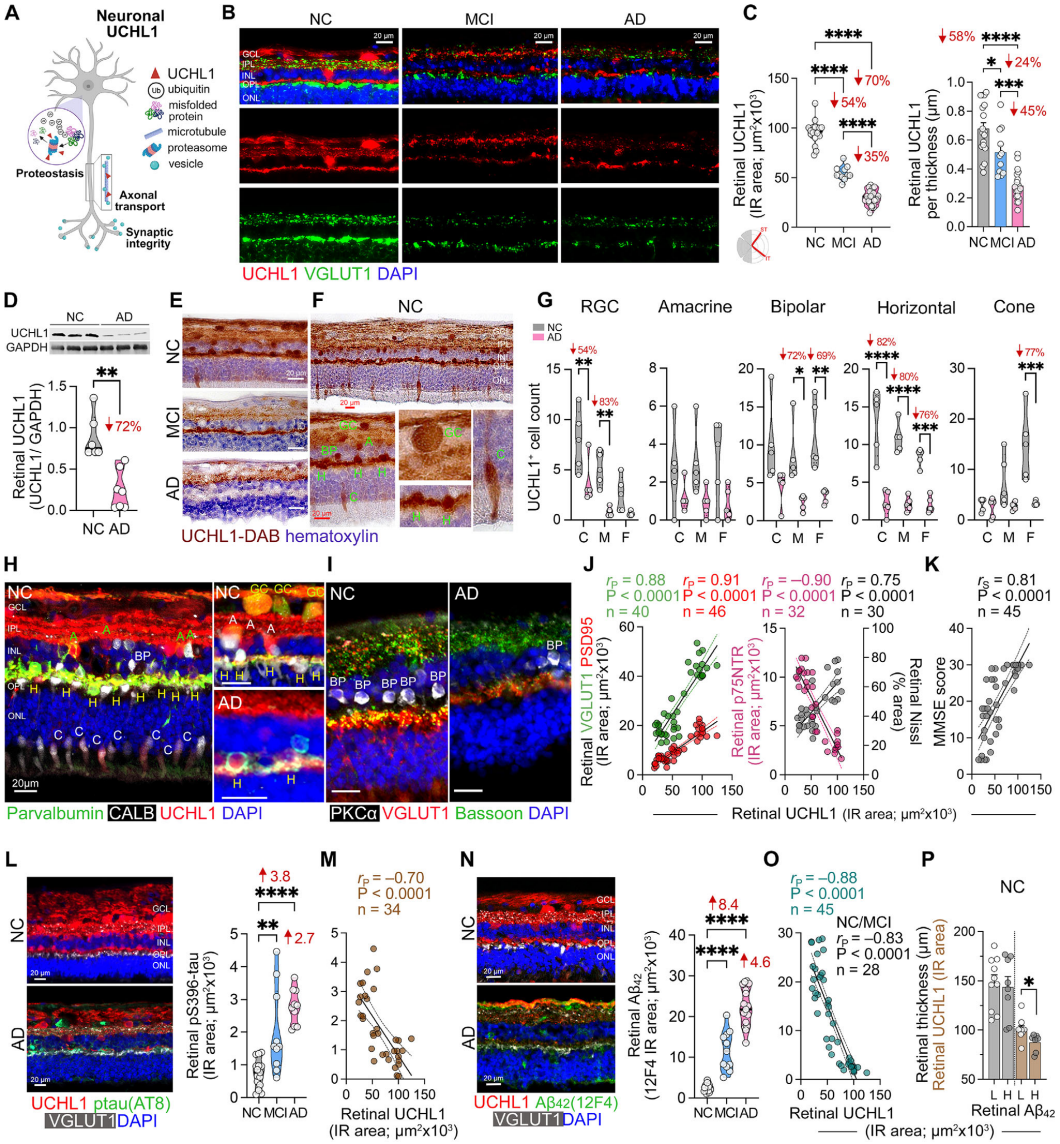
Retinal UCHL1 in relation to synapses and proteinopathies. (A) Schematic illustration of UCHL1 functions in neurons. (B) Fluorescence images of retinal cross‐sections immunolabeled for UCHL1 (red), the presynaptic marker VGLUT1 (green), and nuclei (DAPI, blue). Scale bars: 20 µm. (C) Quantification of retinal UCHL1 IR area in patients with AD (*n* = 21) and MCI (*n* = 11) as compared with NC controls (*n* = 18). (D) Western blot and densitometric analyses of UCHL1 in retinal lysates from AD patients (*n* = 7) versus NC (*n* = 6). The same GAPDH loading control applies to both UCHL1 and SYP (Figure 2I), which were co‐detected on the same membrane, followed by membrane stripping and reprobing for GAPDH (full blots in Figure S7E). (E, F) Representative retinal cross‐section images from MCI and AD patients compared to NC controls immunolabeled with UCHL1 (brown) using peroxidase‐based DAB and hematoxylin counterstaining. Magnified representative images from an NC individual indicate specific cell types (green labels) based on their localization within retinal layers (GC, ganglion cell; A, amacrine; BP, bipolar; H, horizontal; C, cone). Scale bars: 20 µm. (G) Count of UCHL1^+^ cells within retinal ganglion cells (RGCs), amacrine, bipolar, horizontal, and cone cell populations in NC (*n* = 5) and AD (*n* = 5) donors. (H, I) Fluorescence images of retinal cross‐sections immunolabeled for parvalbumin or Bassoon (green), Calbindin (CALB) or PKCα (white), UCHL1 or VGLUT1 (red), and nuclei (DAPI, blue). Scale bars: 20 µm. (J, K) Pearson's or Spearman's correlations between retinal UCHL1 IR area and retinal VGLUT1, PSD95, p75NTR, or Nissl (J), and the MMSE cognitive scores (K). (L) Fluorescence images of retinal cross‐sections immunostained for UCHL1 (red), AT8^+^‐ptau (green), VGLUT1 (white), and nuclei (DAPI, blue). Scale bars: 20 µm. The violin plot presents quantitative analysis of retinal pS396 IR area across subjects in this cohort with AD (*n* = 10), MCI (*n* = 10) and NC (*n* = 15). (M) Pearson's correlation between retinal UCHL1 and retinal pS396‐tau IR areas. (N) Fluorescence images immunolabeled for UCHL1 (red), 12F4^+^ Aβ_42_ (green), VGLUT1 (white), and nuclei (DAPI, blue). Scale bars: 20 µm. The violin plot presents quantitative analysis of retinal 12F4^+^Aβ_42_ IR area across subjects in this cohort with AD (*n* = 17), MCI (*n* = 11) and NC (*n* = 19). (O) Pearson's correlation between retinal UCHL1 IR area and retinal Aβ_42_ burden. (P) Stratification of retinal thickness (µm; *n* ^L^ = 10, *n* ^H^ = 8) and UCHL1 IR area (*n* ^L^ = 10, *n* ^H^ = 7) in NC individuals by high [H] and low [L] retinal Aβ_42_ burden (cutoff = 2.59). Violin plots show individual subjects (circles) with lower, median, and upper quartiles. Bar graphs display group means ± SEM. *p < 0.05, **p < 0.01, ***p < 0.001, ****p < 0.0001, by two‐sided unpaired *t*‐test (D, P), one‐way ANOVA followed by Tukey's post hoc multiple comparison test (C, L, N), or two‐way ANOVA followed by Šidák's post hoc multiple comparison test (G). Percentage (%) changes are shown in red. GCL, ganglion cell layer; IPL, inner plexiform layer; INL, inner nuclear layer; OPL, outer plexiform layer; ONL, outer nuclear layer. The schematic illustration was created using BioRender.com.

UCHL1 is a neuron‐enriched deubiquitinase in the UPS that supports proteostasis, oxidative stress responses, synaptic integrity, and neuronal survival (Figure 4A illustration) [71, 72, 115, 116]. In the brain, UCHL1 facilitates clearance of misfolded proteins and limits the aggregation of neurotoxic species such as Aβ_42_ and p‐Tau [117], whereas reduced UCHL1 expression has been linked to Aβ and p‐tau‐mediated synaptic dysfunction and cognitive decline in AD [73, 79]. We therefore evaluated UCHL1 expression levels and distribution across retinal subregions, layers, and cell types in postmortem retinas from a cohort of AD (*n* = 21, mean age 86.2 ± 8.9 years, 13 females/8 males) and MCI (*n* = 11, mean age 89.3 ± 5.2 years, 7 females/4 males) patients, compared with NC controls (*n* = 18, mean age 82.6 ± 10.3 years, 8 females/10 males) (Figure 4B–H; extended data in Figure S7A–I). We found that in the NC retina, UCHL1 is strongly expressed in multiple layers, with robust signal in synapse‐rich and axonal layers (OPL, IPL, NFL) and reduced signal in the ONL and INL; it lies in close spatial proximity to the glutamatergic presynaptic protein VGLUT1 within the OPL and IPL, with limited direct colocalization. In MCI and AD retinas, UCHL1 is markedly reduced across these cells and layers, mirroring a concomitant decline in VGLUT1 (Figure 4B).

Quantitative analysis showed a significant 54%–70% reduction in UCHL1 immunoreactive area in MCI and AD retinas versus NC (*p* < 0.0001; Figure 4C, left). Extended mapping confirmed 41–80% decreases across C/M/F subregions and the NFL, IPL, and OPL layers, with the largest UCHL1 loss in the AD retina occurring in the OPL (Figure S7B–D). To determine whether reduced retinal UCHL1 simply reflects retinal thinning or neurodegeneration, which is significant in MCI and AD, we normalized UCHL1 to retinal thickness. The reduction persisted: UCHL1 remained significantly decreased by 24% in MCI and 58% in AD retinas (Figure 4C, right, *p* < 0.05‐0.0001; extended data of UCHL1 normalized to inner retinal thickness in Figure S7D). In agreement with these results, WB analysis in an additional human cohort demonstrated a 72% decrease in UCHL1 expression normalized to GAPDH in 7 AD relative to 6 NC retinas (Figure 4D; full blots are shown in Figure S7E). Proteomics showed broad UPS dysregulation in AD retinas: GSEA indicated suppression of the GO “protein SUMOylation” program alongside activation of the WikiPathway “Parkin–ubiquitin–proteasomal system pathway,” consistent with stress‐driven proteostasis and UCHL1‐linked post‐translational modification (Figure S8A,B). In line with prior work implicating UPS dysfunction in AD [83], MS further revealed increased particulate/aggregated and oxidized UCHL1 and UCHL3 (Figure S8C,D), whereas our IHC and WB analyses showed reduced soluble UCHL1. Heatmap analysis and Metascape network mapping demonstrated coordinated dysregulation of deubiquitination/UPS proteins and pathways in AD retinas (Figure S8E,F). These analyses revealed interconnected disruptions in the UPS, autophagy, axonal transport, and axonogenesis, highlighting retinal UCHL1 as a central hub linking proteostasis failure to p75NTR‐mediated synaptic loss and neurodegeneration. Together, these findings indicate a marked retinal UCHL1 imbalance in AD, paralleling reports in the AD brain [118, 119, 120, 121, 122].

To assess cell‐type vulnerability to UCHL1 loss, we analyzed UCHL1^+^ cells by morphology, size, and laminar position using 3,3'‐diaminobenzidine (DAB)/hematoxylin IHC (Figure 4E,F; Figure S7F,G) and by immunofluorescence with cell‐specific markers (Figure 4H,I; Figure S7H,I). In a subset cohort, AD retinas showed 54%–83% reductions in UCHL1 across multiple cell types versus NC, with the most consistent declines in horizontal and bipolar cells; reductions in amacrine cells, far‐peripheral ganglion cells, and central/mid‐peripheral cones did not reach statistical significance (Figure 4G). In NC retinas, UCHL1 colocalized predominantly with parvalbumin (a marker of horizontal and ganglion cells [123, 124]) and also with calbindin (a marker of amacrine, horizontal, ganglion, and bipolar cell subsets, and cones [125]), whereas these signals were markedly diminished in AD retinas (Figure 4H and Figure S7H). Immunolabeling for protein kinase C‐α (PKCα), a rod bipolar cell marker [126], together with presynaptic VGLUT1 and ribbon‐synapse Bassoon, supported bipolar cell loss in AD (Figure 4I and Figure S7I). Together, these data imply that UCHL1 loss disproportionately affects horizontal and bipolar cells and aligns with synaptic compromise in AD.

To determine the potential clinical significance of retinal UCHL1 in AD, we assessed its relationships with various retinal markers, brain AD‐related pathology, disease stage, and cognitive parameters using correlation analyses adjusted for multiple variables (Figure 4J–P and Tables 3, 4). Retinal UCHL1 exhibited robust correlations with all synaptic markers, particularly the presynaptic VGLUT1 and the postsynaptic PSD95 expression (*r* = 0.88–0.91, P*_adj._* < 0.0001; Figure 4J and Table 3). We also identified very strong to strong inverse associations between retinal UCHL1 and the cell death receptor p75NTR (*r* = −0.90, *p* < 0.0001; Figure 4J), retinal neurodegeneration as measured by Nissl‐positive % area (*r* = 0.75, *p* < 0.0001; Figure 4J), and retinal DHE/ROS levels (*r* = −0.77, *p* < 0.0001; not shown). Moreover, retinal UCHL1 displayed a strong positive correlation with MMSE scores (*r* = 0.81, P*_adj._* < 0.0001; Figure 4K and Table 4), suggesting a direct link between retinal UCHL1 loss and cognitive decline. Notably, marked reductions in UCHL1 in MCI and AD retinas coincided with elevated pathogenic tau and Aβ species (Figure 4L–P, Table 3). Except for retinal mature PHF‐tau and MC1^+^ tau, UCHL1 showed moderate to very strong inverse correlations with various abnormal tau and Aβ species (Table 3), most prominently pS396‐tau (*r* = −0.70, P*_adj._* < 0.0001), CitR209‐tau (*r* = −0.73, P*_adj._* < 0.001), and Aβ_42_ burden (*r* = −0.88, P*_adj._* < 0.0001; NC/MCI: *r* = −0.83, *p* < 0.0001; Figure 4O). Among NC individuals, those with high retinal Aβ_42_ burden already exhibited reduced UCHL1 despite preserved retinal thickness, compared with those with low Aβ_42_ (*p* < 0.05; Figure 4P), suggesting that retinal UCHL1 deficiency arises early in disease progression, before tissue atrophy, and may emerge during the preclinical phase.

### 2.5 Fibrillar Aβ_42_ Triggers Early UCHL1 and Synaptic Deficits Prior to Overt Neurodegeneration in Neuronal Cultures

**FIGURE 5:**
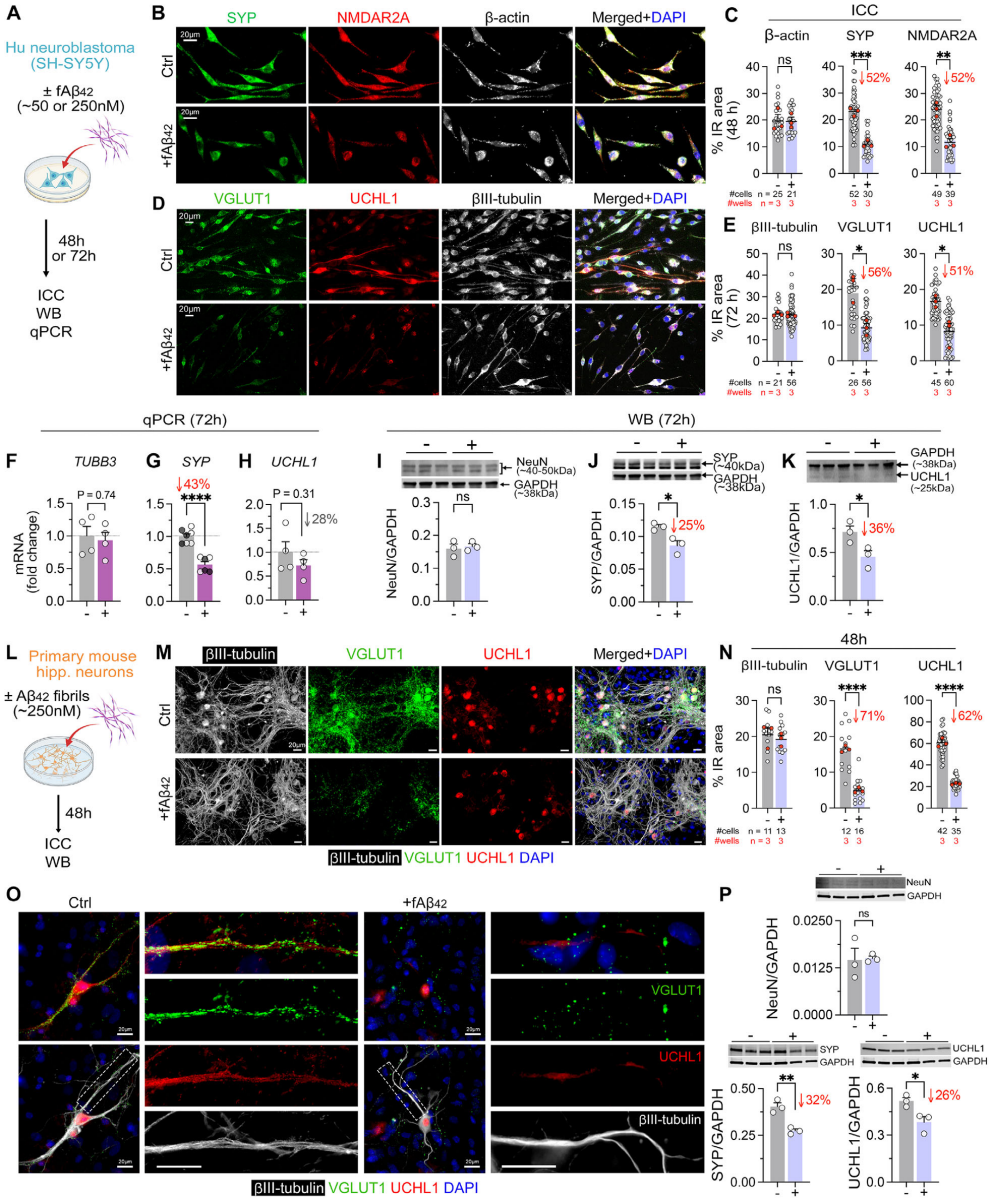
Aβ_42_ fibrils trigger UCHL1 and synaptic deficits before overt neurodegeneration in human and primary mouse neuronal cultures. (A) Experimental in vitro design: retinoic acid (RA) differentiated SH‐SY5Y human (Hu) neurons were treated with fibrillar (f)Aβ_42_ (50 or 250 nM) or left untreated (Ctrl) for 48 h or 72 h. (B) Fluorescence micrographs of SH‐SY5Y neurons ± 250 nM fAβ_42_ for 48 h (2 × 10^4^ cells/well, *n* = 3/group), immunolabeled for either synaptophysin (SYP; green), NMDAR2A (red), β‐actin (white), and nuclei (DAPI, blue). (C) Quantitative ICC analysis of β‐actin, synaptophysin (SYP), and NMDAR2A % IR area in neuronal cells treated with vehicle or 250 nM fAβ_42_ for 48 h. White circles indicate individual cells, illustrating cell‐to‐cell variability; red circles indicate the mean of all analyzed cells within each biological replicate (culture well). Statistical analyses and % changes were based on these biological replicate means and are presented as mean ± SEM (*n* = 3 wells/group). (D) Fluorescence micrographs of SH‐SY5Y neurons ± 250 nM fAβ_42_ for 72 h (2×10^4^ cells/well, *n* = 3/group), immunolabeled for VGLUT1 (green), UCHL1 (red), βIII‐tubulin (white), and nuclei (DAPI, blue). Scale bars: 20 µm. (E) Quantitative ICC analyses of βIII‐tubulin, VGLUT1, and UCHL1% IR area per neuronal cell treated with vehicle or 250 nM fAβ_42_ for 72 h. White circles indicate individual cells; red circles indicate the mean of all analyzed cells within each biological replicate (culture well). Statistical analyses and % changes were based on these biological replicate means and are presented as mean ± SEM (*n* = 3 wells/group). (F–H) Real‐time PCR analyses of *TUBB3*, *SYP*, and *UCHL1* mRNA fold changes normalized to *GAPDH* mRNA in SH‐SY5Y neurons ± 250 nM fAβ_42_ for 72 h [4×10^5^ cells per 60‐mm dish, *n* = 3–4/group; *SYP* plot: differentiated (white circles) and undifferentiated (black circles) SH‐SY5Y neurons treated with or without 250‐ and 50 nM fAβ_42_, respectively, for 72 h]. (I–K) Western blot densitometric quantitation of NeuN (I), synaptophysin (J), and UCHL1 (K) proteins normalized to GAPDH from neuronal cell pellets ± 250 nM fAβ_42_ for 72 h (4×10^5^ cells per 60‐mm dish, *n* = 3/group). Same GAPDH loading control applies to both NeuN and SYP (I and J panels), which were co‐detected on the same membrane, followed by membrane stripping and reprobing for GAPDH (full blots in Figure S9J). (L) Experimental in vitro design: primary mouse hippocampal neurons (mature; 2 × 10^5^ cells per well, DIV14) were exposed to 250 nM fAβ_42_ for 48 h. (M) Fluorescence micrographs of primary mouse hippocampal neurons ± fAβ_42_ immunolabeled with βIII‐tubulin (white), VGLUT1 (green), UCHL1 (red), and nuclei (DAPI, blue). (N) Quantitative ICC analyses of βIII‐tubulin, VGLUT1, and UCHL1% IR area per neuronal cell treated with vehicle or 250 nM fAβ_42_ for 48 h. White circles indicate individual cells; red circles indicate the mean of all analyzed cells within each biological replicate (culture well). Statistical analyses and % changes were based on these biological replicate means and are presented as mean ± SEM (*n* = 3 wells/group). (O) High‐magnification micrographs of primary mouse hippocampal neurons ± fAβ_42_ and immunolabeled for VGLUT1 (green), UCHL1 (red), βIII‐tubulin (white), and nuclei (DAPI, blue). Straightened dendritic segments from the selected white dashed boxes reveal close apposition of UCHL1 and VGLUT1 along dendrites, with occasional colocalized puncta (yellow), in control neurons; both signals are markedly diminished after fAβ_42_ exposure. Scale bars: 20 µm. (P) Western blot densitometric quantitation of NeuN, synaptophysin, and UCHL1 proteins normalized to GAPDH from neuronal cell pellets ± 250 nM fAβ_42_ for 48 h (2.5 × 10^5^ cells/well, *n* = 3/group). Same GAPDH loading control applies to UCHL1, NeuN, and SYP; UCHL1 was detected first, followed by membrane stripping and sequential reprobing for NeuN and SYP, then stripped again and reprobed for GAPDH (full blots in Figure S10C). Individual data points (circles) as well as group means ± SEM are shown. *p <0.05, **p <0.01, ***p < 0.001, ****p < 0.0001, by two‐sided unpaired *t*‐test (C, E, F–K, N, P). Percentage (%) changes are shown in red. Schematic illustrations A and L were created using BioRender.com.

To determine whether Aβ pathology directly drives UCHL1 deficiency and synaptic dysfunction before neuronal degeneration, we used two complementary neuronal culture systems: differentiated human SH‐SY5Y neurons and mature primary mouse hippocampal neurons (Figure 5; extended data in Figure S9A–J). In SH‐SY5Y neurons, treatment with fibrillar (f)Aβ_42_ for 48 h significantly reduced both synaptophysin and NMDAR2A immunoreactivity by 52%, whereas β‐actin levels were unchanged, indicating early synaptic impairment in the absence of overt structural loss (Figure 5A–C). After 72 h exposure, VGLUT1 and UCHL1 immunoreactivity were reduced by 56% and 51%, respectively, while βIII‐tubulin remained unchanged (Figure 5D,E). Consistent with these protein‐level changes, *SYP* mRNA expression was reduced by 43% following fAβ_42_ exposure, *UCHL1* transcript showed a non‐significant trend of 28% reduction, whereas tubulin beta 3 class III (*TUBB3)* transcript was unaltered (Figure 5F–H). WB analyses further demonstrated significant reductions in synaptophysin and UCHL1 protein levels (25% and 36%, respectively) after 72 h treatment, while neuronal nuclei (NeuN) levels remained unchanged (Figure 5I–K). Together, these findings indicate that Aβ_42_‐induced synaptic and UCHL1 deficits occur before overt neuronal degeneration.

To validate these observations in a second neuronal system, mature primary mouse hippocampal neurons (14 days in vitro ‐ DIV14) were exposed to fAβ_42_ for 48 h (Figure 5L–P; extended data in Figure S10A–C). fAβ_42_ treatment caused marked reductions in VGLUT1 and UCHL1 immunoreactivity (71% and 62%, respectively), whereas βIII‐tubulin remained unchanged (Figure 5M,N). High‐resolution dendritic imaging revealed close juxtaposition of UCHL1 and VGLUT1 puncta, with occasional overlap, along dendrites of control neurons; both signals were markedly diminished following fAβ_42_ exposure (Figure 5O and Figure S10A,B). Consistent with the immunocytochemical findings, WB analyses demonstrated significant reductions in synaptophysin and UCHL1 protein levels (32% and 26%, respectively), whereas NeuN remained unchanged (Figure 5P).

Collectively, these results indicate that fibrillar Aβ_42_ directly induces early UCHL1 depletion and glutamatergic synaptic dysfunction in both human and mouse neurons, preceding overt neuronal degeneration.

### 2.6 Retinal Neuroinflammation Closely Associates with Synaptic Degeneration and UCHL1 Dysregulation

**FIGURE 6:**
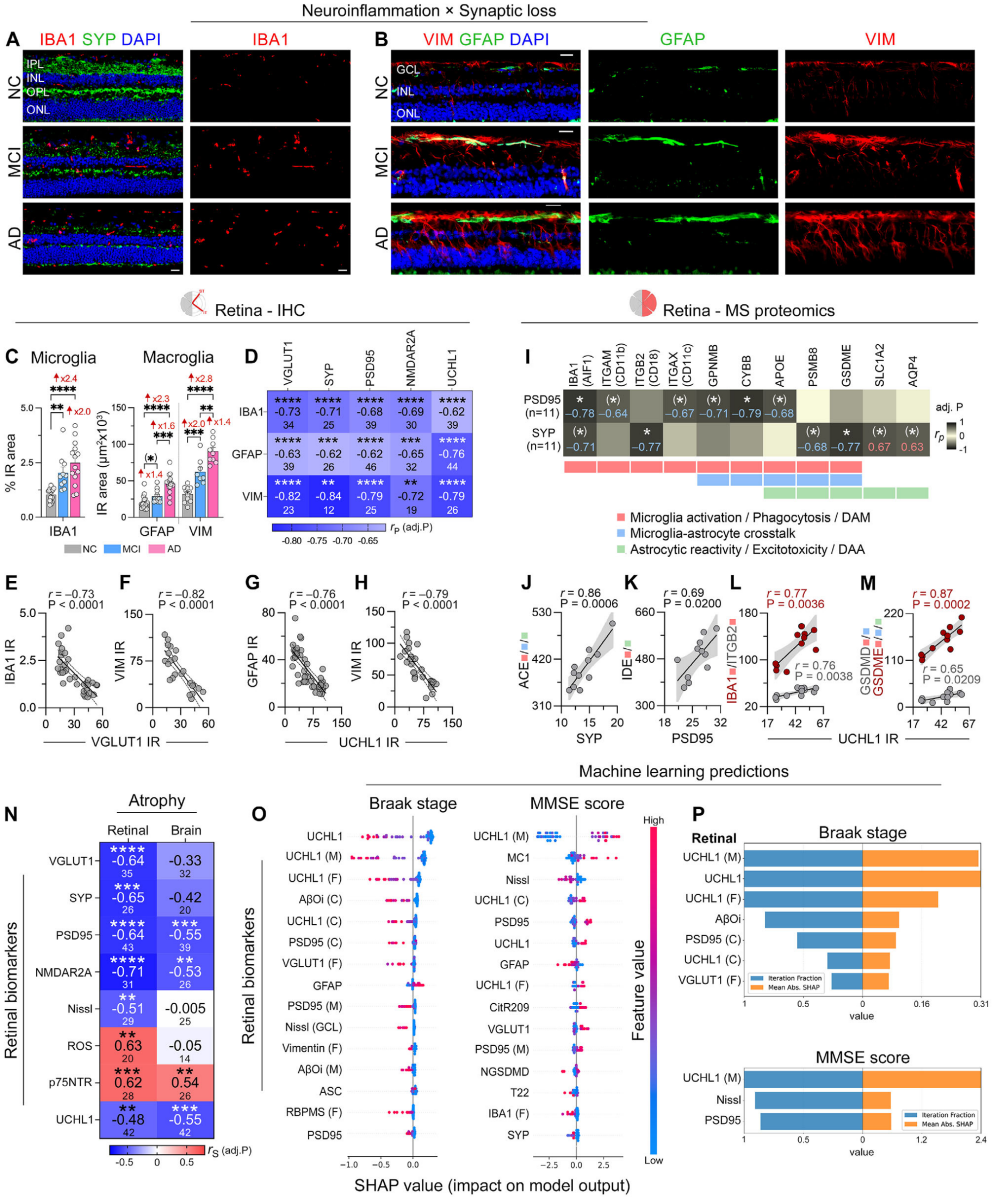
Retinal UCHL1 and synaptic alterations link to neuroinflammation and neurodegeneration and enable machine learning prediction of AD severity. (A) Representative retinal immunofluorescence images from NC, MCI, and AD donors showing presynaptic synaptophysin (SYP, green), microglia (IBA1, red), and nuclei (DAPI, blue), illustrating progressive microglial activation at sites of synaptic loss. Scale bar: 20 µm. (B) Representative retinal immunofluorescence images from NC, MCI, and AD donors stained for the macroglial markers GFAP (green) and vimentin (VIM, red), with DAPI (blue) labeling nuclei, demonstrating progressive macroglial reactivity. Scale bar: 20 µm. (C) Quantification of retinal microglial (IBA1) % IR area and macroglial (GFAP, vimentin) IR area (µm^2^ × 10^3^) in NC (*n* = 11–20) controls, MCI (*n* = 8–11), and AD (*n* = 9–17) subjects. Data from individual subjects (circles) as well as group means ± SEM are shown. Statistical significance (*p < 0.05, **p < 0.01, ***p < 0.001, ****P < 0.0001) determined by one‐way ANOVA followed by Tukey's post hoc multiple comparison test or two‐sided unpaired *t*‐test (asterisk in parentheses). Fold changes are shown in red. (D) Heatmap showing Pearson correlation coefficients (*r_P_*) and significance (Holm‐Šídák‐adjusted P values) between retinal glial activation markers (IBA1, GFAP, vimentin [VIM]) and retinal synaptic proteins (VGLUT1, synaptophysin [SYP], PSD95, NMDAR2A) and UCHL1. (E–H) Representative scatterplots illustrating inverse relationships between retinal inflammatory markers and neuronal/synaptic proteins: IBA1 or vimentin versus VGLUT1 (E, F), GFAP or vimentin versus UCHL1 (G, H). (I) Correlation matrix derived from retinal MS proteomics showing associations between synaptic proteins (PSD95 and SYP) and inflammatory proteins linked to microglial activation, phagocytosis, oxidative stress, and disease‐associated microglia (DAM) (IBA1, ITGAM, ITGB2, IGAX, GPNMB, CYBB, APOE, PSMB8), microglia‐astrocyte crosstalk (GPNMB, CYBB, APOE, PSMB8, GSDME), and astrocytic reactivity, excitotoxicity, and disease‐associated astrocytes (DAA) (APOE, PSMB8, GSDME, SLC1A2, AQP4). Significant correlations are indicated with Holm‐Šídák adjusted *p* values for multiple comparisons (asterisks in parentheses are unadjusted *p* values) and Pearson correlation coefficients. (J–M) Representative proteomic pairwise correlations with unadjusted *p* values between inflammatory and synaptic markers: ACE versus synaptophysin (J), IDE versus PSD95 (K), IBA1 or ITGB2 versus UCHL1 (L), and GSDMD or GSDME versus UCHL1 (M). (N) Heatmap showing Spearman's rank correlation (*r_S_*) and significance (Holm‐Šídák‐adjusted P values) between retinal and brain atrophy and retinal synaptic, neurodegeneration, and UCHL1 markers. (O, P) Identification and robustness of retinal histopathological biomarkers driving Random Forest prediction of Braak stage and MMSE score. (O) SHAP summary plots showing the contribution of each retinal biomarker to model output, ranked by mean absolute SHAP value. Each dot represents one sample and is colored by feature value. (P) Feature robustness across 1,000 independently initialized models. Left‐blue bars indicate the fraction of iterations in which a feature was ranked among the top five by mean absolute SHAP value; right‐orange bars indicate the mean absolute SHAP value averaged across iterations. GCL, ganglion cell layer; IPL, inner plexiform layer; INL, inner nuclear layer; OPL, outer plexiform layer; ONL, outer nuclear layer.

Given emerging evidence that neuroinflammation and microglia actively regulate synaptic remodeling and elimination in neurodegenerative disease, we first investigated whether retinal gliosis is associated with synaptic loss and UCHL1 dysregulation in AD (Figure 6A–H). Representative retinal immunofluorescence images demonstrated progressive activation of IBA1‐positive microglia and GFAP/vimentin‐positive macroglia in MCI and AD retinas, occurring at sites of synaptic loss identified by reduced synaptophysin immunoreactivity (Figure 6A,B). Quantification of retinal inflammatory markers confirmed robust activation of both micro‐ and macroglial compartments. IBA1‐positive microglia increased approximately 2.0–2.4‐fold in MCI and AD retinas compared with NC controls, whereas GFAP‐ and vimentin‐positive macroglia increased by approximately 1.4–2.8‐fold, indicating progressive activation of retinal astrocytes and Müller glia (Figure 6C). Correlation analyses revealed strong inverse associations between glial activation, glutamatergic synaptic integrity, and UCHL1 (Figure 6D–H). Specifically, increased retinal IBA1 immunoreactivity was closely associated with reductions in VGLUT1 (r = −0.73, P*_adj_* < 0.0001), synaptophysin (r = −0.71, P*_adj_* < 0.0001), PSD95 (r = −0.68, P*_adj_* < 0.0001), NMDAR2A (r = −0.69, P*_adj_* < 0.0001), and UCHL1 (r = −0.62, P*_adj_* < 0.0001) (Figure 6D,E). Similar inverse relationships were observed for retinal GFAP and vimentin, with vimentin exhibiting particularly strong correlations with all synaptic markers, especially presynaptic VGLUT1 (r = −0.82), synaptophysin (r = −0.84), and UCHL1 (r = −0.79; all P*_adj_* < 0.001–0.0001) (Figure 6D,F–H). These inflammatory markers, IBA1, GFAP, and vimentin, further exhibited direct associations with retinal p75NTR expression (r = 0.76–0.77; P*_adj_* < 0.001), linking inflammatory activation to Aβ‐responsive neurodegenerative pathways (Figure S11A).

To further elucidate the relationship between neuroinflammation and synaptic degeneration, we interrogated retinal proteomic datasets from the independent protein biochemistry cohort (Figure 6I–M and Figure S11B–D). Similar to the histological measures, retinal synaptic proteins exhibited strong inverse correlations with markers of activated microglia and disease‐associated microglia (DAM), including allograph inflammatory factor 1 (AIF1, IBA1), integrin subunit alpha M (ITGAM, CD11b), integrin subunit beta 2 (ITGB2, CD18), integrin subunit alpha X (ITGAX, CD11c), glycoprotein non‐metastatic melanoma protein B (GPNMB), cytochrome b‐245 beta chain (CYBB, NOX2), APOE, and proteasome subunit beta type‐8 (PSMB8). In particular, the postsynaptic PSD95 demonstrated close inverse relationships with IBA1 (r = −0.78, P = 0.0046), GPNMB (r = −0.71, P = 0.0149), and CYBB (r = −0.79, P = 0.0037), whereas the presynaptic synaptophysin was strongly and inversely correlated with IBA1 (r = −0.71, P = 0.0149) and ITGB2 (r = −0.77, P = 0.0060; Figure 6I; extended pairwise analyses in Figure S11B,C). Notably, synaptophysin and PSD95 showed strong positive associations with Aβ‐degrading enzymes, angiotensin‐converting enzyme (ACE; r = 0.86, P = 0.0006) and insulin‐degrading enzyme (IDE; r = 0.69, P = 0.02; Figure 6J,K), expressed by astrocytes and/or microglia, linking glial activation and impaired Aβ‐clearance to synaptic loss. We also examined the relationship between the oxidized UCHL1 form detected by MS and inflammatory pathways, and observed strong direct correlations with microglial‐associated proteins including IBA1 (r = 0.77, P = 0.0036), ITGB2 (r = 0.76, P = 0.0038), PSMB8 (r = 0.62, P = 0.0298), and the glial pyroptosis markers gasdemin D (GSDMD; r = 0.65, P = 0.0209) and gasdermin E (GSDME; r = 0.87, P = 0.0002) (Figure 6L,M and Figure S11D). These findings consistently demonstrate that retinal synaptic degeneration and UCHL1 dysregulation occur within a broader neuroinflammatory environment characterized by DAM and macroglial activation, oxidative stress, and inflammatory cell death.

### 2.7 Machine Learning Identifies Retinal UCHL1 as a Predictor of Braak Stage and Cognition

We next assessed the relationship between the above‐studied retinal neurodegeneration or synaptic biomarkers and retinal/brain atrophy in our human cohorts (Figure 6N). While all retinal biomarkers significantly correlated with retinal atrophy locally, only retinal UCHL1, p75NTR, and postsynaptic markers correlated with tissue atrophy in both retina and respective brains (Figure 6N), suggesting that p75NTR death receptor signaling and UCHL1 contribute to widespread synaptopathy and tissue atrophy observed in both CNS compartments in AD. These results prompted us to investigate the disease predictability of multiple retinal biomarkers using machine learning (Figure 6O,P). To this end, we trained Random Forests [130] to predict Braak stage and MMSE score. Learning curves indicated that the model predicting Braak stage likely had sufficient data, but that the one for MMSE retained significant differences across folds (Figure S12A,B). Both Random Forest models were trained with 80 estimators and L1 loss and used an identical retinal biomarker feature set. The mean R^2^ score across five‐fold cross‐validation was 0.370 for predicting Braak stage, and 0.137 for predicting MMSE. Notably, among all dysregulated retinal proteins, UCHL1, and also PSD95, VGLUT1, and GFAP emerged as the top predictors of both Braak stage and MMSE score (Figure 6O); retinal AβOi (C/M) was predictive of Braak stage but not MMSE, and retinal Nissl signal and MC1 (tau tangles) were predictive of MMSE but not Braak. To mitigate sensitivity to initialization, we refit each model across 1,000 random seeds and quantified feature robustness by the consistency of their SHAP‐derived importance across runs (Figure 6P). The impact of each feature on model prediction varied considerably, but UCHL1 was both consistently identified and had the greatest impact. Overall, these results identify retinal UCHL1 deficiency as an early event linking Aβ proteinopathy to proteostasis disruption and synaptic degeneration in AD and highlight the potential of non‐invasive retinal synaptic and/or UCHL1 biomarkers for monitoring disease progression and cognitive decline.

## 3 Discussion

The present study identifies profound glutamatergic synaptic degeneration and marked UCHL1 dysregulation in the retinas of individuals with MCI and AD, establishing these alterations as early pathological features closely associated with disease severity and cognitive decline. Across independent cohorts and complementary methodologies, including spatial histopathology, proteomics, biochemistry, ultrastructural analyses, machine learning, and neuronal culture models, we demonstrate reductions in excitatory pre‐ and postsynaptic proteins, ribbon synapse disruption, aberrant p75NTR signaling, oxidative stress, and neuroinflammatory activation. These changes were strongly associated with retinal Aβ_42_ and pathogenic tau accumulation, glial activation, brain pathology, and cognitive impairment. Retinal UCHL1 emerged as a particularly robust correlate of synaptic integrity, disease‐associated glial responses, and clinical severity, and was identified as the strongest predictor of Braak stage and MMSE score. Importantly, UCHL1 reduction was already evident in cognitively normal individuals with elevated retinal Aβ_42_ burden and was reproduced by fibrillar Aβ_42_ in neuronal cultures before detectable neuronal loss, supporting UCHL1 depletion as an early Aβ‐induced event linked to synaptic vulnerability rather than a secondary consequence of neurodegeneration.

A major finding was the early and severe loss of glutamatergic synapses, which remained significant after accounting for thinning of the corresponding neuronal retinal layers, indicating that synaptic degeneration emerges at least as early as the MCI stage. VGLUT1 and NMDAR2A exhibited particularly pronounced reductions. Ultrastructural analyses further revealed ribbon synapse degeneration, vesicle disorganization, and disruption of pre‐ and postsynaptic architecture within the plexiform layers. These deficits were most prominent in mid‐ and far‐peripheral retina, paralleling the preferential peripheral accumulation of retinal pathogenic Aβ and tau forms reported in AD [23, 27, 28, 30, 37, 131]. The persistence of synaptic‐Aβ_42_ associations in NC/MCI‐only analyses, together with Aβ_42_‐induced synaptic marker loss in human and primary neuronal cultures without neuronal death, supports the concept that synaptic degeneration is an early and partially independent pathological process preceding overt neurodegeneration. Notably, immature and oligomeric tau species correlated more strongly with synaptic loss than mature tau filaments and tangles, consistent with evidence that soluble tau species exert disproportionate synaptotoxic effects [132, 133, 134].

These findings align with growing evidence that synaptic dysfunction represents one of the earliest and most clinically relevant manifestations of AD. In the brain, synapse loss often precedes overt neuronal degeneration and correlates more closely with cognitive decline than neuronal death itself [2]. Converging experimental and biomarker studies further suggest that excitatory synapses are particularly vulnerable during the earliest stages of AD, although the temporal relationship between synaptic and neuronal degeneration may vary across brain regions [135, 136]. Consistent with this concept, CSF studies have demonstrated reductions in multiple synaptic proteins, including Calsyntenin‐1, glutamate receptor 4 (GluR4), Neurexin‐2A, Neurexin‐3A, Syntaxin‐1B, and thymocyte differenciation antigen 1 (Thy‐1), during preclinical AD, before the appearance of clinical symptoms or canonical CSF markers of neurodegeneration [137]. Similarly, in the 5xFAD mouse brain, layer V neurons exhibit early synaptic deficits that emerge before detectable structural dystrophy [138]. Our retinal findings parallel these observations and extend them to the visual system, supporting the concept that synaptic degeneration represents an early and partially independent pathological process. Comparable synaptopathic changes have been reported in retinal degeneration, multiple sclerosis (MS), and Parkinson's disease (PD) models, where retinal synaptic dysfunction precedes structural neuronal loss. In retinal degeneration models, synaptic abnormalities arise before photoreceptor degeneration [139], whereas in chronic MS models, synaptic injury and IPL thinning occur before neuronal loss and represent early features of inflammatory demyelination [54]. Likewise, in PD models, altered retinal synaptic signaling precedes overt neurodegeneration and progresses toward cone‐mediated circuit vulnerability [140]. Consistent with these experimental findings, OCT studies in patients with AD [141, 142, 143] and multiple AD mouse models [144, 145, 146, 147] have consistently demonstrated early thinning of synapse‐rich retinal layers, particularly the IPL and OPL, supporting the retina as a vulnerable and accessible site of early synaptic pathology.

Proteomic analyses revealed a dual vulnerability of the AD retina characterized by synaptic failure and photoreceptor degeneration. Broad reductions in phototransduction‐related proteins indicated photoreceptor dysfunction, while synaptic pathway analyses identified widespread disruption of vesicle cycling, synaptic plasticity, and dendritic spine regulation [148, 149, 150, 151, 152, 153, 154]. Reduced levels of synaptophysin, unc‐13 homolog A (UNC13A), syntaxin‐binding protein 1 (STXBP1), adenylate cyclase 1 (ADCY1), erythropoietin‐producing hepatocellular receptor B2 (EPHB2), GNB5, and PSD95, together with increased expression of proteins implicated in synaptic endocytosis [151, 155, 156], suggest impaired neurotransmitter release and vesicle recycling. These findings are consistent with disrupted retinal excitatory transmission and functional circuit disconnection, previously described in cortical and hippocampal AD networks [157, 158, 159, 160, 161].

Among the most prominent molecular alterations in the AD retina was robust upregulation of p75NTR, a death receptor that regulates axon pruning, synaptic plasticity, and apoptosis in the CNS [92, 162]. In AD models, Aβ binding to p75NTR promotes neuritic degeneration, tau hyperphosphorylation, oxidative stress, and neuronal loss [91, 93, 94], and consistent with these observations, elevated retinal p75NTR in our AD cohorts closely correlated with reduced synaptic markers (VGLUT1, PSD95), increased Aβ_42_ burden, immature tau species, and oxidative stress. Given the ability of Aβ to directly activate p75NTR‐dependent degenerative signaling [95, 105, 106], our findings support a model in which p75NTR functions as a junction for Aβ‐, tau‐, and ROS‐mediated stress pathways that culminate in synaptic degeneration. These results are further supported by previous studies implicating p75NTR in retinal neuritic degeneration, tau pathology, and neuronal loss in AD [93, 94, 163]. Notably, p75NTR expression was not restricted to neurons but was also detected in GFAP‐ and vimentin‐positive macroglia in MCI and AD retinas, consistent with reactive astrocyte and Müller glial activation. Given the critical roles of macroglia in synaptic homeostasis, neuroinflammatory signaling, and neuronal support, p75NTR upregulation in these cells may contribute to disease‐associated glial remodeling and influence synaptic vulnerability [24, 59]. Together, these findings suggest that p75NTR links neuronal and glial pathogenic responses in the AD retina and may represent a key mediator through which proteinopathy, oxidative stress, and neuroinflammation converge to drive retinal synaptopathy.

The close relationship between neuroinflammation and synaptic degeneration was another key observation. Although retinal gliosis has been documented in AD [24, 28, 39, 68, 164], its functional relationship to retinal pathology remains poorly understood. Here, increased retinal IBA1, GFAP, and vimentin expression exhibited strong inverse correlations with key excitatory synaptic markers (VGLUT1, synaptophysin, PSD95, NMDAR2A), indicating that retinal synaptopathy develops within an active inflammatory milieu. Consistent with evidence that activated microglia regulate synaptic remodeling and mediate complement‐dependent synaptic engulfment in AD [58, 61, 64], retinal synaptic markers were strongly associated with DAM‐related proteins (GPNMB, ITGB2, ITGAX, APOE, ITGAM, IBA1), supporting analogous neuroimmune mechanisms in the retina. Furthermore, correlations of synaptophysin and PSD95 with glial‐derived oxidative stress mediators (CYBB, PSMB8) and Aβ‐degrading inflammatory enzymes (ACE, IDE) [127, 128, 129, 165], astrocytic glutamate‐regulating proteins (solute carrier family 1 member 2 ‐ SLC1A2, aquaporin 4 ‐ AQP4), and pyroptosis markers (GSDMD, GSDME) suggest that retinal synaptic degeneration occurs within a complex multicellular inflammatory network involving micro‐ and macro‐glial pathways. Together, these findings support a role for neuroimmune remodeling as a major contributor to retinal synaptic vulnerability in AD and warrant further investigation of microglial and complement‐mediated synaptic pruning mechanisms.

A central and novel aspect of this study is the identification of retinal UCHL1 dysregulation as a major component of AD‐associated synaptopathy. UCHL1, a neuron‐enriched deubiquitinating enzyme essential for proteostasis and synaptic integrity [72, 166], was markedly reduced in MCI and AD retinas, particularly within synapse‐rich layers (IPL and OPL), whereas proteomic analyses revealed accumulation of oxidized, aggregation‐prone forms. This pattern mirrors observations in AD brain tissue, where oxidative stress promotes UCHL1 oxidation and aggregation [118, 119], accompanied by reductions in soluble UCHL1 levels and activity [121]. Because the antibody used in this study primarily recognizes the soluble cytosolic form of UCHL1, which is also reduced in AD and PD brains [71, 167], the decreased retinal signal detected by histological and biochemical analyses likely reflects loss of functional soluble UCHL1, whereas mass spectrometry likely captures oxidized species. This imbalance parallels reports linking UCHL1 dysfunction to impaired Aβ and tau clearance [168, 169] and suggests a pathogenic shift from soluble, active UCHL1 toward oxidized, aggregation‐prone forms, thereby compromising proteostatic resilience and contributing to synaptic vulnerability in the AD retina.

Retinal UCHL1 exhibited strong positive associations with synaptic markers and cognitive performance and inverse relationships with Aβ_42_, tau pathology, oxidative stress, p75NTR, and glial activation. Aβ_42_ fibrils induced early UCHL1 depletion together with synaptic marker loss in neuronal cultures while neuronal structural markers remained preserved, supporting a role for UCHL1 dysregulation in early Aβ‐driven synaptic injury. Consistent with this interpretation, retinal UCHL1 reduction was already detectable in cognitively normal individuals with elevated retinal Aβ_42_ burden and preserved retinal thickness. Network analyses further linked UCHL1 to autophagy, axonal transport, and synaptic recycling pathways, positioning it at the intersection of proteostatic dysfunction, neuroinflammation, oxidative stress, and synaptic degeneration. Although causality remains to be established, these findings suggest that UCHL1 may represent a critical regulator connecting multiple pathogenic pathways in AD.

Cell type‐specific analyses indicated that retinal UCHL1 reduction in AD is not pan‐neuronal but preferentially affects interneuron populations, particularly horizontal and bipolar cells. This selective vulnerability is biologically plausible given the high demands that ribbon synapses place on vesicle turnover, synaptic maintenance, and proteostatic control [170, 171]. Marked reductions of UCHL1 in parvalbumin‐, calbindin‐, and PKCα‐positive neurons, together with parallel losses of VGLUT1 and Bassoon in the OPL and IPL, support a model of coordinated presynaptic failure in which impaired glutamatergic transmission and diminished proteostatic resilience converge at vulnerable retinal circuits. These findings are consistent with evidence that UCHL1 localizes to glutamatergic presynaptic compartments adjacent to VGLUT1 [172] and with previous reports demonstrating retinal neuronal UCHL1 expression [123, 124, 125, 126]. The parallel reduction of UCHL1 and VGLUT1 in MCI and AD retinas, as well as in Aβ‐exposed neuronal cultures, further supports coordinated dysfunction of glutamatergic vesicle handling and proteostasis at vulnerable synapses. Although in cognitively normal individuals UCHL1 was also detected in retinal ganglion cells, amacrine cells, and photoreceptors, particularly cones, consistent with prior immunolabeling [173, 174] and single‐cell transcriptomic studies [175, 176], the preferential involvement of interneuron‐rich synaptic networks identifies horizontal and bipolar cells as potential early substrates of retinal synaptopathy in AD.

The relationship between neuroinflammation and UCHL1 was particularly noteworthy. Although UCHL1 is classically regarded as a neuronal proteostasis regulator, its activity is highly sensitive to inflammatory and oxidative stress conditions [117, 119]. The strong associations observed here between UCHL1, glial activation, inflammatory pathways, and synaptic markers suggest that retinal UCHL1 deficiency reflects not only synaptic dysfunction but broader neuroimmune dysregulation. Together with the ability of fibrillar Aβ_42_ to induce UCHL1 depletion and synaptic deficits in neuronal cultures in the absence of glia, these findings support a model in which UCHL1 occupies a mechanistic intersection between Aβ accumulation, oxidative stress, inflammatory activation, and synaptic degeneration, with a neuron‐intrinsic component of vulnerability that is likely amplified by neuroinflammatory processes in vivo.

Consistent with this central role, retinal UCHL1 and synaptic markers correlated strongly with Braak stage, brain atrophy, and cognitive impairment (CDR and MMSE), and artificial intelligence (AI)‐based multivariable modeling identified retinal UCHL1 as the strongest predictor of disease stage and cognition. Although these findings do not establish causality, they highlight the retina as a uniquely accessible site for interrogating synaptic, proteostatic, and neuroinflammatory dysfunction in AD and support the clinical development of multimodal retinal biomarkers. In this context, pattern ERG may provide a sensitive functional readout of early synaptic impairment [52], particularly when combined with OCT assessment of synapse‐rich retinal layers, including the IPL and GCIPL [53, 54, 55], while adaptive optics imaging offers a promising approach for near‐cellular‐resolution evaluation of AD‐related retinal microstructural abnormalities in vivo [56, 57].

Several limitations should be acknowledged. The cross‐sectional nature of postmortem analyses precludes direct assessment of temporal progression and causality. Although neuronal culture experiments support an Aβ‐driven mechanism for UCHL1 depletion and synaptic dysfunction, they do not establish whether UCHL1 loss itself is sufficient to drive degeneration. Cohort size, while comparatively large for retinal AD studies, remains modest for machine‐learning analyses. In addition, the contribution of vascular pathology, co‐pathologies, and microglial and complement‐mediated synaptic pruning requires further investigation. Longitudinal studies integrating retinal imaging, functional electrophysiology, CSF synaptic biomarkers, and UCHL1‐targeted approaches will be important for validation and clinical translation.

In summary, this study identifies the retina as an early site of UCHL1 dysregulation, proteostatic disruption, and glutamatergic synaptic loss in MCI and AD patients. The convergence of Aβ and tau pathology, oxidative stress, neuroinflammation, p75NTR signaling, and Aβ‐induced UCHL1 depletion supports an interconnected pathogenic network underlying retinal and cerebral neurodegeneration. By establishing retinal UCHL1 as a strong indicator of disease severity and cognitive decline, these findings provide a foundation for retinal synaptic biomarkers with potential utility for mechanistic studies and disease monitoring.

## 4 Experimental Section

### 4.1 Postmortem Human Eye and Brain Samples

Postmortem human eye globes and brain tissues were obtained from the Alzheimer's Disease Research Center (ADRC) Neuropathology Core in the Department of Pathology at the University of Southern California (USC, Los Angeles, CA; IRB protocol HS‐042071). In addition, eye globes were obtained from the National Disease Research Interchange (NDRI, Philadelphia, PA; IRB exempt protocol EX‐1055). For a subset of patients and controls, brain specimens were also obtained from the ADRC Neuropathology Core at the University of California, Irvine (UCI [IRB protocol HS#2014–1526]). USC‐ADRC, NDRI, and UCI ADRC maintain human tissue collection protocols that are approved by their respective governing committees and are subject to oversight by the National Institutes of Health and institutional guidelines. All histological procedures were conducted at Cedars‐Sinai Medical Center under IRB protocols (Pro00053412, Pro00019393, and Pro00055802). For histological examinations, 53 retinas were collected from deceased donors with confirmed AD (*n*  =  21) or MCI due to AD (*n*  =  11), and from age‐ and sex‐matched deceased donors with NC (*n*  =  21). For analysis of retinal proteins using MS‐based proteomics, ELISA, and WB, fresh‐frozen retinas were collected from another cohort of deceased donors (*n*  =  16) with clinically and neuropathologically confirmed AD (*n*  =  9) and matched NC controls (*n*  =  7). Comprehensive cohort details are presented in Table 1 and Table S1. The human cohort used in this study exhibited no significant differences in age, sex, or postmortem interval (PMI) duration. Patients' confidentiality was maintained by de‐identifying all tissue samples, ensuring that donors could not be identified.

### 4.2 Clinical and Neuropathological Assessments

The detailed clinical and neuropathological assessment procedures are described in our recent publication [28, 37]. In summary, clinical and neuropathological reports detailing patients' neurological examinations and neuropsychological and cognitive assessments were generously provided by the ADRC system through the Unified Data Set [177]. The NDRI provided patient information, including sex, ethnicity, age at death, cause of death, medical history indicating AD, the presence or absence of dementia, and any accompanying medical conditions. Most cognitive assessments were conducted annually, typically within one year prior to death. In this study, we utilized cognitive scores obtained closest to the patient's time of death. Three global indicators of cognitive status were used for clinical assessment: the Clinical Dementia Rating (CDR scores: 0 = normal; 0.5 = very mild impairment; 1 = mild dementia; 2 = moderate dementia; or 3 = severe dementia) [178], the Montreal Cognitive Assessment (MOCA scores: ≥26 = cognitively normal or <26 = cognitively impaired [179, 180], and the Mini‐Mental State Examination (MMSE scores: normal cognition = 24–30; MCI = 20 – 23; moderate dementia = 10– 19; or severe dementia ≤ 9) [181].

The assessment of cerebral Aβ burden comprised analysis of diffuse and neuritic plaques (including both immature and mature forms), along with amyloid angiopathy, neurofibrillary tangles (NFTs), neuritic threads (NTs), granulovacuolar degeneration, Lewy bodies, Hirano bodies, Pick bodies, ballooned neurons, neuronal loss, microvascular changes, and gliosis. These evaluations were conducted across multiple brain regions, notably in the hippocampus, the entorhinal cortex, the superior frontal gyrus in the frontal lobe, the superior temporal gyrus in the temporal lobe, the superior parietal lobule in the parietal lobe, the primary visual cortex, and the visual association area in the occipital lobe. All brain samples were uniformly collected by a neuropathologist.

Formalin‐fixed, paraffin‐embedded brain sections were used to determine the severity of amyloid plaques and NFTs in the brain using anti‐Aβ monoclonal antibody (mAb) clone 4G8, anti‐phospho‐tau mAb clone AT8, Thioflavin‐S (ThioS), and Gallyas silver staining. Two neuropathologists independently rated the burden of Aβ, NFTs, and NTs on a scale of 0, 1, 3, and 5 [(0 = none, 1 = sparse (0–5), 3 = moderate (6–20), 5 = abundant/frequent (21–30 or above), N/A = not applicable)], with final scores calculated as the average of the two readings. The final diagnosis included AD neuropathologic change. The Aβ plaque scoring system was adapted from Thal et al., (A0 = no Aβ or amyloid plaques, A1 = Thal phase 1 or 2, A2 = Thal phase 3, and A3 = Thal phase 4 or 5) [182]. NFT staging was adjusted from Braak for silver‐based histochemistry or p‐tau immunohistochemistry (B0 = no NFTs, B1 = Braak stage I or II, B2 = Braak stage III or IV, B3 = Braak stage V or VI) [183]. The neuritic plaque score was adapted from CERAD (C0 = no neuritic plaques, C1 = CERAD sparse, C2 = CERAD moderate, C3 = CERAD frequent) [184]. Additional evaluations included neuronal loss, gliosis, granulovacuolar degeneration, Hirano bodies, Lewy bodies, Pick bodies, and ballooned neurons, using hematoxylin and eosin staining, with scores of 0 for absent and 1 for present. Amyloid angiopathy was classified into 4 grades: Grade I indicates amyloid around normal/atrophic smooth muscle cells of vessels; Grade II shows media replaced by amyloid without blood leakage; Grade III involves extensive amyloid deposition with vessel wall fragmentation and perivascular leakage; Grade IV includes extensive amyloid deposition with fibrinoid necrosis, microaneurysms, mural thrombi, lumen inflammation, and perivascular neuritis.

### 4.3 Collection and Processing of Eye Tissues

Donor eyes were obtained within an average of 7.9 h from the time of death. For histological and TEM analyses, eyes were punctured once at the limbus, fixed in either 10% neutral buffered formalin or 4% paraformaldehyde (PFA), and stored at 4°C. Freshly collected eyes for biochemical analyses were preserved in Optisol‐GS media (Bausch & Lomb, 50006‐OPT) and stored at 4°C for less than 36 h, after which they were snap‐frozen and stored at ‐80°C. These procedures were uniformly performed across all tissue sources and collection sites.

### 4.4 Preparation of Retinal Tissues and Cross‐Sections

Eyes that were either freshly preserved in Optisol‐GS or fixed were dissected on ice. After removal of the anterior chamber to obtain eyecups, the vitreous humor was carefully cleared. Retinas were then meticulously isolated, separated from the choroid, and prepared as flatmounts following established procedures [27, 28, 37]. Flatmount strips (∼2 mm wide) extending from the ora serrata to the optic disc were dissected to generate four strips: superior‐temporal (ST), inferior‐temporal (IT), inferior‐nasal (IN), and superior‐nasal (SN). The ST and IT strips analyzed in this study, generated from fixed retinas, were embedded in paraffin, rotated 90° horizontally, and re‐embedded in paraffin blocks. These retinal strips, encompassing central, mid, and far retinal subregions, were sectioned at 8–10 µm and mounted on microscope slides treated with 3‐Aminopropyltriethoxysilane (APES, Sigma A3648). Temporal hemiretinas dissected from fresh tissue were stored at − 80°C for downstream protein analysis (MS, ELISA, WB). Together, these preparation methods provided consistent and comprehensive access to retinal quadrants, layers, and pathological subregions.

### 4.5 IHC Analysis

Before the IHC procedure, paraffin‐embedded retinal cross‐section slides were deparaffinized in 100% xylene twice (10 min each), rehydrated through decreasing concentrations of ethanol (100% to 70%), and washed with distilled water followed by PBS. Antigen retrieval was performed by incubating retinal cross‐sections in target retrieval solution (pH 6.1; S1699, DAKO) at 99°C for 1 h, followed by PBS washes. In some staining cases, sections were additionally treated with 70% formic acid (ACROS) for 10 min at room temperature (RT).

#### 4.5.1 Peroxidase‐Based IHC

Labeling was performed using the Vectastain Elite ABC HRP kit (Vector, PK‐6101, Peroxidase rabbit IgG) according to the manufacturer's instructions. In summary, after incubation with 3% H_2_O_2_ for 20 min, sections were washed in PBS and incubated with blocking serum containing 0.25% Triton X‐100 (Sigma, T8787) for 45 min at RT. Primary antibodies (Table 5) were diluted in PBS containing blocking serum and applied overnight at 4°C. The following day, sections were washed three times in PBS, incubated with secondary antibody (Table 5) for 30 min at 37°C, washed three times again in PBS, and incubated with ABC reagent for 30 min at RT. After PBS washes, signal was visualized with 3,3′‐diaminobenzidine (DAB) substrate (DAKO K3468). Hematoxylin counterstaining was performed, and slides were mounted with Paramount aqueous mounting medium (DAKO, S3025). Control sections were processed without the primary antibodies to assess nonspecific labeling.

**TABLE 5:** Key resources table.

*Primary antibody*	Source Species	Dilution	Application	Commercial Source	Catalog number
Bassoon mAb (SAP7F407)	Mouse	1:400	IF	Enzo Life Sciences	ADI‐VAM‐PS003‐D; RRID: AB_2038857
Parvalbumin pAb	Goat	1:200	IF	Novus Biological	NB100‐1541; RRID:AB_2173902
Calbindin D‐28K Clone CB‐955 mAb	Mouse	1:1000	IF	Sigma–Aldrich	C9848; RRID:AB_476894
PKC alpha mAb	Rabbit	1:250	IF	abcam	ab32376; RRID:AB_777294
βIII‐tubulin (TUBB3)	Mouse	1:200	IF	abcam	ab78078; RRID: AB_2256751
β‐actin	Goat	1:200	IF	abcam	ab8229; RRID: AB_ 306374
NeuN [EPR12763] mAb	Rabbit	1:1000	WB	abcam	Ab177487; RRID:AB 2532109
PSD95 mAb	Rabbit	1:600	IF/WB/TEM	abcam	ab76115; RRID: AB_1310620
VGLUT1 pAb	Guinea Pig	1:1000	IF/TEM	EMD Millipore	AB5905; RRID: AB_2301751
Synaptophysin mAb	Mouse	1:1000	IF/WB	Biolegend	837101; RRID: AB_2565370
NMDAR2A pAb	Rabbit	1:1000	IF	NovusBio	NB300‐105; RRID: AB_10001400
p75NGFR (p75NTR) mAb	Rabbit	1:100	IF/WB	abcam	ab52987; RRID: AB_881682
UCHL‐1 (PGP9.5) mAb	Rabbit	1:1250	IF/DAB/WB	abcam	ab108986; RRID: AB_10891773
12F4 (Aβ_42_) mAb	Mouse	1:200	IF	Biolegend	805503; RRID: AB_2564682
scFvA13 (Oligo‐Aβ) mAb	Mouse	1:450	IF	Dr. Giovanni Meli Lab	−
Ser396 (pS396‐tau) pAb	Rabbit	1:1500	IF	Anaspec	AS‐54977
AT8 (Ser202/Thr205) phospho‐tau) mAb	Mouse	1:250	IF	ThermoFisher	MN1020; RRID: AB_223647
T22 (Oligo‐tau) pAb	Rabbit	1:200	IF	Dr. Rakez Kayed Lab	−
CitR_209_‐tau	Mouse	1:5000	IF	Daniel Lee Lab	−
PHF‐1(Ser396/Ser404) phospho‐tau mAb	Mouse	1:200	IF	Dr. Peter Davies Lab	−
MC‐1 (NFT) mAb	Mouse	1:200	IF	Dr. Peter Davies Lab	−
IBA1 pAb	Rabbit	1:400	IF	Wako	019‐19741; RRID: AB_839504
IBA1 pAb	Goat	1:300‐500	IF	R&D System	NB100‐1028; RRID: AB_521594
GFAP mAb (2.2B10)	Goat	1:500	IF	Invitrogen	13‐0300; RRID: AB_2532994
GFAP pAb	Goat	1:500	IF	R&D System	NB100‐53809; RRID: AB_829022
Vimentin mAb	Mouse	1:200	IF	abcam	ab28028; RRID: AB 778826
GAPDH (D16H11) mAb	Rabbit	1:1000	WB	Cell signaling	5174; RRID: AB_10622025
GAPDH mAb	Mouse	1:1000	WB	Millipore Sigma	G8795 RRID: AB_1078991
***Secondary antibody***						
Cy2 (anti‐ mouse, Rabbit)	Donkey	1:200	IF	Jackson ImmunoResearch Laboratories		
Cy3 (anti‐ mouse, Guinea pig, Rabbit)	Donkey	1:200	IF			
Cy5 (anti‐ mouse, Guinea pig, Rabbit)	Donkey	1:200	IF			
IRDye 680RD pAb	Rabbit	1:10000	WB	Licorbio	926‐68071 RRID: AB_10956166
IRDye 800CW pAb	Rabbit	1:10000	WB	Licorbio	926‐32211 RRID: AB_621843
IRDye 680RD pAb	Mouse	1:10000	WB	Licorbio	926‐68070 RRID: AB_10956588
IRDye 800CW pAb	Mouse	1:10000	WB	Licorbio	926‐32210 RRID: AB_621842
HRP anti‐rabbit	Goat	1:200	IHC‐DAB	Vectastain Elite ABC‐HRP kit	PK‐6101
***Experimental models: Cell lines***						
SH‐SY5Y	Human	−	ICC/WB/mRNA	ATCC	CRL‐2266^TM^	
***Experimental models: Organisms***						
C57BL/6J	Mouse	−	Primary hippocampal neurons	The Jackson Laboratory	000664 RRID:IMSR_JAX:000664	
***Oligonucleotides***						
*SYP*: forward: 5′‐TTGGCTTCGTGAAGGTGCTGCA‐3′ *SYP*: reverse: 5′‐ACTCTCCGTCTTGTTGGCACAC‐3′ *TUBB3*: forward 5′‐TCAGCGTCTACTACAACGAGGC‐3′ *TUBB3*: reverse 5′ GCCTGAAGAGATGTCCAAAGGC‐3′) *UCHL1*: forward *5*′‐CAGTTCAGAGGACACCCTGCTG‐3′; *UCHL1*: reverse 5′‐CCACAGAGCATTAGGCTGCCTT‐3′ *GAPDH*: forward: 5′‐ACCACAGTCCATGCCATCAC‐3′ *GAPDH*: reverse: 5′‐TCCACCACCCTGTTGCTGTA‐3′						
***Key reagents and kits***						
Cresyl Violet acetate (Nissl stain)	−	0.1%	Histology	Sigma‐Aldrich	C5042	
Dihydroethidium (DHE)	−	5‐10µM	Histology	Sigma‐Aldrich	D7008	
3,3′‐diaminobenzidine (DAB)	−	−	Histology	DAKO	K3468	
3‐Aminopropyltriethoxysilane (APES)	−	2%	Slide coating	Sigma‐Aldrich	A3648	
Fluoromount‐G, with DAPI	−	−	Histology	ThermoFisher	00‐4959‐52	
Human Amyloid beta 42 ELISA Kit	−	−	ELISA	ThermoFisher	KHB3441	
4–20% precast polyacrylamide gels	−	−	WB	Bio‐Rad	4561094	
***Chemicals, peptides, and recombinant proteins***						
Beta‐amyloid (Aβ_1‐42_)	−	−	Cell culture	Anaspec	AS‐20276	
***Software and algorithms***						
GraphPad Prism (Version 10.0.3)	GraphPad				http://www.graphpad.com; RRID: SCR_002798	
Zeiss Zen Lite	Zeiss				https://www.zeiss.com/microscopy/us/products/software/zeiss‐zen‐lite.html	
Fiji ImageJ (Version 2.14.0/1.54f/Java 1.8.0‐322)	Fiji				*open‐source download*	
Image Studio software	Licorbio				https://www.licorbio.com/image‐studio	
Metascape	https://metascape.org/				*open source*	
Cytoscape 3.10.2	https://cytoscape.org/				*open source*	
ClustVis	https://biit.cs.ut.ee/clustvis/				*open source*	
Ingenuity Pathway Analysis (IPA)	*Qiagen*				https://digitalinsights.qiagen.com/	
SynGO	https://syngopotal.org/				*open source*	
String 12.0	https://string‐db.org/				*open source*	
*Deposited data*						
Mass spectrometry dataset	*ProteomeXchange Consortium via the PRIDE* https://www.ebi.ac.uk/pride/archive/projects/PXD040225 https://ftp.pride.ebi.ac.uk/pride/data/archive/2025/10/PXD040225				*PXD040225*	
Machine learning	*Scikit‐learn* *NumPy* *pandas* *SciPy* *Jupyter* [188]				https://scikit‐learn.org/stable/index.html https://numpy.org/ https://pandas.pydata.org/ https://scipy.org/ https://jupyter.org/	

Abbreviations: CitR209‐tau, Citrullinated arginine (R)‐209 tau; Cyanine dyes, Cy2, Cy3, Cy5; HRP, Horseradish peroxidase; IF, immunofluorescence; mAb, monoclonal antibody; NFT, Neurofibrillary tangles; NGFR, Nerve growth factor receptor; NMDAR2A, N‐methyl‐D‐aspartate receptor; pAb, polyclonal antibody; PHF, Paired helical filament; PSD95, Postsynaptic density 95; UCHL‐1, Ubiquitin carboxyl‐terminal hydrolase L1; VGLUT‐1, Vesicular glutamate transporter.

#### 4.5.2 Fluorescence‐Based Immunostaining

Sections were treated with blocking solution (DAKO X0909) containing 0.25% Triton X‐100 (Sigma, T8787) for 1 h at RT, followed by overnight incubation at 4°C with primary antibodies diluted in the same blocking solution. The following day, after PBS washes, sections were incubated with fluorophore‐conjugated secondary antibodies (Table 5) for 1 h at RT. After PBS washes, sections were mounted using Fluoromount‐G, with DAPI (Invitrogen, #00‐4959‐52). Control sections processed without the primary antibodies were used to assess nonspecific fluorescence.

### 4.6 Nissl Staining

Neuronal cytoplasm was specifically stained with the Nissl staining technique. Deparaffinized and rehydrated sections were stained in 0.1% Cresyl Violet acetate (Sigma #C5042) for 5 min, rapidly rinsed in tap water, and briefly dipped in 70% ethanol. Sections were then dehydrated through two changes of absolute ethanol for 3 min each, followed by immersion in xylene twice for 2 min, and mounted using xylene‐based mounting medium (Fisher Scientific Company, L.L.C. #245–691). An average of 12 images per section, covering the retinal neurons from the optic disc to the ora serrata, were captured at a 20x objective and analyzed to quantify the percent neuronal area in the ONL and INL.

### 4.7 DHE Labeling

Tissues were fluorescently labeled with DHE (Sigma–Aldrich #D7008) for 30 min at 37°C (10 µM final concentration in PBS) prior to mounting.

### 4.8 Microscopy and Quantitative IHC

Fluorescence and bright‐field images were acquired using a Carl Zeiss Axio Imager Z1 fluorescence microscope with ZEN 2.6 blue edition software (Carl Zeiss MicroImaging, Inc.) and equipped with ApoTome, AxioCam MRm, and AxioCam HRc cameras. Multi‐channel image acquisition was used to create images with several channels. Images were repeatedly captured at 20 × (resolution of 1388 × 1040 pixels, 447.63 µm × 335.30 µm per image) or 40 × (resolution of 1388 × 1040 pixels, 223.82 µm × 167.70 µm per image) at the same focal planes with the same exposure time for each marker and human donor.

For quantitative analyses, ten images per ST retina were consistently acquired from anatomically matched regions, including three central (C), four mid‐peripheral (M), and three far‐peripheral (F) retinal fields. Retinal thickness (µm) was measured manually using ZEN 2.6 software as the distance between the inner limiting membrane (ILM) and outer limiting membrane (OLM). Images were exported to NIH ImageJ2/Fiji (v2.14.0) for quantitative analysis. For each biomarker (VGLUT1, synaptophysin, PSD95, NMDAR2A, 12F4‐Aβ_42_, DHE‐ROS, p75NTR, UCHL1, pS396‐tau, IBA1, GFAP, and vimentin), single‐channel 20× fluorescence images (∼150 × 10^3^ µm^2^/image) were converted to grayscale and subjected to histogram‐based thresholding to establish a baseline intensity. The resulting threshold was then applied uniformly to all images for the corresponding biomarker across all diagnostic groups to ensure unbiased quantification. ImageJ2/Fiji particle analysis was subsequently used to quantify the total immunoreactive (IR) area (µm^2^) and percentage (%) IR area. Values from all sampled retinal regions were averaged to generate a single value per biomarker for each donor. To minimize anatomical variability, retinal biomarker quantification, including neurodegeneration measurements, glial cell markers, synaptic and proteinopathy markers, and retinal thickness assessments, was performed on the same images from precisely matched retinal subregions across all cases. All image acquisition, quantification, and statistical analyses were performed by investigators blinded to diagnostic group assignment.

### 4.9 TEM Analysis

Tissue samples were initially fixed in 4% paraformaldehyde (PFA) in phosphate‐buffered saline (PBS) for 20 min at room temperature (RT), followed by three washes in PBS (10 min each). For intracellular antigen labeling, samples were permeabilized and blocked in PBS containing 1% bovine serum albumin (BSA) and 0.1% Triton X‐100 for 15 min at RT, then rinsed in PBS. Primary antibodies against PSD95 and VGLUT1 (Table 5) were diluted in blocking solution (1% BSA in PBS) and applied overnight at 4°C in a humidified chamber. Samples were then washed three times with PBS (5 min each). Secondary detection was performed using species‐specific, gold‐conjugated secondary antibodies (anti‐rabbit, 6 nm; anti–guinea pig, 12 nm), diluted in PBS containing 1% BSA. Samples were incubated for 1 h at RT, followed by 3 additional PBS washes (5 min each). Subsequently, samples were post‐fixed in half‐strength Karnovsky's fixative (2% paraformaldehyde, 2.5% glutaraldehyde in 0.1 M cacodylate buffer, pH 7.2) for 15 min. Following post‐fixation, the samples were rinsed in 0.1 M cacodylate buffer (pH 7.2), then post‐fixed in 2% osmium tetroxide for 1 h, rinsed again in cacodylate buffer, and subsequently in sodium acetate. En bloc staining was performed using 1% aqueous uranyl acetate for 1 h, followed by a wash in sodium acetate. Samples were then dehydrated through a graded series of ethanol (50%, 70%, 85%, 95%, and 100%), followed by incubation in 1:1 and 1:2 mixtures of ethanol and propylene oxide (PO), and finally in pure PO. Tissues were infiltrated with PO/Eponate resin mixtures and embedded in 100% Eponate resin. Polymerization was carried out at 60°C for 48 h. Ultrathin sections (60 nm) were obtained using an ultramicrotome and collected on copper grids. Sections were imaged using a JEOL JEM‐2100 LaB6 transmission electron microscope operating at 120 kV (JEOL USA). Digital images were captured with an Orius SC1000B CCD camera (Gatan).

### 4.10 MS‐based Proteome Analysis

In this study, we performed secondary analyses on previously published retinal MS data [28] to identify DEPs associated with synaptic function and integrity. In brief, this analysis included the following steps: (1) preparation of retinal and brain homogenates; (2) tandem mass tag labeling; (3) nanoflow liquid chromatography electrospray ionization tandem MS; and (4) database searching, peptide quantification, and statistical analysis. Given the exploratory nature and limited sample size of this human retinal dataset, we adopted a less stringent threshold, defining DEPs as proteins with an FDR‐adjusted *p* < 0.20 and |FC| > 1.2 (or, in selected sub‐analyses, an unadjusted *p* < 0.05 and |FC| > 1.2, as specified for each analysis) for downstream interpretation.

#### 4.10.1 Functional Network and Computational Analysis

Prediction of activation or inhibition of pathways related to retinal degeneration, synaptic function and integrity, and neurotransmission was analyzed with Ingenuity Pathway Analysis (IPA, Qiagen; https://digitalinsights.qiagen.com). Activation z‐scores, *p*‐values, and Benjamini‐Hochberg adjusted *p*‐values are reported. Volcano plots representing expression changes [log_2_(FC)] and significance levels [−log_10_(P)] in AD versus NC were created using Prism 10.3.1 (GraphPad) and included human proteins related to the synapse. The list of synaptic‐associated proteins was extracted from SynGO release 1.2 knowledge base. Gene Ontology (GO) analysis of synapse‐related differentially expressed proteins (DEPs) was performed in Metascape (https://metascape.org/; cutoffs: overlap ≥ 3, *p*‐value < 0.01, enrichment ≥ 1.5) and included the GO Biological Processes, Reactome, Kyoto Encyclopedia of Genes and Genomes (KEGG), and WikiPathways databases. Pathway networks were created in Metascape and subsequently loaded and modified in Cytoscape 3.10.2 (https://cytoscape.org/). Protein interaction networks were generated in String v12.0 and modified in Cytoscape. Heatmaps corresponding to the protein expression levels of select proteins in the retina of the 6 NC individuals and the 6 AD patients, standardized by unit variance scaling, were generated in ClustVis (https://biit.cs.ut.ee/clustvis/). To characterize the relationship between synaptic dysfunction and neuroinflammatory pathways within the retinal proteomic dataset, expression levels of key synaptic proteins (SYP, PSD95, and UCHL1) were correlated with a curated panel of inflammation‐related proteins. Candidate inflammatory markers were identified through an AI‐assisted literature review of the NCBI database and included proteins associated with microglial identity and activation, phagocytic and complement pathways, disease‐associated microglia (DAM), oxidative stress, cytokine signaling, astrocyte reactivity and homeostasis, blood–retinal barrier/vascular inflammation, and amyloidogenic processing. Correlation analyses were performed across the entire proteomic cohort irrespective of diagnostic group (NC or AD) to identify relationships between synaptic integrity and neuroinflammatory signaling. The MS‐based proteomics data have been deposited to the ProteomeXchange Consortium via the PRIDE [185] partner repository under the dataset identifier PXD040225 (https://www.ebi.ac.uk/pride/archive/projects/PXD040225; https://ftp.pride.ebi.ac.uk/pride/data/archive/2025/10/PXD040225).

#### 4.10.2 Generation of Gene‐Level Ranking Scores and GSEA

Differential protein abundance statistics were processed to generate a continuous, directionally signed ranking metric suitable for preranked GSEA [104]. For each quantified protein, the log fold‐change direction (positive or negative) and the raw *p*‐value from the comparison of interest were used to compute a score defined as: rank_score = sign(logFC) × [−log10(P)]. This metric integrates the magnitude and direction of differential abundance with the statistical strength of the evidence, producing a continuous distribution of values that preserves ordering across proteins. Proteins lacking a gene symbol or *p*‐value were removed. When multiple entries mapped to the same gene symbol, scores were averaged to produce a single representative value per gene. The resulting gene‐level scores were sorted in descending order to generate the ranked list required for preranked GSEA.

Pathway enrichment analysis was performed using the *gseapy* Python package (https://github.com/zqfang/gseapy), which implements the Broad Institute's GSEA algorithm. The prerank module was run with 1000 permutations, a minimum gene set size of 15, and a maximum gene set size of 500. Gene sets were obtained from the designated human pathway collections (e.g., KEGG_2021_Human, GO Biological Process, or WikiPathways, depending on the analysis). Normalized enrichment scores (NES), nominal *p*‐values, and FDR values were extracted from the gseapy output. Selected pathways are shown. Enrichment plots and ranked‐metric distributions were generated directly using gseapy's plotting utilities.

### 4.11 Biochemical Determination of Aβ1–42 Levels by Sandwich ELISA in Human Retina

In this study, we reused retinal Aβ_1–42_ data generated in our previous study [28] to perform pairwise correlation analyses with newly identified differentially expressed proteins. Fresh‐frozen human retinal strips from the temporal hemisphere were homogenized as previously described [27, 28]. The amount of Aβ_1–42_ in retinal homogenates was determined using an anti‐human Aβ_1–42_ end‐specific sandwich ELISA kit (Thermo Fisher, KHB3441) and normalized to the total protein concentration (Thermo Fisher Scientific).

### 4.12 WB Analysis of Human Retinal Proteins

Fresh‐frozen postmortem temporal hemiretina tissues from human donors were homogenized in radioimmunoprecipitation assay (RIPA) buffer (0.5 M Tris‐HCl, pH 7.4, 1.5 M NaCl, 2.5% deoxycholic acid, 10% NP‐40, 10 mM EDTA, Millipore; 20–188) supplemented with protease and phosphatase inhibitors. Protein concentration was determined using a bicinchoninic acid protein assay kit (Pierce). Lysates were cleared by brief centrifugation for 10 min at 8000 g, normalized, and boiled at 95°C after addition of 6X SDS loading dye. Equal amounts of protein (30 µg per sample) were separated on 4%–20% precast polyacrylamide gels (Bio‐Rad, catalog #4561094) and transferred to polyvinylidene difluoride membranes. The membranes were then blocked with 2.5% bovine serum albumin in 1x TBS‐T (Tris‐buffered saline with 0.1% Tween‐20) for 1 h at RT, followed by overnight incubation at 4 °C with the anti‐UCHL1, anti‐PSD95, anti‐synaptophysin, and anti‐p75NTR primary antibodies (Table 5). After washing with 1x TBS‐T, the membranes were incubated with fluorescently labeled secondary antibodies (Table 5), and bands were detected using the LI‐COR Odyssey imaging system. Band intensities were quantified using Image Studio software (LI‐COR), and relative protein expression levels were calculated by normalizing target protein signals to GAPDH. For the Western blot analyses shown in Figures 2I and 4D, SYP and UCHL1 were detected on the same membrane. After imaging, the membrane was stripped using 1× NewBlot Nitro Stripping Buffer (LI‐COR Biosciences, Cat. No. 928–40030) and reprobed for GAPDH, which served as the common loading control for both proteins. Densitometry was performed on the sequentially acquired immunoblots. Full, unmodified blots are provided in Figures S4 and S7E.

### 4.13 Preparation of Amyloid‐β_1–42_ Fibrils

Aβ_1–42_ fibrils (fAβ_42_; Anaspec #20276) were prepared as previously described [186] from lyophilized peptide that was first monomerized in ice‐cold hexafluoroisopropanol (HFIP, Sigma #52512) to a final concentration of 1 mM, aliquoted and dried to form a peptide film, which was stored at −20°C. To induce fibril formation, Aβ_1–42_ films were resuspended in media with 10% sterile NaOH 60 mM, vortexed for 30 s, then mixed with 45% sterile H_2_O and 45% sterile solution of 20 mM NaH2PO4+Na2HPO4 (pH 7.4). The final solution was vortexed again and incubated at 37°C for 2 weeks with shaking. Pre‐formed fAβ_42_ were sonicated for 60 s and diluted to the desired concentration using culture medium prior to assays.

### 4.14 SH‐SY5Y Human Neuroblastoma Cell Culture

Human SH‐SY5Y neuroblastoma cells (ATCC, CRL‐2266) were used to examine the effect of amyloid‐β on synaptic markers, UCHL1 expression, and neurotoxicity. Cells were seeded in 6‐ or 24‐well or 60 mm tissue culture plates and maintained at 37°C in a humidified incubator with 5% CO2 and 95% air. Cells were cultured in basic growth medium, comprised of Dulbecco's‐modified Eagle's medium (DMEM; Gibco, #11‐320‐033) supplemented with 10% fetal bovine serum (FBS), 1% penicillin–streptomycin (100 U/mL each, Gibco, #15140122), and 1% sodium pyruvate. Upon reaching approximately 80% confluence, cells were treated with 50 or 250 nM fAβ_42_ for 24‐, 48‐, or 72 h prior to processing for immunocytochemistry, Western blot, or quantitative PCR (described below). To further induce neuronal differentiation, cells were allowed to reach approximately 40% confluence and were subsequently cultured in differentiation medium consisting of DMEM, supplemented with 10 µM all‐trans retinoic acid (RA; Fisher Scientific, #AA4454002), and progressively reduced concentrations of FBS (5%, 2.5%, and 1%) for 5 to 8 days. Differentiation medium was replenished every 2 days. Following differentiation, cells were exposed to ∼250 nM fAβ_42_ for 48 h or 72 h. At the end of the treatment period, cells were processed for downstream analyses.

#### 4.14.1 WB Analysis of SH‐SY5Y Cells

SH‐SY5Y cells (1.5×10^6^ cells per well in six‐well plates, *n* = 2–3 replicates/group; or 4 × 10^5^ cells per 60‐mm dish, *n* = 3 replicates/group) were lysed in RIPA buffer supplemented with protease and phosphatase inhibitors. Protein concentrations were determined using a bicinchoninic acid (BCA) assay (Pierce). Equal amounts of protein (30 µg) were resolved on 4%–20% precast polyacrylamide gels (Bio‐Rad #4561094) and transferred to polyvinylidene fluoride membranes (PVDF). Membranes were blocked with 2.5% bovine serum albumin and incubated overnight at 4°C with primary antibodies (Table 5). Following primary incubation, membranes were probed with appropriate fluorescently labeled secondary antibodies (Table 5). Protein bands were visualized and quantified using Image Studio software (LI‐COR). Relative protein expression levels were calculated by normalizing target protein signals to GAPDH. For the Western blot analyses shown in Figure 5I,J, NeuN and SYP were detected on the same membrane. After imaging, the membrane was stripped using 1× NewBlot Nitro Stripping Buffer (LI‐COR Biosciences, Cat. No. 928–40030) and reprobed for GAPDH, which served as the common loading control for both proteins. Densitometry was performed on the sequentially acquired immunoblots. Full, unmodified blots are provided in Figure S9J.

#### 4.14.2 Immunocytochemistry

SH‐SY5Y cells grown on gelatin‐coated glass coverslips (undifferentiated: 1 × 10^5^ cells per well in 24‐well plates; differentiated: 2 × 10^4^ cells/well; three replicates per condition) were fixed with cold methanol, washed with PBS, and blocked using DAKO protein‐block. Cells were incubated with primary antibodies for 1 h, followed by same‐day incubation with the appropriate secondary antibodies for 1 h (Table 5), and then mounted. Fluorescence images were acquired using a Carl Zeiss Axio Imager Z1 fluorescence microscope at 20× and 40× magnification. For each condition, 8–10 fields of view were imaged. Image processing and quantification of immunoreactive areas were performed for each individual channel, converted to grayscale using NIH ImageJ2/Fiji (v2.14.0), as described above.

For immunocytochemistry, each experimental condition was performed using three independent biological replicates (separate culture wells). Images from all biological replicates were acquired using identical microscope settings. Individual neurons were manually delineated using the freehand selection tool in NIH ImageJ2/Fiji (v2.14.0). Following histogram‐based thresholding standardized for each biomarker, the percentage (%) IR area was quantified separately for each cell.

For data visualization, measurements from individual cells (white circles) were plotted to illustrate the distribution of cellular responses across independent biological replicates (culture wells). For statistical analysis, the mean of all analyzed cells within each biological replicate was calculated, with each well representing the experimental unit (red circles). Statistical analyses were performed exclusively on these biological replicate means and are presented as mean ± SEM (*n* = 3 wells per group). Throughout image acquisition, cell selection, and image analysis, investigators remained blinded to the experimental groups.

#### 4.14.3 Real‐Time PCR Analysis of mRNA Levels in SH‐SY5Y Cells

Total RNA was extracted from SH‐SY5Y cell pellets (4×10^5^ cells/dish, *n* = 3–4/group) using TRIzol Reagent (Invitrogen; 15596018). One microgram of total RNA was reverse transcribed (RT) to synthesize complementary DNA (cDNA) using the RevertAid RT kit (ThermoScientific; #K1691), which includes 5× reverse transcription buffer, 10 mM dNTP mix, RiboLock RNase Inhibitor (20 U/µL), random hexamer primers, and reverse transcriptase. Samples were incubated for 5 min at 25°C, followed by 60 min at 42°C using a thermal cycler (Applied Biosystems). Quantitative PCR was performed using gene‐specific primers: *SYP (*forward: 5′‐TTGGCTTCGTGAAGGTGCTGCA‐3′; reverse: 5′‐ACTCTCCGTCTTGTTGGCACAC‐3′), beta III Tubulin [*TUBB3*] (forward: 5′‐TCAGCGTCTACTACAACGAGGC‐3′; reverse: 5′ GCCTGAAGAGATGTCCAAAGGC‐3′), *UCHL1* (forward: 5′‐CAGTTCAGAGGACACCCTGCTG‐3′; reverse: 5′‐CCACAGAGCATTAGGCTGCCTT‐3′), and *GAPDH* (forward: 5′‐ACCACAGTCCATGCCATCAC‐3′; reverse: 5′‐TCCACCACCCTGTTGCTGTA‐3*′*) with Power‐Up‐SYBR Green Master Mix (Applied Biosystems; A25742) on a CFX96 Real‐Time System (BioRad). Expression levels of *SYP, TUBB3, UCHL1*, and *GAPDH* mRNA were quantified using the relative threshold ^ΔΔ^Ct method: ^ΔΔ^Ct  =  (primer efficiency)^(−^ΔΔ^Ct), where ^ΔΔ^Ct means ΔCt (target gene) − ΔCt (reference gene, GAPDH).

### 4.15 Primary Mouse Hippocampal Neuronal Culture

All animal procedures were approved by the Institutional Animal Care and Use Committee (IACUC#9589) of Cedars‐Sinai Medical Center and performed in accordance with relevant guidelines and regulations. Hippocampi were dissected from postnatal day 0 (P0) C57BL/6 mice (The Jackson Laboratory, Strain #:000664) of both sexes in Hank's Balanced Salt Solution containing 10 mM HEPES and 20 mM glucose. Tissue was digested in 0.25% trypsin and washed three times with Minimal Essential Medium (Gibco, 11090‐081) supplemented with 1x B27 Plus (GIBCO, A3582801), 2 mM Glutamax (GIBCO, 35050–061), 5% FBS (HyClone, defined), 21 mM glucose (Sigma, G8769) and 1x penicillin/streptomycin (Corning, 30‐002‐CL). Tissue was dissociated by trituration, and cells were plated on poly‐L‐lysine (Sigma, A005C)‐coated coverslips (ICC) at a density of 2×10^5^ in the same medium or 2.5 × 10^5^ without cover slip (WB). After one day in vitro (DIV1), 3/4 of the medium was changed to Neurobasal Plus (GIBCO, A35829‐01) supplemented with B27, Glutamax, and penicillin/streptomycin. Upon maturation (DIV14), cells were treated with 250 nM fAβ_42_ for 24 or 48 h. Immunohistochemistry and Western Blot analyses were performed as described in Sections 4.14.1 and 4.14.2 above. For Western blots shown in Figure 5P, UCHL1 was detected first, after which the membrane was stripped (1× NewBlot Nitro Stripping Buffer) and sequentially reprobed for NeuN and SYP. Following imaging, the membrane was stripped again and reprobed for GAPDH, which served as the common loading control for all target proteins analyzed on the same membrane. Densitometry was performed from the sequentially acquired immunoblots. Full, unmodified blots are provided in Figure S10C.

### 4.16 Machine Learning Prediction

The dataset was split into training (80%, 38/14/44 NC/MCI/AD) and test sets (20%, 10/4/10 NC/MCI/AD), stratified by clinical diagnosis and patient sex. Two random forest regression models were trained to predict Braak stage and MMSE using 80 estimators and L1 loss, with both models using the same set of retinal biomarkers for their predictions. Due to random forest models being sensitive to their initial conditions, we instantiated the models using 1,000 different random seeds to identify which features were robustly predictive of their target based on their SHAP values [187]. At each iteration, the top features sorted by mean absolute SHAP were tabulated. After all iterations, these features were gathered, and the fraction of iterations in which they were present was computed (1.0 signifies the feature was present in all iterations; 0.5, in half). Learning curves were produced using 10 steps between 10% and 100% of data; cross‐validation scores were obtained using five‐fold cross‐validation and taking the mean across folds.

### 4.17 Statistical Analysis

All statistical analyses were conducted using GraphPad Prism 10.0.3 (GraphPad Software). Data are presented as mean ± standard error of the mean (SEM) for bar graphs. Violin plots display the median together with the lower and upper quartiles. The number of samples examined for each group is specified for each set of data in the corresponding figure caption. Comparisons among three or more groups were performed using one‐way or two‐way analysis of variance (ANOVA), followed by Tukey's or Šidák's post hoc test for multiple comparisons. Comparisons between two independent groups were assessed using two‐sided unpaired *t*‐tests. Associations between continuous variables were evaluated using the Pearson correlation coefficient (*r_p_*) for parametric data or Spearman rank correlation coefficient (*r_s_*) for non‐parametric data. Pairwise correlation coefficients *r_p_ or r_s_* and *p* values were reported to indicate the direction, strength, and significance of the linear relationship. For multivariable correlation analyses, P values were adjusted for multiple comparisons using the Holm–Šídák method, and P*_adj._* are reported. A *p* value of <0.05 was considered statistically significant. Where applicable, fold changes (FC) and corresponding 95% confidence intervals (CI) were calculated to quantify the magnitude and precision of observed effects.

## Author Contributions

A.R. and M.K.H.: Conceptualization; A.R., J‐P.V., A.H., Y.K., S.S., E.R., E.B., An.R., O.J., J.S., B.P.G., B.G., D.H., L.S.S, K.S., J.G.M, D‐T.F., and M.K.H.: Methodology; A.R., J‐P.V., A.H., Y.K., S.S., E.R., E.B., An.R., O.J., J.S., L.S., D.H, L.S.S, M.M., J.G.M, D‐T.F., and M.K.H.: Formal analysis and data curation; A.R., J‐P.V., B.P.G., D‐T.F., and M.K.H.: Writing – original draft: All authors: Writing – review and editing; A.R., J‐P.V., A.H., Y.K., S.S., J.G.M., D‐T. F., and M.K.H.: Visualization; M.K.H.: Supervision; M.K.H. and K.L.B. Funding acquisition.

## Conflicts of Interest

Y.K., K.L.B., and M.K.H. are co‐founding members of Neurovision Imaging, Inc., Sacramento, CA, USA. All other authors declare no conflicts of interest related to this work.

## Supporting information




**Supporting File**: advs77264‐sup‐0001‐SuppMat.pdf.

## Data Availability

Mass spectrometry data are publicly available via the PRIDE Database under the ProteomeXchange accession number PXD040225. All data are available in the main text, tables, and figures or in the supplementary materials (Figures S1–S12 and Tables S1–S7). All other material or data requests should be addressed to the corresponding author. The Jupyter Notebook containing the analysis with random forests has been uploaded to https://Github.com/Xomicsdatascience/Ubiquitin_Ad. Data will become publicly available as of the date of publication.
