# Supplementary material for: Early Retinal UCHL1 Dysregulation Coupled With Synaptic Loss Reflects Alzheimer's Disease Severity
Source: Adv Sci (Weinh). 2026 Aug 29;13(63):e00020. doi: 10.1002/advs.202600020 (PMC13525425; doi:10.1002/advs.202600020)
Supplement: Supplementary file 1 [file ADVS-13-e00020-s001.pdf]

Supplementary Online Content

Early Retinal UCHL1 Dysregulation Coupled with Synaptic Loss Reflects Alzheimer's Disease Severity

Altan Rentsendorj, Jean-Philippe Vit, Alexandre Hutton, Yosef Koronyo, Saba Shahin, Edward Robinson, Ernesto Barron, Anthony Rodriguez, Ousman Jallow, Julia Sheyn, Bhakta Prasad Gaire, Lalita Subedi, Miyah Rene Davis, Barathan Gnanabharathi, Alexander V. Ljubimov, Stuart L. Graham, Vivek K. Gupta, Mehdi Mirzaei, Debra Hawes, Katlin Silm, Keith L. Black, Lon S. Schneider, Alfredo A. Sadun, Jesse G. Meyer, Dieu-Trang Fuchs, Maya Koronyo-Hamaoui

Corresponding author: Maya Koronyo-Hamaoui, PhD, maya.koronyo@csmc.edu

Table S1. List of human donors in this study.
Table S2. Stratification of retinal synaptic markers by CDR.
Table S3. Stratification of retinal synaptic markers by Braak stage.
Table S4. Stratification of retinal synaptic markers by MMSE score.
Table S5. Correlation between retinal synaptic markers and brain pathology.
Table S6. Synapse-associated DEPs upregulated in AD versus NC.
Table S7. Synapse-associated DEPs downregulated in AD versus NC.

Figure S1. Pre- and post-synaptic density in retinal sub-regions from MCI and AD patients and relations to retinal amyloidosis and tauopathy.
Figure S2. Gene set enrichment analyses of AD retina mass spectrometry data.
Figure S3. P75NTR and photoreceptor degeneration pathway in the AD retina.
Figure S4. Full western blots of PSD95, Synaptophysin and p75NTR.
Figure S5. Regional and spatial retinal atrophy assessed by retinal thickness.
Figure S6. Extended analyses on retinal ROS and p75NTR.
Figure S7. Extended data on retinal UCHL1.
Figure S8. UCHL1 and associated molecular pathways in the AD retina.
Figure S9. Extended data on SH-SY5Y cells.
Figure S10. Extended data on mouse primary hippocampal neurons.
Figure S11. Extended data on synaptic alterations link to retinal p75NTR and retinal neuroinflammation.
Figure S12. Extended data on machine learning prediction of AD severity.

Table S1. List of postmortem human retinas and brains used in this study.

Diagnosis	Age at death	Thal A	Braak B	CERAD C	CAA score	Co-morbid (LB/AS/VD)	Braak stage	CDR score	MMSE score	Study type
NC1	86	n.a.	n.a.	n.a.	n.a.	n.a.	n.a.	n.a.	n.a.	Hist
NC2	75	n.a.	n.a.	n.a.	n.a.	n.a.	n.a.	n.a.	n.a.	Hist
NC3	76	n.a.	n.a.	n.a.	n.a.	n.a.	n.a.	n.a.	n.a.	Hist
NC4	73	n.a.	n.a.	n.a.	n.a.	n.a.	n.a.	n.a.	n.a.	Hist
NC5	85	2	1	2	0	−/+	I-II	0	30	Hist
NC6	80	n.a.	n.a.	n.a.	n.a.	n.a.	n.a.	n.a.	n.a.	Hist
NC7	84	n.a.	n.a.	n.a.	n.a.	n.a.	n.a.	n.a.	30	Hist
NC8	81	3	1	2	0.25	−/+	I-II	0	23	Hist
NC9	95	3	3	2	1	−/+	V	0	30	Hist
NC10	69	0	0	1	0	−/+	0	1	28	Hist
NC11	88	2	1	2	0	−/+	I	0	30	Hist
NC12	77	n.a.	n.a.	n.a.	n.a.	n.a.	n.a.	n.a.	30	Hist
NC13	70	n.a.	n.a.	n.a.	n.a.	n.a.	n.a.	n.a.	30	Hist
NC14	87	n.a.	n.a.	n.a.	n.a.	n.a.	n.a.	n.a.	30	Hist
NC15	58	n.a.	n.a.	n.a.	n.a.	n.a.	n.a.	n.a.	30	Hist
NC16	92	1	1	0	0.5	−/+	I	n.a.	25	Hist
NC17	95	1	1	1	0	n.a.	I	0	30	Hist
NC18	76	2	0	2	0	n.a.	0	0	29	Hist
NC19	95	1	0	0	0.5	−/+	I-II	0	30	Hist
NC20	99	1	2	1	0	n.a.	III	0	29	Hist
NC21	93	3	2	3	0.5	−/+	III-IV	1	27	Hist
MCI1	93	2	0	2	0	−/+	0	3	19	Hist
MCI2	86	3	1	3	0	−/+	I-II	2	15	Hist
MCI3	94	2	1	2	0	n.a.	I-II	0.5	29	Hist
MCI4	88	1	2	2	0	−/+	III	3	n.a.	Hist
MCI5	93	3	2	2	2	−/+	IV	3	11	Hist
MCI6	98	2	3	2	2	−/+	V	2	15	Hist
MCI7	83	2	3	2	1.5	−/+	III-IV	0	26	Hist
MCI8	91	2	2	2	0	−/+	III	3	29	Hist
MCI9	87	3	3	3	1.5	−/+	V-VI	3	13	Hist
MCI10	89	1	2	2	1	n.a.	III-IV	0.5	24	Hist
MCI11	80	3	3	2	1	−/+	V	3	29	Hist
AD1	76	3	3	3	2	−/+	V	1	26	Hist
AD2	90	1	3	3	1	−/+	V	3	9	Hist
AD3	93	3	2	2	0	−/+	III-IV	3	20	Hist

Diagnosis	Age at death	Thal A	Braak B	CERAD C	CAA score	Co-morbid (LB/AS/VD)	Braak stage	CDR score	MMSE score	Study type
AD4	86	3	3	2	1	−/+	V-VI	3	18	Hist
AD5	88	3	3	3	1.5	−/+	V-VI	1	16	Hist
AD6	93	2	2	2	1.5	−/+	III-IV	3	17	Hist
AD7	90	3	2	3	1	−/+	IV	3	n.a.	Hist
AD8	88	2	3	3	1.5	−/+	V	3	4	Hist
AD9	100	2	3	3	1	−/+	V-VI	2	16	Hist
AD10	88	2	3	2	1	−/+	V-VI	1	4	Hist
AD11	90	3	3	3	0	−/+	V-VI	n.a.	n.a	Hist
AD12	81	3	3	3	2	−/+	VI	n.a.	4	Hist
AD13	83	3	2	3	3	−/+	IV	1	18	Hist
AD14	89	3	3	3	2	−/+	V-VI	n.a.	6	Hist
AD15	75	3	3	3	0	−/+	III	n.a.	4	Hist
AD16	71	3	3	3	2	−/+	III	n.a.	10	Hist
AD17	64	3	3	3	0	−/+	III	3	5	Hist
AD18	90	3	3	2	2	−/+	III	2	18	Hist
AD19	97	3	3	3	0	−/+	III	n.a.	4	Hist
AD20	97	3	2	3	1	−/+	III	1	26	Hist
AD21	81	3	3	3	1.5	−/+	III	3	12	Hist
AD1	48	3	3	3	2	−/−	V-VI	n.a.	n.a.	Proteins
AD2	93	2	3	3	1	−/+	V	3	n.a.	Proteins
AD3	88	2	3	2	1	−/+	V-VI	1	4	Proteins
AD4	88	1	2	2	0	−/+	III	3	n.a.	Proteins
AD5	94	3	3	3	0	−/+	V-VI	3	n.a.	Proteins
AD6	100	2	3	3	0.5	−/+	V-VI	2	16	Proteins
AD7	81	3	3	3	0	−/+	V-VI	n.a.	12	Proteins
AD8	78	n.a.	n.a.	n.a.	n.a.	n.a.	n.a.	n.a.	n.a.	Proteins
AD9	74	n.a.	n.a.	n.a.	n.a.	n.a.	n.a.	n.a.	n.a.	Proteins
NC1	75	n.a.	n.a.	n.a.	n.a.	n.a.	n.a.	n.a.	n.a.	Proteins
NC2	72	n.a.	n.a.	n.a.	n.a.	n.a.	n.a.	n.a.	n.a.	Proteins
NC3	81	3	1	2	0.25	−/−	I-II	0	23	Proteins
NC4	69	n.a.	n.a.	n.a.	n.a.	n.a.	n.a.	n.a.	n.a.	Proteins
NC5	79	n.a.	n.a.	n.a.	n.a.	n.a.	n.a.	n.a.	n.a.	Proteins
NC6	85	0	1	3	1	−/+	I-II	0.5	n.a.	Proteins
NC7	76	n.a.	n.a.	n.a.	n.a.	n.a.	n.a.	n.a.	n.a.	Proteins

AD, Alzheimer's disease dementia; MCI, mild cognitive impairment; NC, normal cognition; F, female; M, male; A, Asian; B, Black; H, Hispanic; W, White; Hist, Histology; A, Aβ plaque score modified from Thal; B, NFT stage modified from Braak; C, Neuritic plaque score modified from CERAD; CAA, Cerebral amyloid angiopathy; LB, Lewy bodies; ASVD. Atherosclerosis; CDR, Clinical dementia rating; MMSE, Mini-Mental State Examination; n.a., not available; +: present; –: none; APOE, apolipoprotein alleles.

Table S2. Stratification of retinal synaptic markers by CDR score.

Retinal	CDR=0-0.5			CDR=1-2			CDR=3			CDR 0-0.5 vs 1-2		CDR 1-2 vs 3		CDR 0-0.5 vs 3	
	Mean	SD	n	Mean	SD	n	Mean	SD	n	P	FC [95% CI]	P	FC [95% CI]	P	FC [95% CI]
VGLUT1	36.3	9.7	9	22.2	10.3	10	19.3	5.6	11	<0.0001	-1.6 [-2.18, -1.09]	0.52	-1.1 [-1.53, -0.77]	<0.0001	-1.9 [-2.34, -1.42]
SYP	19.6	7.2	5	9.1	4.0	5	6.4	3.0	8	0.019	-2.2 [-3.24, -1.07]	0.71	-1.4 [-2.15, -0.71]	0.0006	-3.1 [-4.48, -1.68]
PSD95	14.0	4.9	9	9.5	5.7	12	7.3	1.8	13	0.21	-1.5 [-2.08, -0.87]	0.63	-1.3 [-1.79, -0.83]	0.0304	-1.9 [-2.44, -1.42]
NMDAR2A	17.6	8.1	7	7.5	2.7	6	7.0	2.2	11	0.0092	-2.4 [-3.41, -1.29]	0.99	-1.1 [-1.44, 10.70]	0.0013	-2.5 [-3.50, -1.54]

P < 0.05 value determines the statistical significance by two-way ANOVA and Tukey's post-hoc multiple comparison test. CDR, Clinical dementia rating; CI, Confidence interval; FC, Fold change; n, sample size; SD, Standard deviation

Table S3. Stratification of retinal synaptic markers by Braak stage.

Retinal	Braak=0-II			Braak=III-IV			Braak=V-VI			Braak 0-II vs III-IV		Braak III-IV vs V-VI		Braak 0-II vs V-VI	
	Mean	SD	n	Mean	SD	n	Mean	SD	n	P	FC [95% CI]	P	FC [95% CI]	P	FC [95% CI]
VGLUT1	34.8	10.1	8	23.0	10.9	11	20.6	7.8	13	0.0006	-1.5 [-2.04, -0.99]	0.67	-1.1 [-1.50, -0.73]	<0.0001	-1.7 [-2.17, -1.20]
SYP	17.2	5.8	5	6.3	3.1	4	8.6	6.8	11	0.042	-2.7 [-4.26, -1.19]	0.82	-0.7 [-1.22, -0.24]	0.049	-2.0 [-3.09, -0.90]
PSD95	14.7	5.1	8	9.1	5.1	12	7.0	3.0	19	0.16	-1.6 [-2.25, -0.97]	0.66	-1.3 [-1.78, -0.82]	0.019	-2.1 [-2.74, -1.45]
NMDAR2A	15.8	9.4	7	8.0	2.4	7	7.2	4.0	12	0.074	-2.0 [-2.96, -1.00]	0.97	-1.1 [-1.54, -0.68]	0.02	-2.2 [-3.39. -1.01]

P < 0.05 value determines the statistical significance by two-way ANOVA and Tukey's post-hoc multiple comparison test. CI, Confidence interval; FC, Fold change; n, sample size; SD, Standard deviation.

Table S4. Stratification of retinal synaptic markers by MMSE score.

**Retinal**	**MMSE>26**			**MMSE≤26**			**2way ANOVA Multiple comparisons**	
	Mean	SD	n	Mean	SD	n	P value	FC [95% CI]
VGLUT1	41.8	7.1	14	20.0	6.1	22	<0.0001	-2.1 [-2.42, -1.77]
SYP	24.8	8.0	9	8.2	5.0	14	<0.0001	-3.0 [-4.16, -1.87]
PSD95	16.2	4.5	16	7.0	2.6	26	<0.0001	-2.3 [-2.76, -1.85]
NMDAR2A	19.2	6.9	12	7.6	3.4	18	<0.0001	-2.5 [-3.27, -1.79]

P < 0.05 value determines the statistical significance by two-way ANOVA with Šídák's post-hoc multiple comparison test. CI, Confidence interval; FC, Fold change; MMSE, Mini-Mental State Examination, n, sample size; SD, Standard deviation.

Table S5. Correlation between retinal synaptic markers and brain pathology.

Brain / Retina	**Aβ plaques (severity score)**	**NFTs (severity score)**
VGLUT1	−0.071<br>32	−0.38<br>32
SYP	−0.47*<br>20	−0.65**<br>20
PSD95	−0.34*<br>39	−0.45**<br>39
NMDAR2A	−0.42*<br>26	−0.48*<br>26

Pairwise Spearman rank correlation analyses showing the strength of the association (*r*, upper value), sample size number (n, lower value), and adjusted P values with Holm-Šídák multiple-comparison correction method (adjusted for each retinal synaptic marker against 2 brain pathology scores): *P < 0.05, **P < 0.01.

Table S6. Synapse-associated DEPs upregulated in AD versus NC.

Accession	Symbol	Description	NC	AD	FC	*P*	adj. *P*
O75487	GPC4	Glypican-4	20.9	39.1	1.87	0.0178	0.1898
P09497	CLTB	Clathrin light chain B	249.1	462.2	1.86	0.0036	0.1294
P62942	FKBP1A	Peptidyl-prolyl cis-trans isomerase FKBP1A	120.3	209.9	1.75	0.0047	0.1826
Q01469	FABP5	Fatty acid-binding protein 5	1176.3	2053.5	1.75	0.0126	0.1736
Q13542	EIF4EBP2	Eukaryotic translation initiation factor 4E-binding protein 2	11.3	18.9	1.67	0.0165	0.1857
P08138	NGFR	Tumor necrosis factor receptor superfamily member 16	24.7	40.4	1.64	0.0007	0.1203
Q6PUV4	CPLX2	Complexin-2	131.2	214.0	1.63	0.0177	0.1894
Q6UUV9	CRTC1	CREB-regulated transcription coactivator 1	54.5	83.5	1.53	0.0151	0.1826
P42574	CASP3	Caspase-3	24.0	35.4	1.47	0.0073	0.1496
Q13303	KCNAB2	Voltage-gated potassium channel subunit beta-2	83.9	122.1	1.46	0.0010	0.1203
P62330	ARF6	ADP-ribosylation factor 6	298.8	431.8	1.45	0.0031	0.1285
Q13526	PIN1	Peptidyl-prolyl cis-trans isomerase NIMA-interacting 1	719.4	1031.8	1.43	0.0106	0.1670
I3L504	EIF5A	Eukaryotic translation initiation factor 5A-1	956.2	1338.9	1.40	0.0211	0.1951
P18085	ARF4	ADP-ribosylation factor 4	645.8	883.5	1.37	0.0007	0.1203
P62166	NCS1	Neuronal calcium sensor 1	91.9	124.2	1.35	0.0090	0.1594
P23763	VAMP1	Vesicle-associated membrane protein 1	117.0	157.1	1.34	0.0172	0.1880
P46934	NEDD4	E3 ubiquitin-protein ligase NEDD4	14.4	19.3	1.34	0.0189	0.1939
P68032	ACTC1	Actin, alpha cardiac muscle 1	3608.7	4806.4	1.33	0.0138	0.1775
Q14643	ITPR1	Inositol 1,4,5-trisphosphate receptor type 1	10.7	14.2	1.33	0.0206	0.1944
H7C394	CAMK2B	Calcium/calmodulin-dependent protein kinase type II subunit beta	41.1	54.4	1.32	0.0060	0.1415
Q14012	CAMK1	Calcium/calmodulin-dependent protein kinase type 1	108.6	143.4	1.32	0.0003	0.1203
A0A0U1RRM6	ENAH	Protein enabled homolog	235.1	310.3	1.32	0.0044	0.1349
Q14515	SPARCL1	SPARC-like protein 1	420.5	552.9	1.31	0.0119	0.1702
E5RJR5	SKP1	S-phase kinase-associated protein 1	1575.9	2046.6	1.30	0.0131	0.1744
O00499	BIN1	Myc box-dependent-interacting protein 1	61.5	79.7	1.30	0.0056	0.1415
Q8WUY3	PRUNE2	Protein prune homolog 2	153.4	194.3	1.27	0.0153	0.1826
Q9UH65	SWAP70	Switch-associated protein 70	348.4	440.6	1.26	0.0015	0.1203
O43765	SGTA	Small glutamine-rich tetratricopeptide repeat-containing protein alpha	1074.9	1357.5	1.26	0.0191	0.1939
Q9UK22	FBXO2	F-box only protein 2	778.1	982.0	1.26	0.0030	0.1277
Q99961	SH3GL1	Endophilin-A2	453.7	565.3	1.25	0.0223	0.1966

Q96P47	AGAP3	Arf-GAP with GTPase, ANK repeat and PH domain-containing protein 3	144.6	179.4	1.24	0.0097	0.1653
Q16515	ASIC2	Acid-sensing ion channel 2	30.3	37.6	1.24	0.0172	0.1880
P45974	USP5	Ubiquitin carboxyl-terminal hydrolase 5	2076.7	2537.6	1.22	0.0093	0.1615
P40123	CAP2	Adenylyl cyclase-associated protein 2	692.6	842.2	1.22	0.0115	0.1699
Q9H1P3	OSBPL2	Oxysterol-binding protein-related protein 2	393.0	475.6	1.21	0.0016	0.1203
O15145	ARPC3	Actin-related protein 2/3 complex subunit 3	276.8	334.1	1.21	0.0216	0.1963
P31749	AKT1	RAC-alpha serine/threonine-protein kinase	420.3	506.9	1.21	0.0045	0.1349

NC (n = 6) and AD (n = 6) are expressed as geometric means.
FC > 1.2; FDR-adjusted (adj.) $P < 0.2$.
The list of synapse-associated proteins was extracted from SynGo release 1.2 knowledge base for synapse research[1].

Table S7. Synapse-associated DEPs downregulated in AD versus NC.

Accession	Symbol	Description	NC	AD	FC	*P*	adj. *P*
Q12791	KCNMA1	Calcium-activated potassium channel subunit alpha-1	125.6	69.8	-1.80	0.0187	0.1933
A0A286YEX2	SYT6	Synaptotagmin VI	44.4	28.6	-1.55	0.0088	0.1594
Q13557	CAMK2D	Calcium/calmodulin-dependent protein kinase type II subunit delta	116.0	75.3	-1.54	0.0090	0.1596
O14775	GNB5	Guanine nucleotide-binding protein subunit beta-5	1751.3	1144.8	-1.53	0.0012	0.1203
Q9NQC3	RTN4	Reticulon-4	430.9	290.9	-1.48	0.0076	0.1534
Q08828	ADCY1	Adenylate cyclase type 1	582.3	394.8	-1.47	0.0004	0.1203
Q9UI40	SLC24A2	Sodium/potassium/calcium exchanger 2	376.1	255.8	-1.47	0.0098	0.1654
P49207	RPL34	60S ribosomal protein L34	1669.6	1135.7	-1.47	0.0001	0.1203
P83731	RPL24	60S ribosomal protein L24	2353.3	1601.0	-1.47	0.0019	0.1209
P62913	RPL11	60S ribosomal protein L11	1643.0	1122.0	-1.46	0.0032	0.1285
P61353	RPL27	60S ribosomal protein L27	1992.6	1376.6	-1.45	0.0007	0.1203
P62280	RPS11	40S ribosomal protein S11	2800.1	1944.2	-1.44	0.0063	0.1427
P35268	RPL22	60S ribosomal protein L22	614.0	429.6	-1.43	0.0002	0.1203
Q00839	HNRNPU	Heterogeneous nuclear ribonucleoprotein U	4906.0	3442.1	-1.43	0.0057	0.1415
F8VVT9	AGAP2	Arf-GAP with GTPase, ANK repeat and PH domain-containing protein 2	24.5	17.5	-1.40	0.0116	0.1700
P61764	STXBP1	Syntaxin-binding protein 1	1670.3	1209.7	-1.38	0.0014	0.1203
P52272	HNRNPM	Heterogeneous nuclear ribonucleoprotein M	17928.5	13023.5	-1.38	0.0041	0.1323
P36578	RPL4	60S ribosomal protein L4	4413.8	3224.1	-1.37	0.0038	0.1297
P42766	RPL35	60S ribosomal protein L35	2123.7	1558.9	-1.36	0.0002	0.1203
Q14839	CHD4	Chromodomain-helicase-DNA-binding protein 4	4536.8	3338.6	-1.36	0.0029	0.1265
P62244	RPS15A	40S ribosomal protein S15a	1082.2	801.5	-1.35	0.0071	0.1485
Q14721	KCNB1	Potassium voltage-gated channel subfamily B member 1	763.1	568.7	-1.34	0.0010	0.1203
A0A0A0MRM8	MYO6	Unconventional myosin-VI	1382.2	1038.1	-1.33	0.0060	0.1415
Q9BUJ2	HNRNPUL1	Heterogeneous nuclear ribonucleoprotein U-like protein 1	3273.8	2473.3	-1.32	0.0022	0.1209
P42677	RPS27	40S ribosomal protein S27	683.1	516.6	-1.32	0.0061	0.1415
P27635	RPL10	60S ribosomal protein L10	1574.7	1193.9	-1.32	0.0049	0.1399
P62277	RPS13	40S ribosomal protein S13	2212.4	1696.7	-1.30	0.0037	0.1294
P62424	RPL7A	60S ribosomal protein L7a	2942.2	2269.0	-1.30	0.0050	0.1403
P61026	RAB10	Ras-related protein Rab-10	2012.1	1555.6	-1.29	0.0048	0.1385
Q8TCG5	CPT1C	Carnitine O-palmitoyltransferase 1, brain isoform	488.7	378.6	-1.29	0.0019	0.1209
P50914	RPL14	60S ribosomal protein L14	1194.2	925.5	-1.29	0.0150	0.1826

P40429	RPL13A	60S ribosomal protein L13a	2466.8	1913.8	-1.29	0.0032	0.1285
Q4R9M9	KIF1B	Kinesin family member 1Bbeta isoform II	982.7	764.0	-1.29	0.0021	0.1209
Q13151	HNRNPA0	Heterogeneous nuclear ribonucleoprotein A0	3023.5	2351.1	-1.29	0.0067	0.1448
Q8NBU5	ATAD1	ATPase family AAA domain-containing protein 1	1262.0	986.9	-1.28	0.0194	0.1939
P39023	RPL3	60S ribosomal protein L3	4503.3	3527.3	-1.28	0.0220	0.1966
Q15717	ELAVL1	ELAV-like protein 1	2854.3	2237.0	-1.28	0.0025	0.1248
P61247	RPS3A	40S ribosomal protein S3a	4305.6	3383.3	-1.27	0.0012	0.1203
Q9NVJ2	ARL8B	ADP-ribosylation factor-like protein 8B	1105.3	870.2	-1.27	0.0033	0.1287
O60506	SYNCRIP	Heterogeneous nuclear ribonucleoprotein Q	33.5	26.4	-1.27	0.0120	0.1702
P61313	RPL15	60S ribosomal protein L15	1127.9	890.0	-1.27	0.0043	0.1346
P62917	RPL8	60S ribosomal protein L8	2556.6	2031.3	-1.26	0.0174	0.1880
P15880	RPS2	40S ribosomal protein S2	3731.9	2971.8	-1.26	0.0130	0.1742
Q1KMD3	HNRNPUL2	Heterogeneous nuclear ribonucleoprotein U-like protein 2	5206.0	4147.4	-1.26	0.0048	0.1384
Q8TAC9	SCAMP5	Secretory carrier-associated membrane protein 5	2392.0	1915.4	-1.25	0.0156	0.1829
P29323	EPHB2	Ephrin type-B receptor 2	954.8	764.6	-1.25	0.0127	0.1736
Q06787	FMR1	Synaptic functional regulator FMR1	1013.6	811.9	-1.25	0.0045	0.1349
Q9BTU6	PI4K2A	Phosphatidylinositol 4-kinase type 2-alpha	510.9	410.7	-1.24	0.0132	0.1744
A0A087WWK8	IQSEC1	IQ motif and SEC7 domain-containing protein 1	2184.4	1761.3	-1.24	0.0195	0.1939
P46776	RPL27A	60S ribosomal protein L27a	1539.6	1243.1	-1.24	0.0225	0.1976
P62241	RPS8	40S ribosomal protein S8	2133.6	1725.3	-1.24	0.0052	0.1403
O75396	SEC22B	Vesicle-trafficking protein SEC22b	3207.5	2595.5	-1.24	0.0062	0.1423
Q9NP72	RAB18	Ras-related protein Rab-18	3017.4	2442.1	-1.24	0.0088	0.1594
P26373	RPL13	60S ribosomal protein L13	2465.5	2000.2	-1.23	0.0147	0.1805
O00592	PODXL	Podocalyxin	464.2	376.8	-1.23	0.0072	0.1485
P62701	RPS4X	40S ribosomal protein S4, X isoform	3977.3	3228.8	-1.23	0.0102	0.1655
Q9UQM7	CAMK2A	Calcium/calmodulin-dependent protein kinase type II subunit alpha	925.4	752.0	-1.23	0.0109	0.1672
P18124	RPL7	60S ribosomal protein L7	4105.9	3336.5	-1.23	0.0223	0.1966
P62888	RPL30	60S ribosomal protein L30	810.1	662.2	-1.22	0.0165	0.1857
Q02878	RPL6	60S ribosomal protein L6	3584.9	2934.1	-1.22	0.0103	0.1656
J3QQ67	RPL18	60S ribosomal protein L18 (Fragment)	1882.2	1543.3	-1.22	0.0224	0.1969
Q8NC96	NECAP1	Adaptin ear-binding coat-associated protein 1	1072.7	879.9	-1.22	0.0019	0.1209
P23246	SFPQ	Splicing factor, proline- and glutamine-rich	9991.3	8196.4	-1.22	0.0034	0.1290
Q96CW1	AP2M1	AP-2 complex subunit mu	4427.6	3634.0	-1.22	0.0227	0.1984
F8W059	UNC13A	Protein unc-13 homolog A	48.6	39.9	-1.22	0.0189	0.1939
M0R0P8	MYO9B	Unconventional myosin-IXb	42.7	35.1	-1.22	0.0013	0.1203

P48454	PPP3CC	Serine/threonine-protein phosphatase 2B catalytic subunit gamma	431.4	356.4	-1.21	0.0111	0.1678
P84098	RPL19	60S ribosomal protein L19	751.9	622.9	-1.21	0.0206	0.1944
P07948	LYN	Tyrosine-protein kinase Lyn	2270.9	1882.2	-1.21	0.0012	0.1203
P62829	RPL23	60S ribosomal protein L23	2161.3	1798.7	-1.20	0.0081	0.1547
P26232	CTNNA2	Catenin alpha-2	7712.4	6434.0	-1.20	0.0066	0.1447

NC (n = 6) and AD (n = 6) are expressed as geometric means.
|FC| > 1.2; FDR-adjusted (adj.) $P < 0.2$.
The list of synapse-associated proteins was extracted from SynGo release 1.2 knowledge base for synapse research[1].

Reference

1. Koopmans, F., van Nierop, P., Andres-Alonso, M., Byrnes, A., Cijsouw, T., Coba, M.P., Cornelisse, L.N., Farrell, R.J., Goldschmidt, H.L., Howrigan, D.P., et al. (2019). SynGO: An Evidence-Based, Expert-Curated Knowledge Base for the Synapse. Neuron *103*, 217-234 e214. 10.1016/j.neuron.2019.05.002.

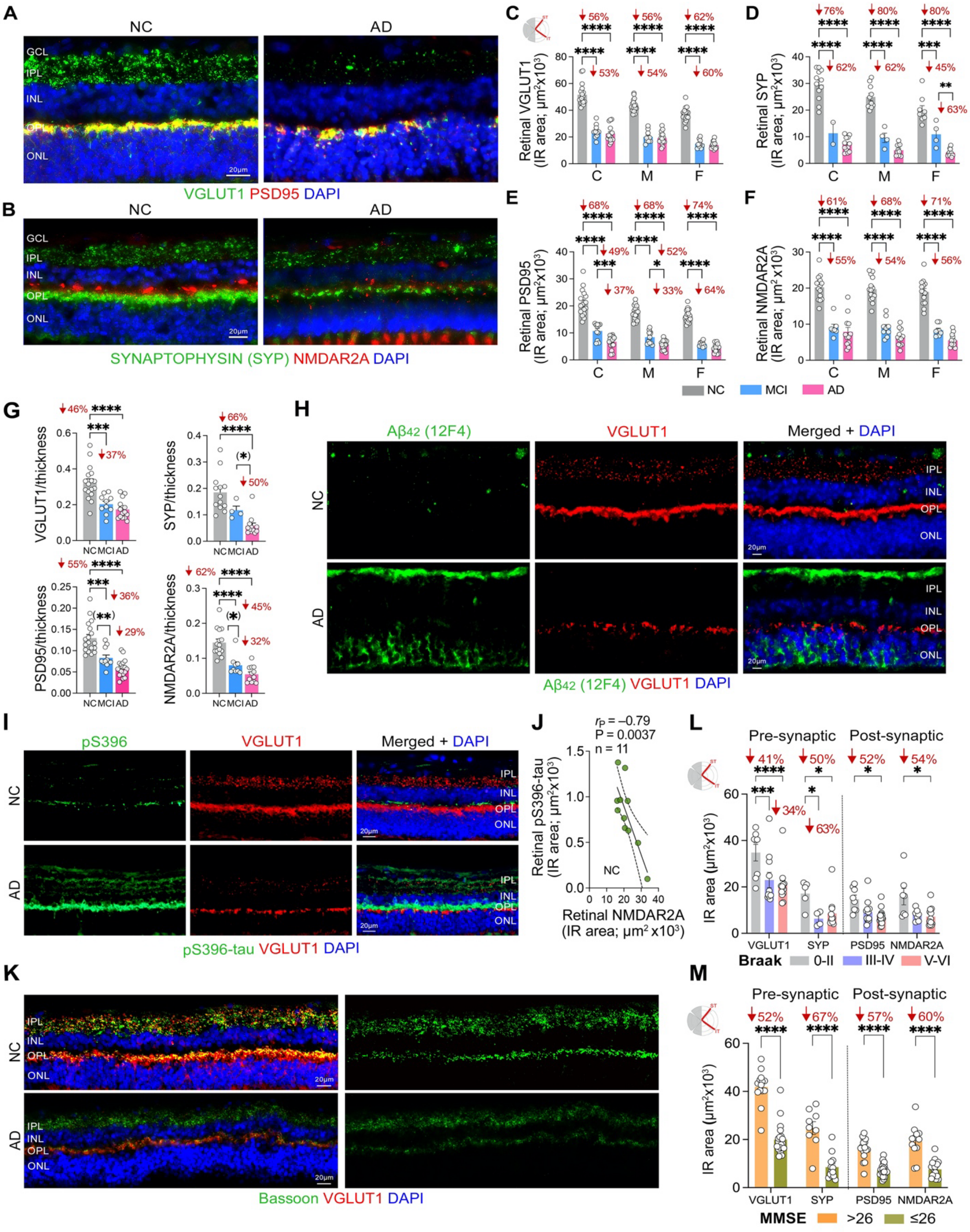
A
NC
AD
GCL
IPL
INL
OPL
ONL
20μm
VGLUT1 PSD95 DAPI
B
SYNAPTOPHYSIN (SYP) NMDAR2A DAPI
C
Retinal VGLUT1 (IR area; μm²x10³)
D
Retinal SYP (IR area; μm²x10³)
E
Retinal PSD95 (IR area; μm²x10³)
F
Retinal NMDAR2A (IR area; μm²x10³)
C
M
F
NC
MCI
AD
G
VGLUT1/thickness
SYP/thickness
PSD95/thickness
NMDAR2A/thickness
H
Aβ42 (12F4)
VGLUT1
Merged + DAPI
Aβ42 (12F4) VGLUT1 DAPI
I
pS396
VGLUT1
Merged + DAPI
pS396-tau VGLUT1 DAPI
J
rP = –0.79
P = 0.0037
n = 11
Retinal pS396-tau (IR area; μm²x10³)
Retinal NMDAR2A (IR area; μm² x10³)
L
Pre-synaptic
Post-synaptic
IR area (μm²x10³)
VGLUT1
SYP
PSD95
NMDAR2A
Braak
0-II
III-IV
V-VI
K
Bassoon VGLUT1 DAPI
M
MMSE
>26
≤26

Figure S1. Pre- and post-synaptic density in retinal sub-regions from MCI and AD patients and relations to retinal amyloidosis and tauopathy.

(**A**) Representative high-magnification fluorescence micrographs of retinal cross-sections immunolabeled for presynaptic VGLUT1 (green) and postsynaptic PSD95 (red) markers with nuclei (DAPI, blue) in a patient with AD versus NC control. Scale bars: 20 µm.

(**B**) Representative high-magnification fluorescence micrographs of retinal cross-sections immunolabeled for presynaptic Synaptophysin (SYP, green) and postsynaptic NMDAR2A (red) in a patient with AD versus NC control. Scale bars: 20 µm.

(**C-F**) Quantitative analyses of retinal VGLUT1 (**C**), SYP (**D**), PSD95 (**E**), and NMDAR2A (**F**) immunoreactive area (IR) in predefined retinal subregions, central (C), mid- (M), and far-periphery (F), in patients with MCI (due to AD; n = 2-11) and AD-dementia (n = 11-20) as compared with age and sex-matched NC controls (n = 9-18).

(**G**) Quantitative analyses of retinal synaptic markers IR area normalized to tissue thickness (µm) in patients with MCI (n = 4-11) and AD dementia (n = 12-18) versus NC controls (n = 13-18).

(**H**) Representative fluorescence micrographs of retinal cross-sections immunolabeled for 12F4-$A\beta_{42}$ (green)**,** VGLUT1 (red) and nuclei (DAPI, blue) in a patient with AD and in NC control. Scale bars: 20 µm.

(**I**) Representative fluorescence micrographs of retinal cross-sections immunolabeled for pS396-tau (green)**,** VGLUT1 (red) and nuclei (DAPI, blue) in a patient with AD versus NC control. Scale bars: 20 µm.

(**J**) Pearson's correlation (r_P) between retinal pS396 and NMDAR2A IR area.

(**K**) Representative fluorescence micrographs of retinal cross-sections immunolabeled for Bassoon (green), VGLUT1 (red) and nuclei (DAPI, blue). Scale bars: 20 µm.

(**L-M**). Quantitative analyses of retinal VGLUT1, SYP, PSD95 and NMDAR2A, when donors are stratified according to Braak stage [0-II = 5-8; III-IV = 4-12; V-VI = 11-19] (**L**) and 26-score cutoff on the mini-mental state examination [MMSE; >26 = 9-16; ≤26 = 14-26] (**M**).

Bar graphs display individual subjects (circles) as well as group means ± SEMs. *P < 0.05, **P < 0.01, ***P < 0.001, ****P < 0.0001, by two-sided unpaired t-test (in parenthesis), one-way or two-way ANOVA followed by Tukey's or Šídák's post hoc multiple comparison tests. Percentage (%) changes are shown in red. GCL, ganglion cell layer; IPL, inner plexiform layer; INL, inner nuclear layer; OPL, outer plexiform layer; ONL, outer nuclear layer.

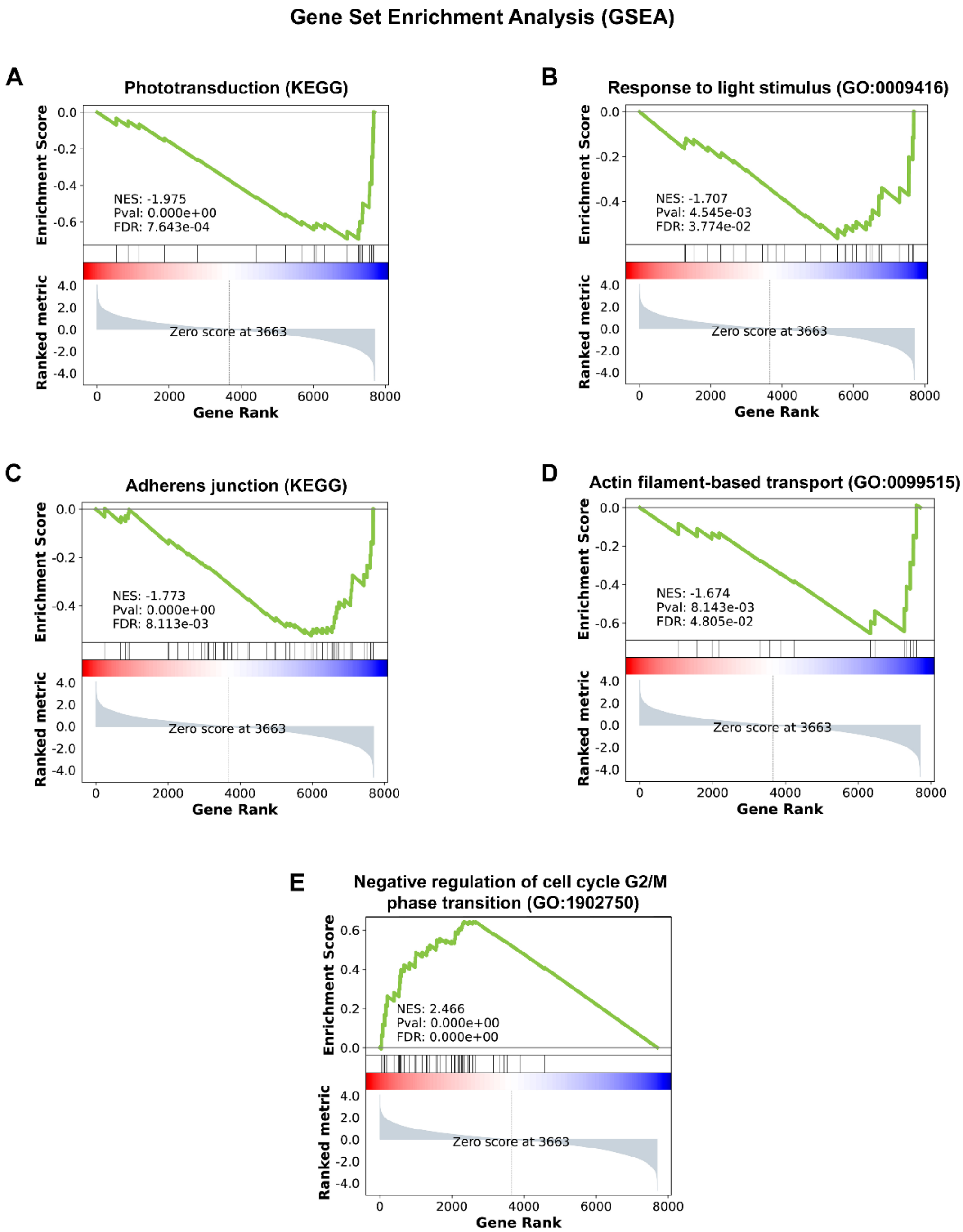

Figure S2. Gene set enrichment analyses (GSEA) of AD versus NC retina mass spectrometry data

(**A-B**) GSEA enrichment plots showing enrichment in the bottom-ranked dataset (downregulated in AD) for KEGG phototransduction (NES=-1.975, FDR<0.001) (**A**) and GO response to light stimulus (NES=-1.707, FDR<0.05) (**B**).

(**C-D**) GSEA enrichment plots showing enrichment in the bottom-ranked dataset (downregulated in AD) for KEGG adherens junction (NES=-1.773, FDR<0.01) (**C**) and GO actin filament-based transport (NES=-1.674, FDR<0.05) (**D**).

(**E**) GSEA enrichment plot for GO negative regulation of cell cycle G2/M phase transition enriched in our top-ranked dataset (upregulated in AD: NES=2=466, FDR<0.0001).

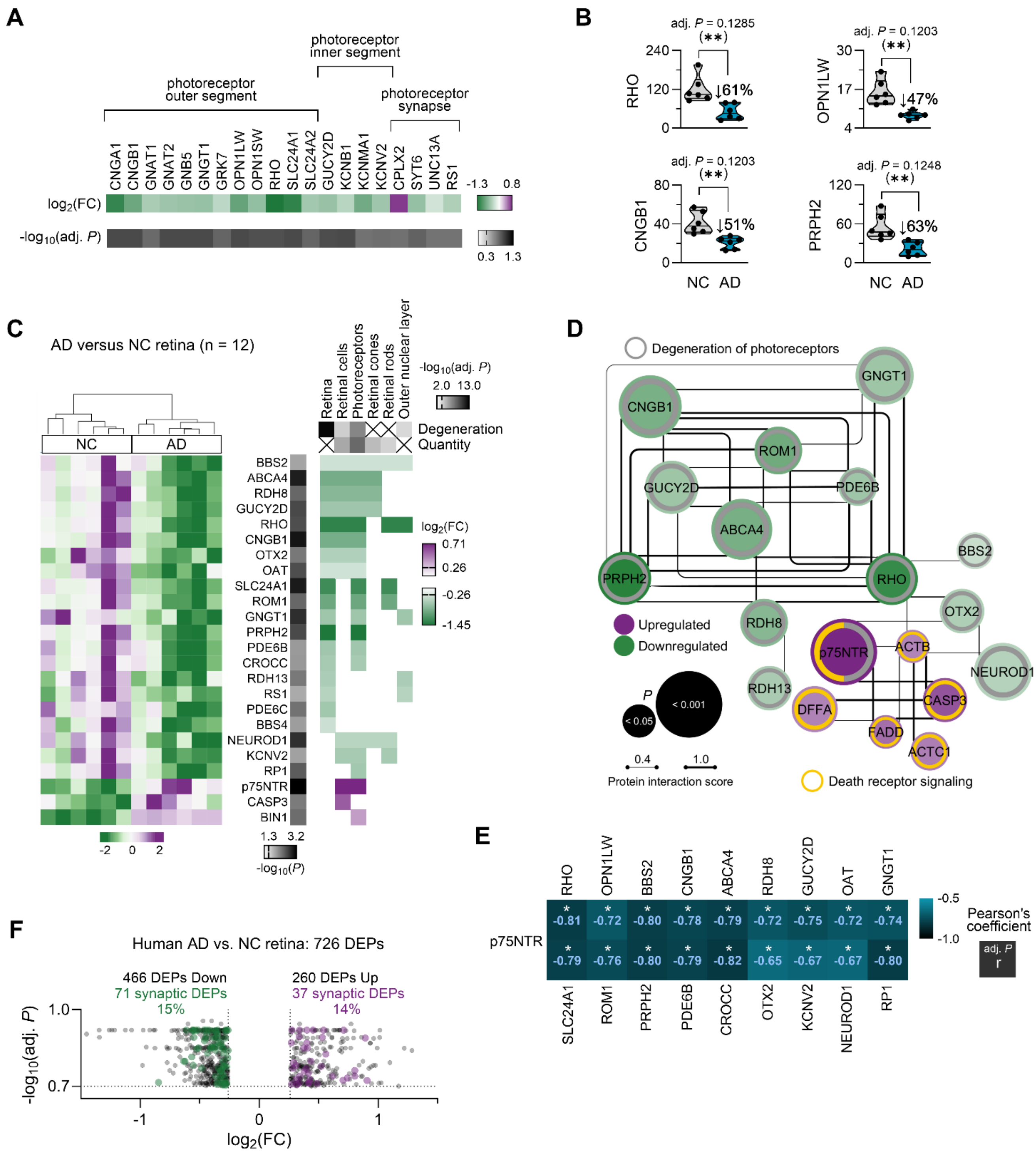
A
photoreceptor outer segment
photoreceptor inner segment
photoreceptor synapse
CNGA1 CNGB1 GNAT1 GNAT2 GNB5 GNGT1 GRK7 OPN1LW OPN1SW RHO SLC24A1 SLC24A2 GUCY2D KCNB1 KCNMA1 KCNV2 CPLX2 SYT6 UNC13A RS1
log2(FC)
-log10(adj. P)
B
adj. P = 0.1285
adj. P = 0.1203
adj. P = 0.1203
adj. P = 0.1248
RHO
OPN1LW
CNGB1
PRPH2
↓61%
↓47%
↓51%
↓63%
NC AD
C
AD versus NC retina (n = 12)
NC
AD
Retina
Retinal cells
Photoreceptors
Retinal cones
Retinal rods
Outer nuclear layer
Degeneration
Quantity
BBS2 ABCA4 RDH8 GUCY2D RHO CNGB1 OTX2 OAT SLC24A1 ROM1 GNGT1 PRPH2 PDE6B CROCC RDH13 RS1 PDE6C BBS4 NEUROD1 KCNV2 RP1 p75NTR CASP3 BIN1
log2(FC)
-log10(P)
D
Degeneration of photoreceptors
Upregulated
Downregulated
Protein interaction score
Death receptor signaling
E
p75NTR
Pearson's coefficient
adj. P
r
RHO OPN1LW BBS2 CNGB1 ABCA4 RDH8 GUCY2D OAT GNGT1
-0.81 -0.72 -0.80 -0.78 -0.79 -0.72 -0.75 -0.72 -0.74
-0.79 -0.76 -0.80 -0.79 -0.82 -0.65 -0.67 -0.67 -0.80
SLC24A1 ROM1 PRPH2 PDE6B CROCC OTX2 KCNV2 NEUROD1 RP1
F
Human AD vs. NC retina: 726 DEPs
466 DEPs Down
71 synaptic DEPs
15%
260 DEPs Up
37 synaptic DEPs
14%
-log10(adj. P)
log2(FC)

Figure S3. P75NTR and photoreceptor degeneration pathway in the AD retina.

(**A**) Heatmaps of photoreceptor-relevant DEPs in the AD retina, organized by cellular compartment.

(**B**) Violin plots of selected photoreceptor-specific proteins quantified by MS. Individual patient values are presented (n = 6/group).

(**C**) Heatmaps of DEPs [$\log_2$(FC) and $-\log_{10}(P)$] in AD retina for select pathways linked to retinal degeneration. The heatmap on the left corresponds to the protein expression level in the 6 NC individuals and the 6 AD patients, standardized by unit variance scaling and generated in ClustVis. Clustering of DEPs was carried out manually based on their involvement in select pathways for visual clarity.

(**D**) Interaction network analysis (String v12.0) of DEPs in AD retina related to photoreceptor degeneration (yellow inner ring) and death receptor signaling (grey inner ring). Blue and red node fills indicate downregulated and upregulated DEPs in AD retina, respectively. The node size increases with the significance level of the difference in protein expression between AD patients and NC individuals.

(**E**) Heatmap of Pearson's correlation coefficients between p75NTR and DEPs in AD retina related photoreceptor degeneration. *adj. $P < 0.5$.

(**F**) Volcano plot showing the proportion of dysregulated synapse-associated proteins in AD versus NC control retinas. Synapse-associated proteins were extracted from SynGo v1.2 database.

For this figure, DEPs were defined by $|FC| > 1.2$ and adj. $P < 0.2$.

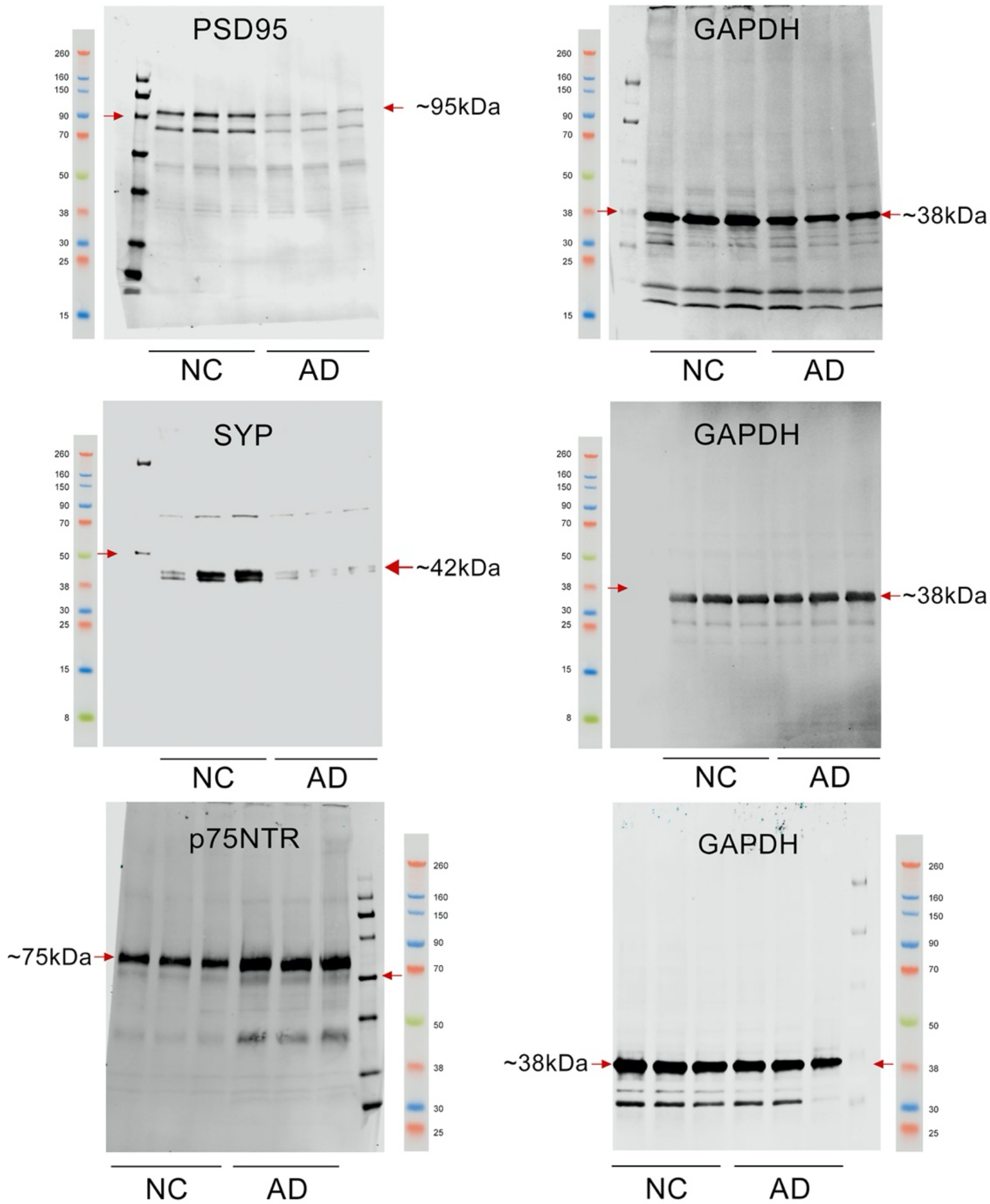

Figure S4. Full Western blots for retinal PSD95, Synaptophysin and p75NTR.

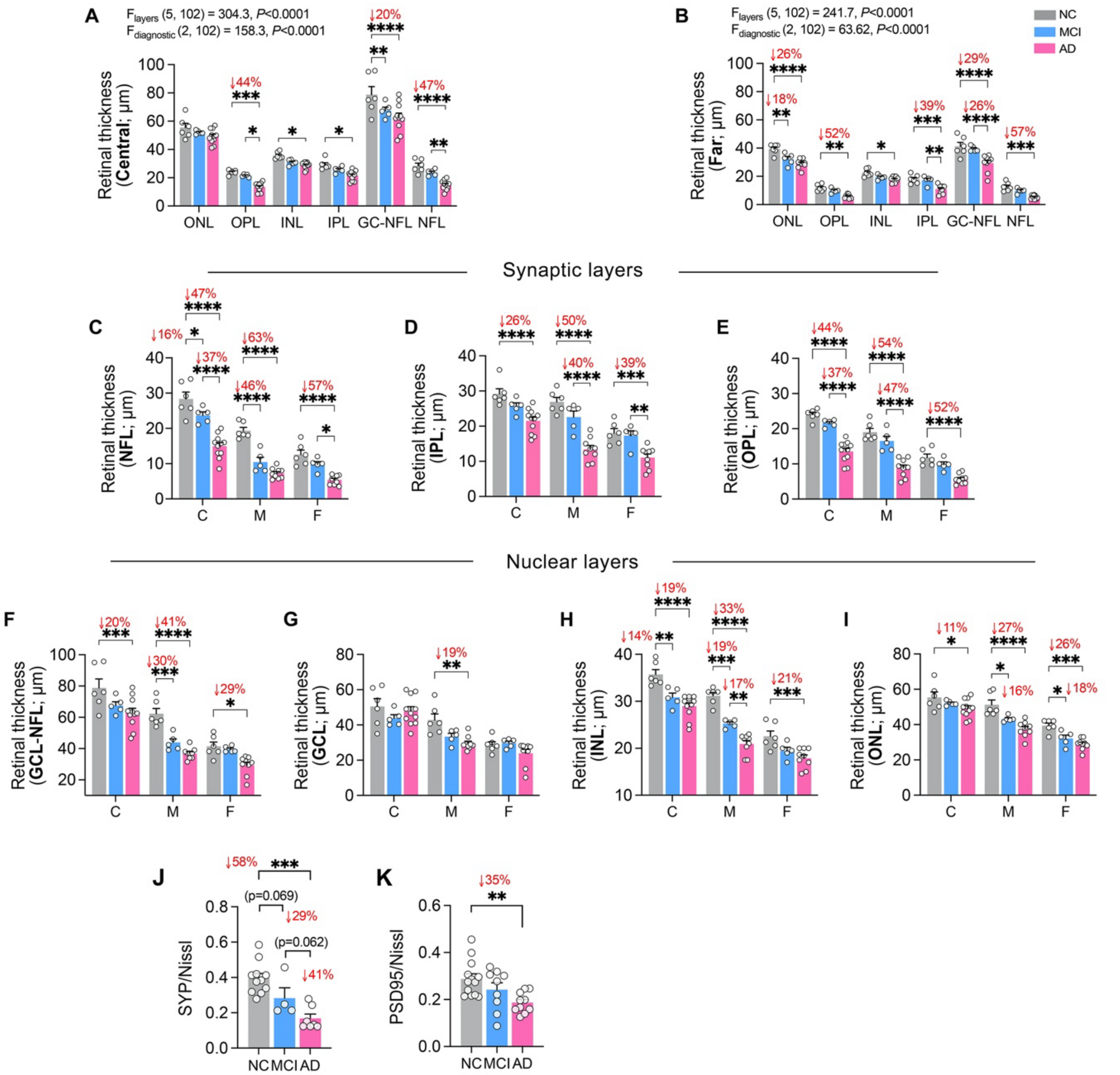

Figure S5. Regional and spatial retinal atrophy assessed by retinal thickness.

(A–I) Retinal thickness measurements across different regions and layers from a subset of individuals with NC (n =6), MCI (n = 5) and AD (n =9-11), in the central subregion (**A**) and far peripheral subregion (**B**). Thickness of specific retinal layers is shown for: NFL (**C**), IPL (**D**), OPL (**E**), GCL-NFL (**F**), GCL (**G**), INL (**H**), and ONL (**I**).

(J-K) Quantitative analyses of retinal SYP IR area (NC =11, MCI = 4, AD = 7) (**J**) and PSD95 IR area (NC =13, MCI = 9, AD =10) (**K**) normalized to Nissl % area.

Bar graphs display individual subjects (circles) as well as group means ± SEMs. *P < 0.05, **P < 0.01, ***P < 0.001, ****P < 0.0001, by two-sided unpaired t-test (in parenthesis), one-way or two-way ANOVA followed by Tukey's post hoc multiple comparison test. Percentage (%) changes are shown in red. NFL, Nerve fiber layer; GCL, ganglion cell layer; IPL, inner plexiform layer; INL, inner nuclear layer; OPL, outer plexiform layer; ONL, outer nuclear layer.

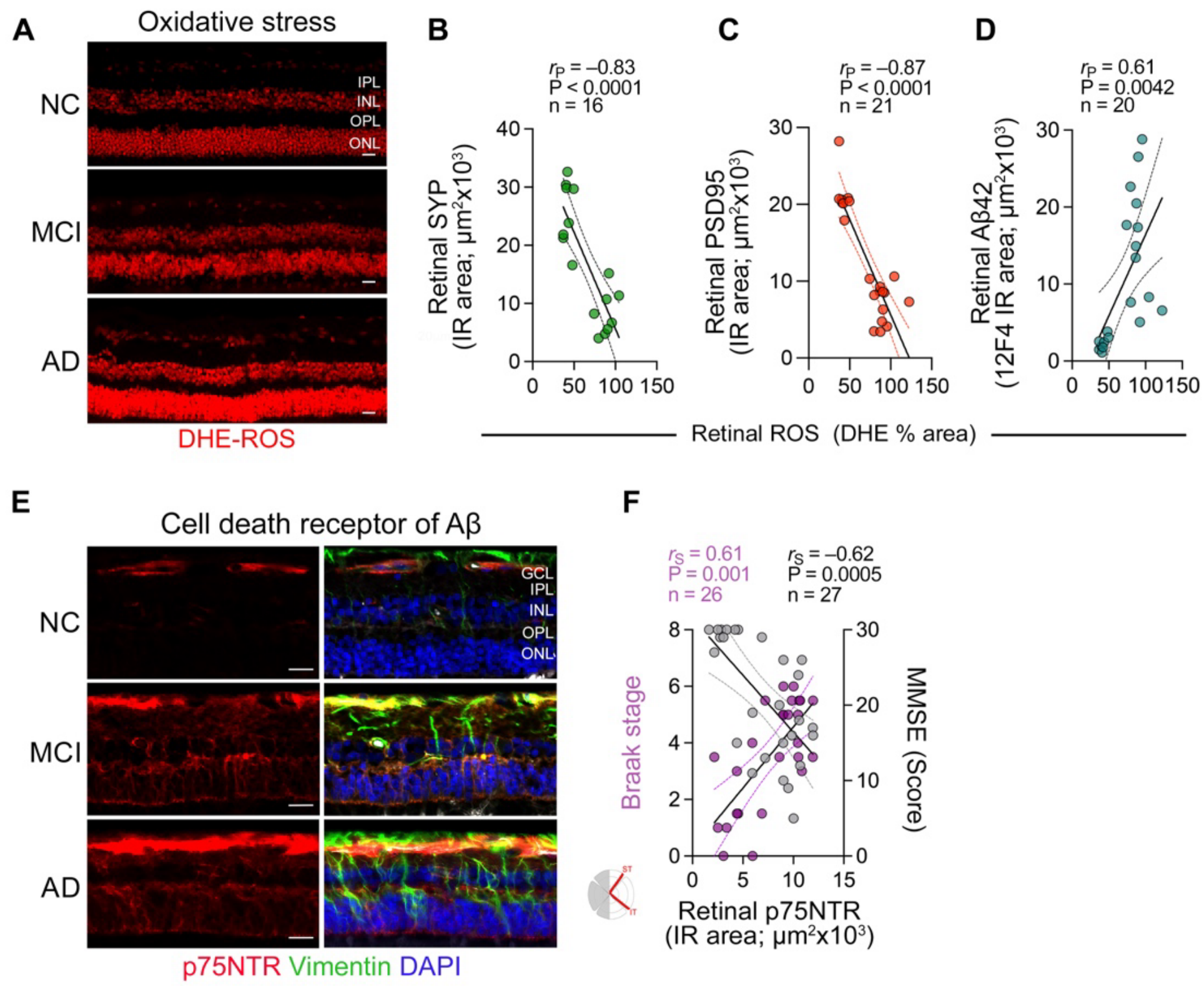

Figure S6. Extended analyses on retinal ROS and p75NTR.

(A) Representative dihydroethidium (DHE) staining of retinal reactive oxygen species (ROS) in NC, MCI, and AD retinas. Scale bars: 20 µm

(B-C) Pearson's correlation (r_P) between neuronal DHE$^+$ % area and retinal SYP (B), PSD95 (C) and 12F4-Aβ_{42} (D).

(E) Immunofluorescence of retinal cross-sections labeled for p75NTR (nerve growth factor receptor; red), vimentin (green), and nuclei (DAPI, blue) from individuals with NC, MCI and AD dementia.

(F) Spearman's correlation (r_S) between retinal p75NTR and Braak staging or MMSE scores.

GCL, ganglion cell layer; IPL, inner plexiform layer; INL, inner nuclear layer; OPL, outer plexiform layer; ONL, outer nuclear layer.

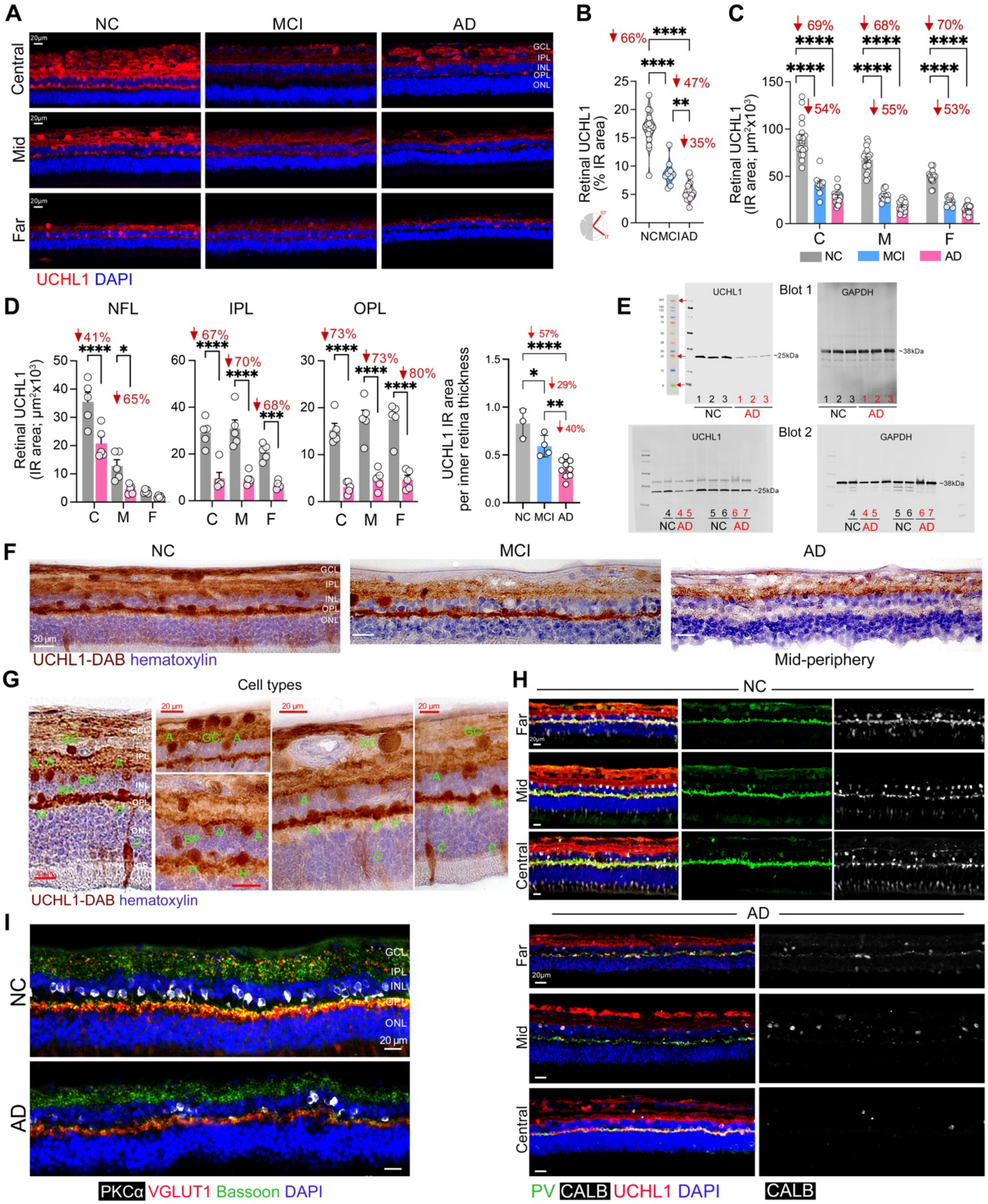

A
NC
MCI
AD
Central
Mid
Far
GCL
IPL
INL
OPL
ONL
UCHL1 DAPI
B
↓66%
****
****
↓47%
**
↓35%
Retinal UCHL1 (% IR area)
NC MCI AD
C
↓69%
↓68%
↓70%
↓54%
↓55%
↓53%
Retinal UCHL1 (IR area; µm²x10³)
NC
MCI
AD
D
NFL
IPL
OPL
↓41%
↓65%
↓67%
↓70%
↓68%
↓73%
↓73%
↓80%
↓57%
↓29%
↓40%
UCHL1 IR area per inner retina thickness
E
UCHL1
Blot 1
GAPDH
~25kDa
~38kDa
Blot 2
F
UCHL1-DAB hematoxylin
Mid-periphery
G
Cell types
UCHL1-DAB hematoxylin
H
I
PKCα VGLUT1 Bassoon DAPI
PV CALB UCHL1 DAPI
CALB

Figure S7. Extended data on retinal UCHL1.

(**A)** Representative fluorescence micrographs of retinal cross-sections immunolabeled for UCHL1 (red) and nuclei (DAPI, blue) in patients with MCI and AD versus NC control. Scale bars: 20 µm.

(**B**) Quantitative analysis of retinal UCHL1 % IR area in MCI (n = 9) and AD-dementia (n = 18) subjects compared with age and sex-matched NC controls (n = 16).

(**C**) Quantitative analyses of retinal UCHL1 IR in predefined retinal subregions, central (C), mid- (M), and far-periphery (F), in patients with MCI (n = 11) and AD-dementia (n = 21) versus age and sex-matched NC controls (n = 18).

(**D**) Quantitative analyses of retinal UCHL1 IR area in the NFL, IPL, OPL and normalized to inner retinal thickness, from a subset of patients with MCI (n = 4-5) and AD (n = 5-9) versus NC controls (n = 3-5).

(**E**) Full Western blots of retinal UCHL1 and GAPDH.

(**F-G**) Representative micrographs of retinal UCHL1 using peroxidase-based 3,3′diaminobenzidine (DAB) and hematoxylin counterstaining. Green labels indicate retinal cell types (GC, ganglion cell; A, amacrine; BP, bipolar; H, horizontal; C, cone) based on their localization within the retinal layers. Scale bars: 20 µm.

(**H**) Representative fluorescence micrographs of retinal cross-sections immunolabeled for Parvalbumin (PV, green), Calbindin (CALB, white), UCHL1 (red) and nuclei (DAPI, blue) in NC and AD retinas. Scale bars: 20 µm.

(**I**) Representative fluorescence micrographs of retinal cross-sections immunolabeled for PKCα (white), VGLUT1 (red), Bassoon (green) and nuclei (DAPI, blue) in NC and AD retinas. Scale bars: 20 µm.

The violin plot shows individual subjects (circles) with lower, median, and upper quartiles. Bar graphs display individual subjects as well as group means ± SEMs. *P < 0.05, **P < 0.01, ***P < 0.001, ****P < 0.0001, by one-way or two-way ANOVA followed by Tukey's or Šidák's post hoc multiple comparison test. Percentage (%) changes are shown in red. GCL, ganglion cell layer; IPL, inner plexiform layer; INL, inner nuclear layer; OPL, outer plexiform layer; ONL, outer nuclear layer.

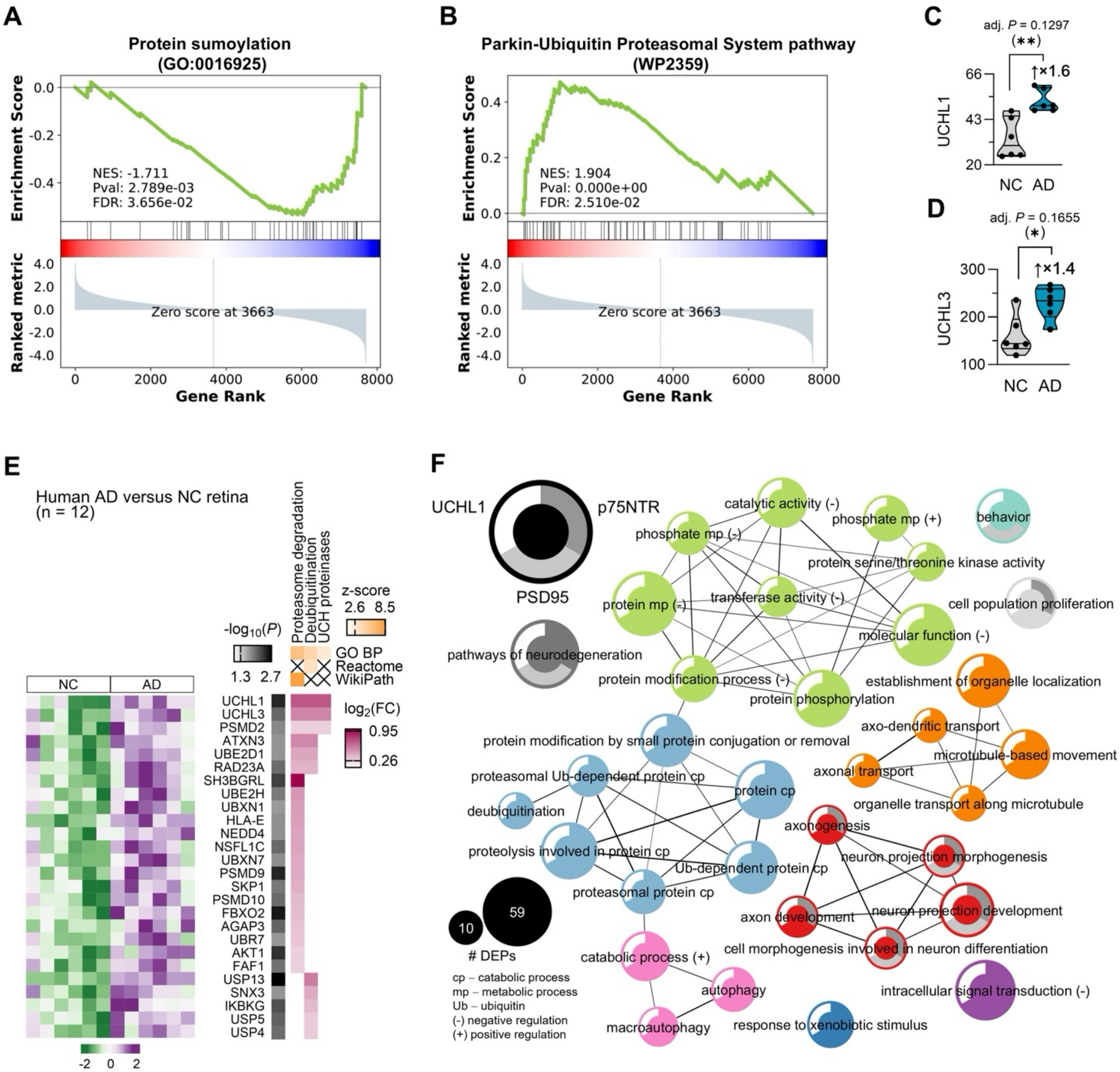
A
Protein sumoylation
(GO:0016925)
Enrichment Score
NES: -1.711
Pval: 2.789e-03
FDR: 3.656e-02
Ranked metric
Zero score at 3663
Gene Rank
B
Parkin-Ubiquitin Proteasomal System pathway
(WP2359)
Enrichment Score
NES: 1.904
Pval: 0.000e+00
FDR: 2.510e-02
Ranked metric
Zero score at 3663
Gene Rank
C
adj. P = 0.1297
(**)
↑×1.6
UCHL1
NC AD
D
adj. P = 0.1655
(*)
↑×1.4
UCHL3
NC AD
E
Human AD versus NC retina
(n = 12)
Proteasome degradation
Deubiquitination
UCH proteinases
z-score
2.6 8.5
-log10(P)
1.3 2.7
GO BP
Reactome
WikiPath
log2(FC)
0.95
0.26
NC
AD
UCHL1
UCHL3
PSMD2
ATXN3
UBE2D1
RAD23A
SH3BGRL
UBE2H
UBXN1
HLA-E
NEDD4
NSFL1C
UBXN7
PSMD9
SKP1
PSMD10
FBXO2
AGAP3
UBR7
AKT1
FAF1
USP13
SNX3
IKBKG
USP5
USP4
-2 0 2
F
UCHL1
p75NTR
PSD95
pathways of neurodegeneration
catalytic activity (-)
phosphate mp (+)
behavior
phosphate mp (-)
protein serine/threonine kinase activity
protein mp (-)
transferase activity (-)
cell population proliferation
molecular function (-)
protein modification process (-)
protein phosphorylation
establishment of organelle localization
protein modification by small protein conjugation or removal
axo-dendritic transport
proteasomal Ub-dependent protein cp
axonal transport
microtubule-based movement
protein cp
deubiquitination
organelle transport along microtubule
axonogenesis
proteolysis involved in protein cp
neuron projection morphogenesis
Ub-dependent protein cp
proteasomal protein cp
axon development
neuron projection development
59
10
DEPs
cell morphogenesis involved in neuron differentiation
catabolic process (+)
autophagy
intracellular signal transduction (-)
macroautophagy
response to xenobiotic stimulus
cp – catabolic process
mp – metabolic process
Ub – ubiquitin
(-) negative regulation
(+) positive regulation

Figure S8. UCHL1 and associated molecular pathways in the AD retina.

(**A-B**) GSEA enrichment plots for GO protein sumoylation enriched in our bottom-ranked dataset (downregulated in AD: NES=-1.711, FDR<0.05) (**A**) and for WikiPathway Parkin-ubiquitin proteasomal system enriched in our top-ranked dataset (upregulated in AD: NES=1.904, FDR<0.05) (**B**).

(**C-D**) Violin plots of the ubiquitin hydrolases, UCHL1 (**C**) and UCHL3 (**D**), quantified by MS in AD versus NC control retinas (n = 6/group).

(**E**) Heatmaps of DEPs [log2(FC) and -log10(P)] in AD retina for select pathways linked to deubiquitination. The heatmap on the left corresponds to the protein expression level in the 6 NC individuals and the 6 AD patients, standardized by unit variance scaling and generated in ClustVis. Clustering of DEPs was carried out manually based on their involvement in select pathways for visual clarity.

(**F**) Metascape network of dysregulated UCHL1-associated pathways in the AD retina. The size of the nodes represents the number of DEPs, while the thickness of edges represents the overlapping DEPs (association score) between GO terms. The inner ring indicates the co-involvement of PSD95 and p75NTR with UCHL1 in the molecular pathways. DEPs: |FC|>1.2 and P<0.05.

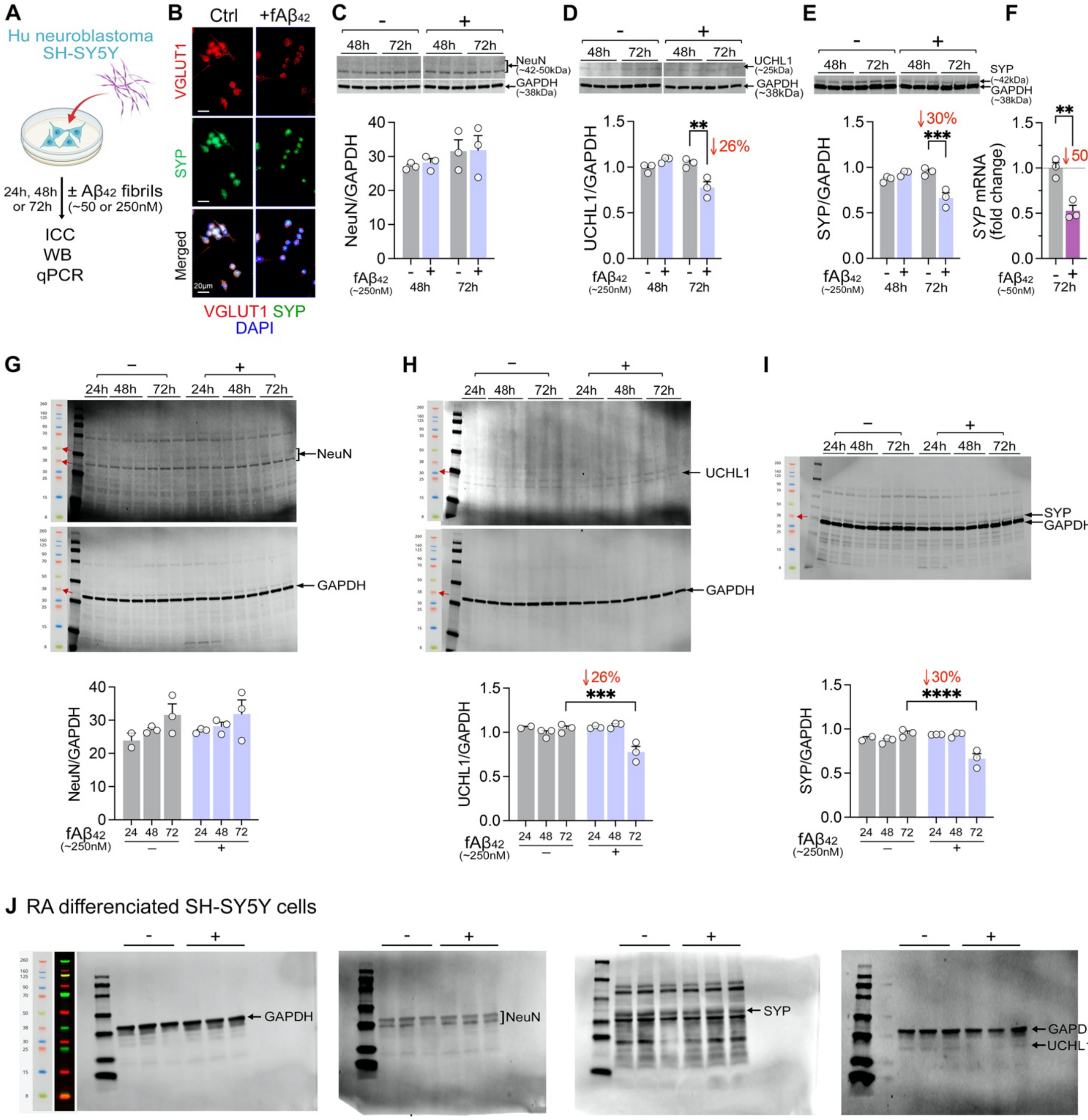

A
Hu neuroblastoma SH-SY5Y
24h, 48h or 72h
± Aβ42 fibrils (~50 or 250nM)
ICC
WB
qPCR
B
Ctrl
+fAβ42
VGLUT1
SYP
Merged
VGLUT1 SYP DAPI
C
NeuN (~42-50kDa)
GAPDH (~38kDa)
NeuN/GAPDH
D
UCHL1 (~25kDa)
UCHL1/GAPDH
↓26%
E
SYP (~42kDa)
SYP/GAPDH
↓30%
F
SYP mRNA (fold change)
↓50%
fAβ42 (~250nM)
fAβ42 (~50nM)
G
H
I
J RA differenciated SH-SY5Y cells

Figure S9. Extended data on SH-SY5Y cells.

(**A**) Experimental in vitro design: undifferenciated SH-SY5Y human (Hu) neurons were treated with fibrillar (f)$A\beta_{42}$ (50 or 250 nM) or left untreated (Ctrl) for 24-, 48-, or 72 h.

(**B**) Fluorescence micrographs of SH-SY5Y neurons ± 250 nM f$A\beta_{42}$ for 48 h, immunolabeled for VGLUT1 (red), synaptophysin (SYP; green), and nuclei (DAPI, blue). Scale bars: 20 µm

(**C-E**) Western blots and densitometric quantitative analyses of NeuN (C), UCHL1 (D), and SYP (E) normalized to GAPDH from neuronal cell pellets ± 250 nM f$A\beta_{42}$ for 24-, 48- and 72 h (n = 3/group).

(**F**) Quantitative real-time PCR analysis of *SYP* mRNA levels normalized to *GAPDH* mRNA in human SH-SY5Y neurons ± 50 nM f$A\beta_{42}$ for 72 h (n = 3/group).

(**G-I**) Full blots (from C-E panels) and corresponding densitometric quantitative analyses, of NeuN, UCHL1 and SYP normalized to GAPDH, at 24-, 48-, or 72 h time points (n = 2-3/group).

(**J**) Full Western blots of NeuN, SYP, UCHL1 and GAPDH from differentiated SH-SY5Y cell pellets ± 250 nM f$A\beta_{42}$ for 72 h (n = 3/group).

Individual data points (circles) as well as group means ± SEMs are shown. **P < 0.01, ***P < 0.001, ****P < 0.0001, by two-sided unpaired t-test or two-way ANOVA followed by Šidák's or Tukey's post hoc multiple comparison test. Percentage (%) changes are shown in red. Ctrl, control; f, fibrils; ICC, immunocytochemistry; qPCR, quantitative polymerase chain reaction; RA, retinoic acid; WB, Western blot.

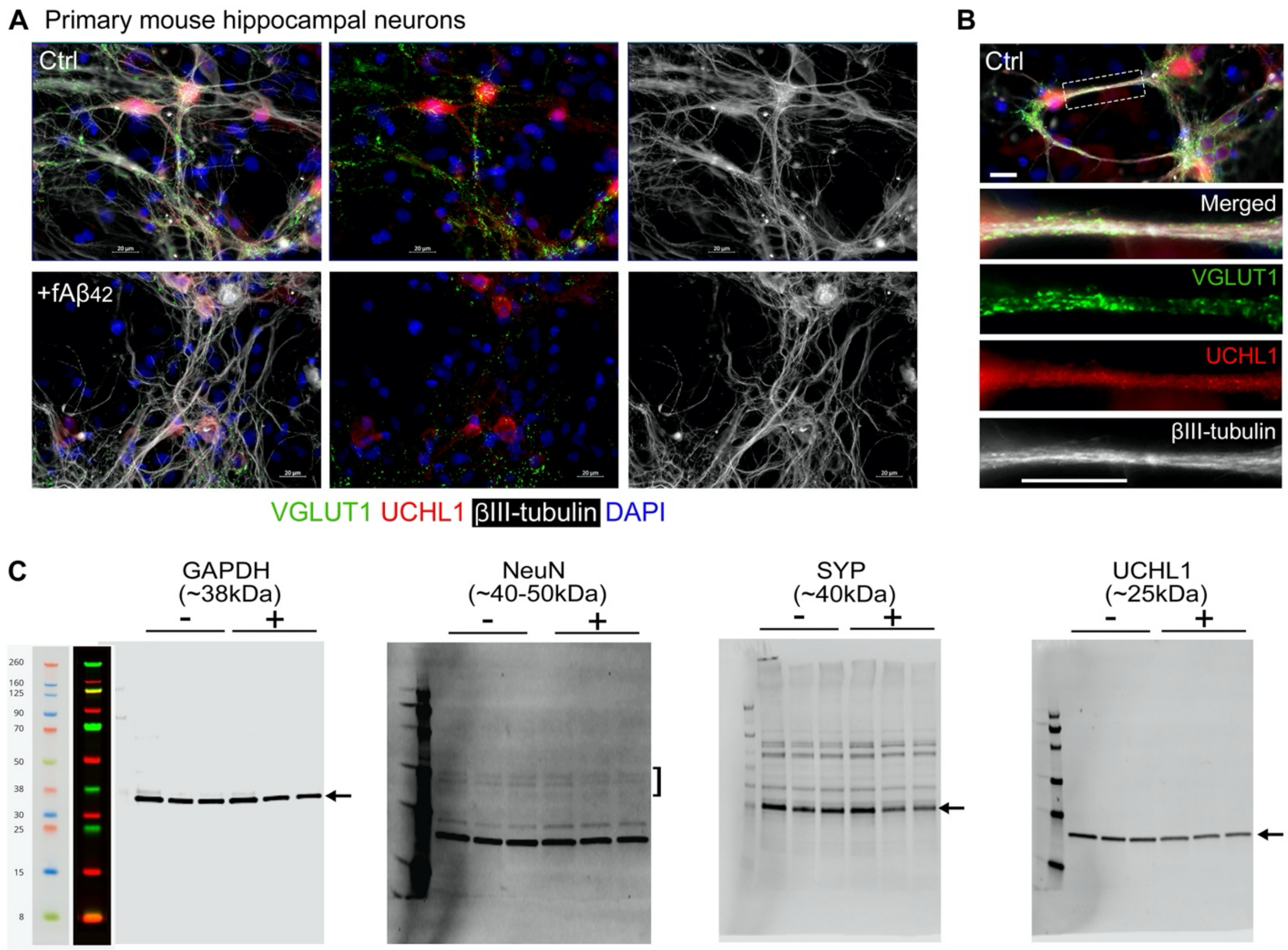

Figure S10. Extended data on primary mouse hippocampal neurons.

(**A-B**) Fluorescence micrographs and high magnification (B) of primary mouse hippocampal neurons ± 250 nM fAβ$_{42}$ immunolabeled with VGLUT1 (green), UCHL1 (red), βIII-tubulin (white), and nuclei (DAPI, blue). Scale bars: 20 µm

(**C**) Full Western blots of GAPDH, NeuN, SYP and UCHL1 from primary mouse hippocampal neurons pellets ± 250 nM fAβ$_{42}$ for 48 h (n = 3/group).

Ctrl, control; f, fibrils.

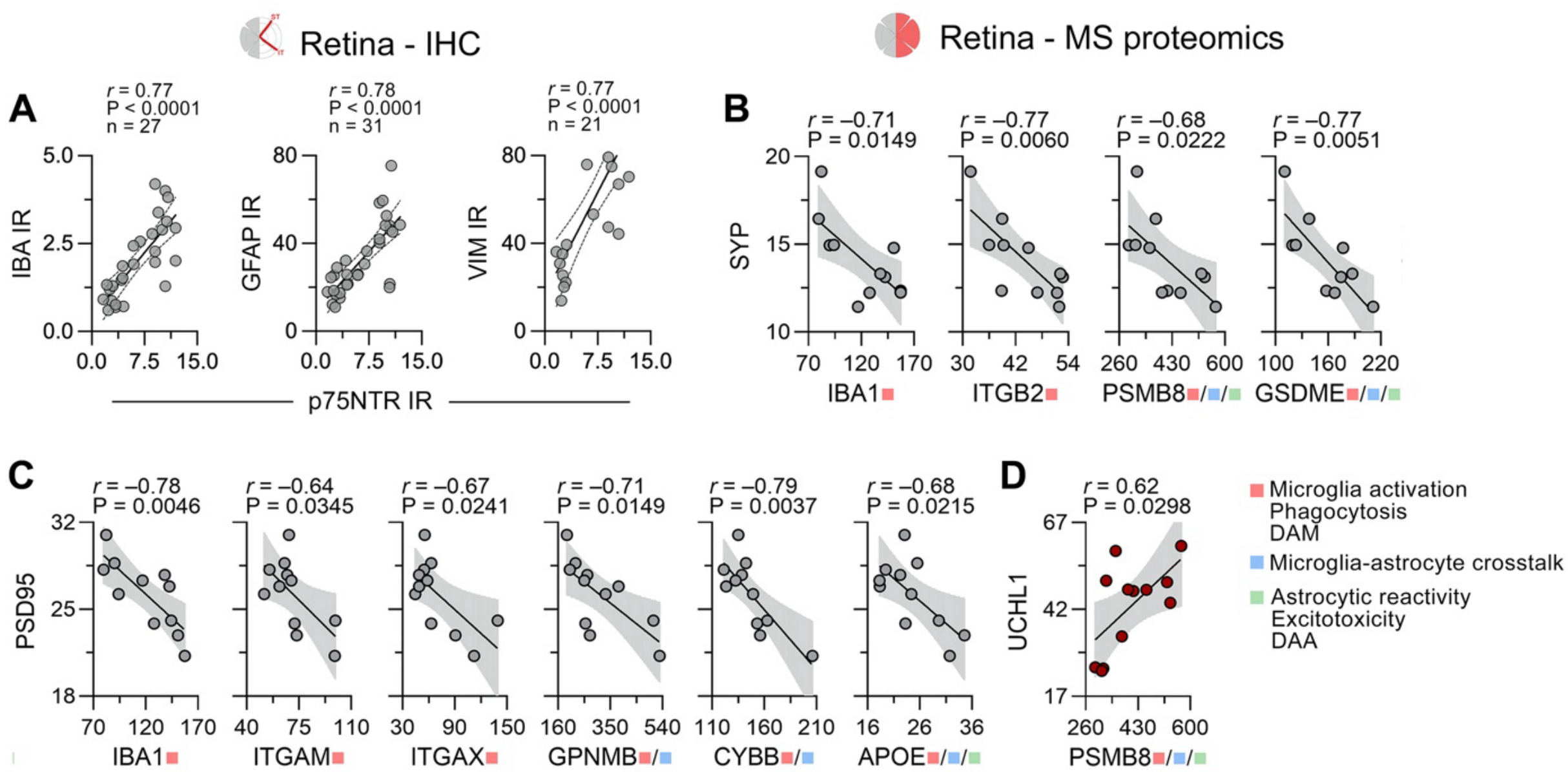

Figure S11. Extended data on synaptic alterations link to retinal p75NTR and retinal neuroinflammation.

(**A**) Representative pairwise Pearson's correlations, with unadjusted P values, between retinal p75NTR and retinal glial activation markers IBA1, GFAP and VIM.

(**B-D**) Representative proteomic pairwise correlations, with unadjusted P values, between synaptic and inflammatory markers: SYP versus IBA1/ITGB2/PSMB8/GSDME (B), PSD95 versus IBA1/ITGAM/ITGAX/GPNMB/CYBB/APOE (C) and UCHL1 versus PSMB8 (D).

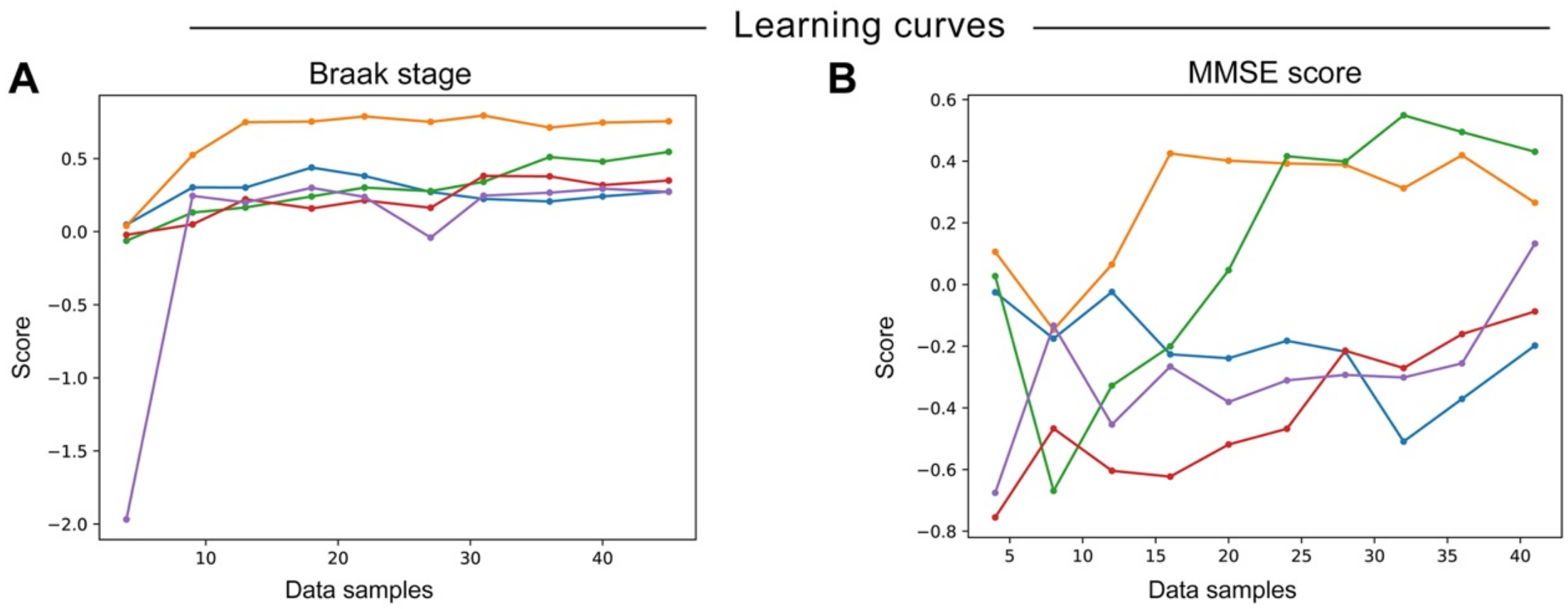

Figure S12. Extended data on machine learning prediction of AD severity.

(**A-B**) The Braak-stage model shows convergence across folds, indicating sufficient training data, whereas the MMSE model exhibits persistent cross-fold gaps, suggesting underfitting or data insufficiency: Learning curves for Braak stage (**A**) and MMSE (**B**) produced from Random Forest machine learning prediction.
